# Twist Grain Boundary Phases in Proper Ferroelectric Liquid Crystals Realm

**DOI:** 10.1002/advs.202508405

**Published:** 2025-07-13

**Authors:** Damian Pociecha, Jadwiga Szydlowska, Nataša Vaupotič, Katarzyna Kwiatkowska, Marijus Juodka, Julian Spiess, John MD Storey, Corrie T. Imrie, Rebecca Walker, Ewa Gorecka

**Affiliations:** ^1^ Department of Chemistry University of Warsaw Warsaw 02‐089 Poland; ^2^ Faculty of Natural Sciences and Mathematics University of Maribor Maribor 2000 Slovenia; ^3^ Jozef Stefan Institute Ljubljana 1000 Slovenia; ^4^ School of Natural and Computing Sciences University of Aberdeen Aberdeen AB24 3UE Great Britain

**Keywords:** chirality, liquid crystals, proper ferroelectrics, twist‐grain‐boundary

## Abstract

The twist‐grain‐boundary (TGB) phases, characterized by a periodic, helical arrangement of blocks made of polar smectic phases, SmA_F_ and SmC_F_, have been discovered. They are observed for rod‐like molecules with a strong longitudinal dipole moment, featuring an (S)‐2‐methylbutyl end group having only weak twisting power, and emerge below the antiferroelectric SmA_AF_ phase, where the lamellar structure is already well established. It is suggested that the structure is governed by electrostatic interactions amplified by weak chiral forces, in striking contrast to the mechanism of TGB phase formation found in non‐polar materials. The TGB phases exhibit selective light reflection in the visible range, while the value of electric polarization confirms an almost perfectly ordered dipole alignment.

## Graphical abstract

A new type of twist‐grain‐boundary (TGB) phases, with a periodic, helical arrangement of ferroelectric smectic blocks, are reported, formed by molecules with giant longitudinal dipole moment. They emerge below the antiferroelectric smectic phase, and their structure is governed by electrostatic interactions amplified by weak chiral forces. Polar TGB phases exhibit a photonic bandgap in the visible light range.

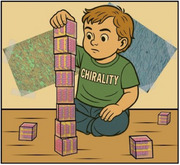

## 1 Introduction

In the field of liquid crystals (LC) the concepts of ferroelectricity and chirality are closely related. Ferroelectric properties of LC phases were discovered in the 1970s^[^ ^1^ ^]^ and, for a long time, were considered inherently linked to molecular chirality. The emergence of long‐range dipole order in smectic layers was thought to result from the lack of inversion symmetry elements in the system built of chiral molecules. In the tilted SmC^*^ phase molecular chirality not only induces spontaneous electric polarization but also causes the director, and thus polarization, to rotate between adjacent layers, producing the structural chirality of the phase. The discovery of ferroelectricity for achiral bent‐core molecules^[^ ^2^ ^]^ introduced a new perspective on the polarity/chirality relationship.^[^ ^3^ ^]^ In these systems, polarization appears due to steric interactions restricting molecular rotation. The resulting polarization vector, together with the tilt direction and layer normal, might define either a left‐ or right‐handed coordination system, thus a structural chirality emerges spontaneously even though the individual molecules themselves are achiral. This finding demonstrated that molecular chirality is not a prerequisite for the emergence of ferroelectricity in liquid crystals, but ferroelectricity and structural chirality are still related. The breakthrough discovery of proper ferroelectricity in the least‐ordered liquid crystalline phase – the nematic N_F_ phase,^[^ ^4^, ^5^, ^6^ ^]^ and later in smectic phases,^[^ ^7^, ^8^, ^9^, ^10^, ^11^ ^]^ seemed to decouple ferroelectricity from chirality; dipole‐dipole interactions alone were found sufficient to induce ferroelectric order. Molecular chirality still influences proper ferroelectric LC phases, e.g., transforming the N_F_ phase into its helical analogue, N_F_^*,^ similarly as observed for the non‐polar nematic phase. Importantly, the helical structure of the N_F_^*^ phase does not affect the value of local electric polarization, and likewise, polar order does not modify the helical pitch considerably.^[^ ^12^, ^13^, ^14^, ^15^ ^]^ For a while, in proper ferroelectric liquid crystals chirality and ferroelectricity appeared to be independent phenomena. However, in 2024, a pivotal discovery revealed that helicity can also emerge spontaneously as a mechanism to avoid bulk polarization, once again merging chirality and ferroelectricity in soft matter.^[^ ^16^, ^17^, ^18^, ^19^ ^]^

Here we will illustrate the behavior of a material made of weakly chiral but strongly polar molecules – prone to forming proper ferroelectric LC phases – and show that such a combination might lead to complex and unexpected structural arrangements: Twist Grain Boundary (TGB) phases. Mesogenic molecules tend to arrange themselves into well‐defined layers, while the molecular chirality causes a natural tendency of the molecules to twist. In general, lamellar structures expel the twist, however, in some systems, these two tendencies compete and the resulting TGB structure develops as a compromise between both ordering principles.^[^ ^20^ ^]^ The TGB structure consists of blocks of smectic layers that are separated by a periodic array of screw dislocations, mediating rotation of the smectic blocks. The axis of the resulting helix is perpendicular to the smectic layer normal, and often the pitch is of the order of visible light wavelength. The structure resembles that of type‐II superconductors, where magnetic flux lines penetrate the material in a periodic fashion.^[^ ^21^ ^]^ The TGB phase typically appears in strongly chiral materials and is usually found within a temperature range between the isotropic liquid or N^*^ and SmA or SmC phases, in which layer interactions are still weak.^[^ ^22^, ^23^, ^24^ ^]^ It was only rarely observed below a SmA phase.^[^ ^24^, ^25^ ^]^ Until now, no TGB phase has been reported in proper ferroelectric liquid crystals, although recently a twisted organization of discrete polar smectic blocks, inherited from the twisted state of the N_F_ phase, has been described.^[^ ^26^ ^]^

## 2 Structure Properties

**Figure 1:**
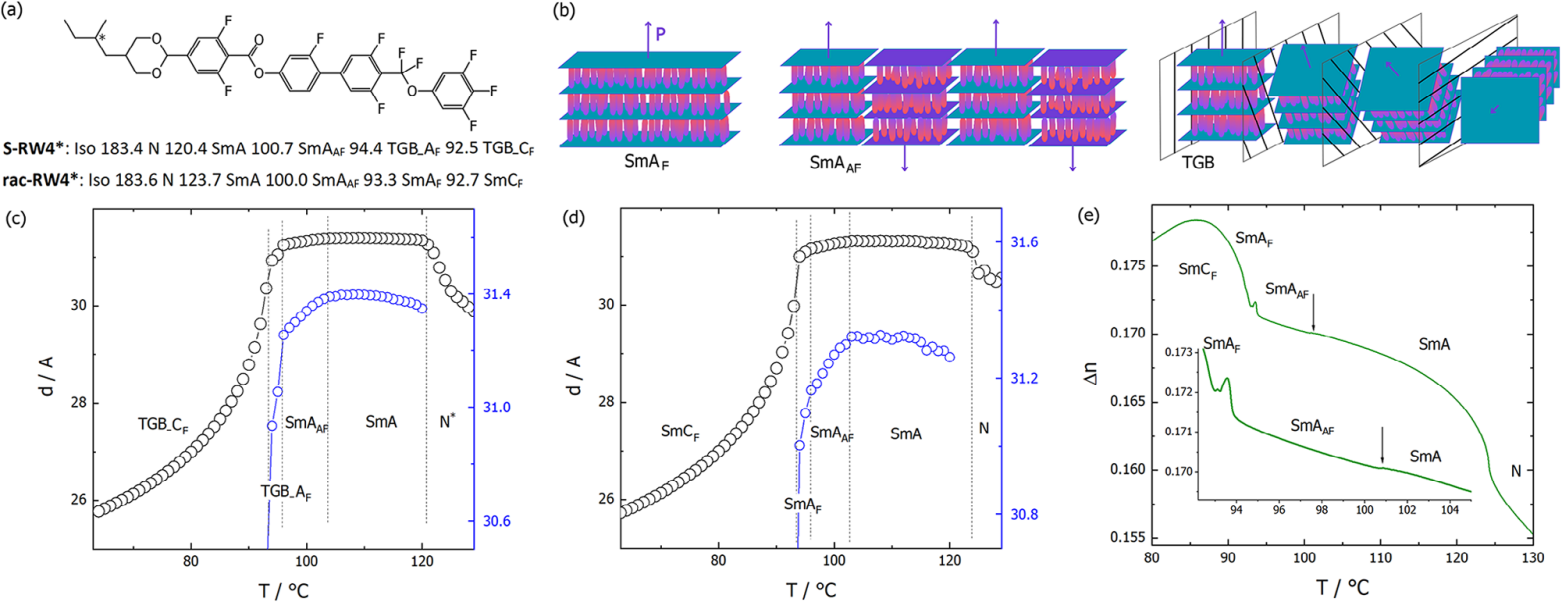
a) Molecular structure and phase transition temperatures determined by the DSC method for S‐enantiomer and racemic mixture of RW4^*^ compound. b) Models of ferroelectric SmA_F_, antiferroelectric SmA_AF_, and TGB phases, polar molecules are represented by ellipsoids with different color ends, arrows show the spontaneous polarization vector. Layer spacing versus temperature for c) S‐RW4^*^ and d) rac‐RW4^*^. e) Optical birefringence versus temperature for rac‐RW4^*^, measured in 1.6‐µm‐thick cell with planar anchoring. In the inset enlarged temperature range with SmA‐SmA_AF_‐ SmA_F_ phases. Note that in the SmC_F_ phase measured apparent optical retardance does not reflect actual birefringence changes, as the sample loses alignment with the formation of tilted domains (see Figure S51, Supporting Information).

The studied compound, RW4^*^, has a long, rigid mesogenic core with a substantial longitudinal dipole moment (≈12D), and a single terminal chain containing an asymmetric carbon atom (**Figure** **1**). The material is a modification of the previously studied compound **JK104**,^[^ ^17^ ^]^ with a non‐branched terminal chain, which showed a sequence of non‐polar and proper ferroelectric phases (denoted by subscript F): N – SmA – SmA_F_ – SmC_F_. Notably, shorter **JK10*n*** homologues formed the spontaneously helical ferroelectric nematic phase, N_TBF_. Both the optically pure enantiomer (S‐RW4^*^) and racemic mixture (rac‐RW4^*^) were studied, and they showed similar phase transition temperatures.

For both materials X‐ray diffraction (XRD) studies revealed a nematic phase and a sequence of three orthogonal smectic phases (SmA‐type with liquid like in‐plane order, Figure S49, Supporting Information), with transitions between them marked as slight changes in the slope of the layer spacing – temperature dependence (Figure 1c,d). On further cooling a strong decrease of layer thickness was observed, confirming formation of the tilted smectic C phase. There is no difference in layer spacing between the enantiomer and the racemic mixture. For rac‐RW4^*^, for which uniformly aligned samples could be obtained between glass plates treated for planar anchoring, the phase transitions were also tracked by optical birefringence, Δ*n*, changes. At the N‐SmA phase transition there is a step‐like increase of Δ*n*, and subsequent transitions between orthogonal smectic phases are marked by weak changes of birefringence, pointing to only small variations of the orientational order (Figure 1e).

The two highest‐temperature SmA‐type phases of rac‐RW4^*^ give a homeotropic texture when observed in free‐standing films or in cells with homeotropic anchoring, with no changes visible at the phase transition between them. Apparently, in both phases, the layers are oriented with the layer normal perpendicular to the sample surface. In the lowest temperature orthogonal smectic phase, the samples start to lose the homeotropic texture, and small wrinkle‐like defects develop (Figure S50, Supporting Information). On further cooling toward the SmC phase, the texture rebuilds completely, and a strongly birefringent texture develops, evidencing a book‐shelf geometry of layers. Such a behavior can be explained assuming that the lowest temperature SmA phase becomes ferroelectric, with the polarization vector along the layer normal. For an axially polar structure, there is a strong tendency to escape from homeotropic orientation of layers, even in the free suspended film samples, to avoid the charges at the film surface.^[^ ^27^ ^]^ Thus, based on the results of XRD and optical studies, one can speculate that the smectic phases in rac‐RW4^*^ appear on cooling in a sequence: non‐polar SmA, antiferroelectric SmA_AF_ with polarization compensated by the formation of separate blocks with antiparallel orientation (Figure 1b), ferroelectric SmA_F_ and SmC_F,_ with the onset of polar order in the SmA_AF_ phase. It should be noticed that birefringence, measured in planar cell, at SmA‐ SmA_AF_ phase transition (Figure 1e) shows the small step down (of order 10^−4^), which can be attributed to small splay of director, and thus polarization, by no more than 1–2 degrees at the boundaries of the antiferroelectric grains (see Supporting Information).

**Figure 2:**
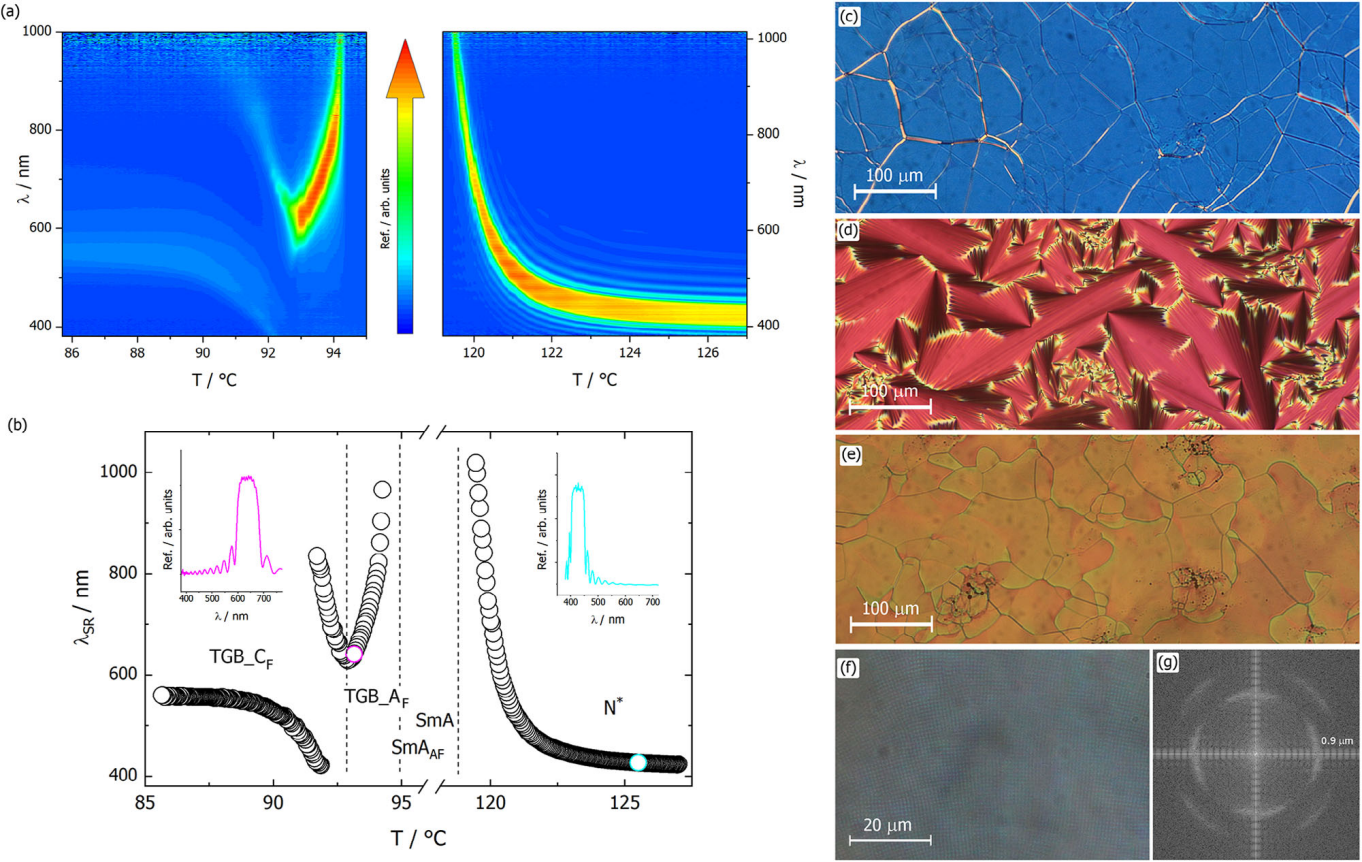
a) 2D plot showing temperature evolution of selective reflection bands in N^*^ phase (right) and TGB phases (left) for S‐RW4^*^ compound. b) Selective reflection wavelength, λ_SR_, versus temperature; in the insets reflection versus wavelength for chosen temperatures in N^*^ (right) and TBG_A_F_ (left) phases. Optical textures of c) N^*^ d) SmA_AF_, e) TGB_A_F_, and f) TGB_C_F_ phases. In TGB_C_F_ phase a square pattern texture is observed, the Fourier transform of which is presented in (g).

In cells with planar anchoring, the enantiomeric S‐RW4^*^ material shows a Grandjean texture in N^*^ phase (helical axis perpendicular to the cell surface) with the helical pitch in visible range (Figure S52, Supporting Information) and fan texture in the SmA and SmA_AF_ phases (**Figure** **2**; Figure S53, Supporting Information). The sample undergoes a complete reorganization in the lower temperature phases, typical for the formation of the TGB‐type phase (Figure 2e). In planar geometry, in the TGB phase, the Grandjean texture reappears, and the helical structure gives selective reflection of light in the visible range. On lowering the temperature, judging from the sequence of colors, the helix winds, and the phase transition between TGB_A_F_ and TGB_C_F_ phases is marked by the appearance of two reflection bands in the visible range (Figure 2). The selective reflection bands in the TGB_C_ phase are asymmetric and become less intense with lowering temperature.

**Figure 3:**
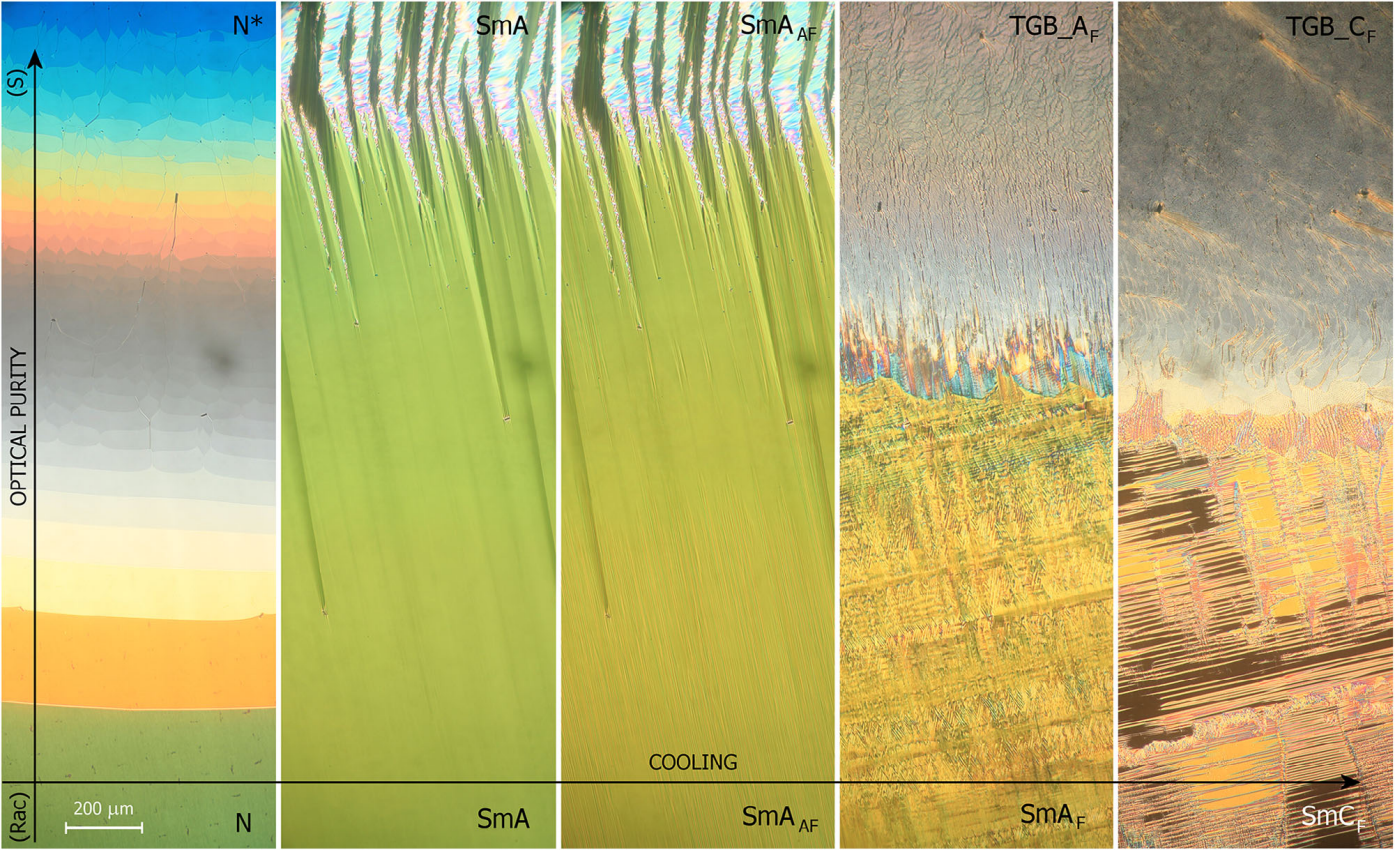
Temperature evolution of the optical textures in 3‐µm‐thick cell with planar anchoring, the area is presented in which optically pure S‐RW4^*^ (top) is in contact with racemic mixture rac‐RW4^*^ (bottom).

A contact cell, in which the enantiomer and racemic mixture form the diffused region with a gradual change of optical purity, shows that the TGB_A_F_ phase in S‐RW4^*^ corresponds to the temperature range of the SmA_F_ phase in rac‐RW4^*^, and the TGB_C_F_ phase to the SmC_F_ phase (**Figure** **3**).

In the temperature range corresponding to the TGB_C_F_ phase a square grid pattern develops.^[^ ^28^ ^]^ This is usually taken as evidence for the TGB_C^*^ structure (proposed by Renn^[^ ^29^ ^]^), in which, in addition to the helical superstructure of layer blocks, each block exhibits the director helix of SmC^*^ phase, with the helical axes being mutually perpendicular.

## 3 Polar Properties

To confirm the polar nature of the LC phases, the Second Harmonic Generation (SHG) activity was monitored, which is inherent to materials with a non‐centrosymmetric structure and is often used to prove the polar nature of a phase.^[^ ^30^, ^31^, ^32^ ^]^ The experiments were conducted in planar cells: in this geometry, a strong SHG signal is expected as the spontaneous polarization is perpendicular to the light propagation direction. In the racemic mixture, an SHG signal appears in the SmA_F_ and SmC_F_ phases. The enantiomeric material was SHG silent in all phases because, as expected, the global polarization in the TGB_A_F_ and TGB_C_F_ phases is canceled by the helical structure. However, under an electric field, above some critical value (the cell with in‐plane electric field was used) the SHG signal is observed already in the SmA_AF_ phase, clearly showing the switching from an antiferroelectric non‐SHG active to a ferroelectric, SHG active state (Figure 3; Figure S55, Supporting Information). In the SmA_AF_, TGB_A_F_, and TGB_C_F_ phases, a strong SHG signal appears upon application of an electric field, and its intensity increases with lowering temperature (Figure S56, Supporting Information).

**Figure 4:**
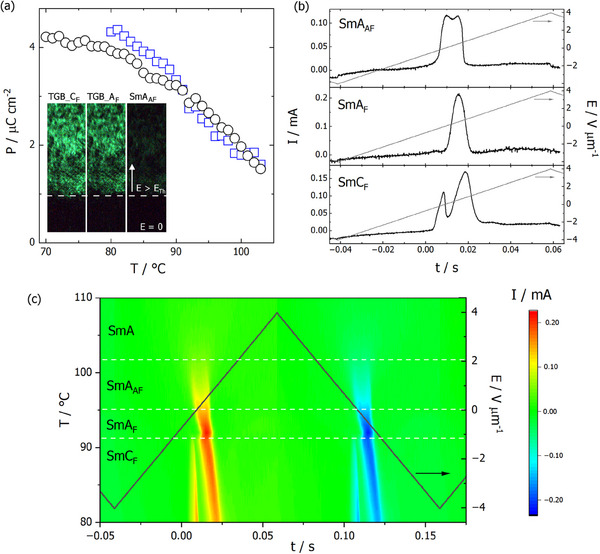
a) Electric polarization versus temperature for rac‐RW4^*^ (blue squares) and S‐RW4^*^ (black circles). In the inset, the SHG‐microscopy images taken in SmA_AF_, TGB_A_F_, and TGB_C_F_ phases in the cell with in‐plane electrodes. The SHG active areas (top part) are between electrodes, thus are exposed to the electric field, the intensity of the SHG signal reflects differences in electric polarization, SHG silent areas (bottom part) are on the electrode. b) Switching current recorded under application of triangular‐wave ac electric field in SmA_AF_, SmA_F_, and SmC_F_ phases of rac‐RW4^*^. c) Temperature evolution of repolarization current peaks in smectic phases of rac‐RW4^*^, evidencing very slight changes in the threshold field for polarization switching.

The electric polarization was determined by measuring the switching current resulting from the application of ac electric field. In the SmA_F_ (or TGB_A_F_) phase, a single current peak per half of the electric field cycle is found, while in the SmA_AF_ phase a symmetric double peak is registered – consistent with the antiferroelectric nature of the phase (**Figure** **4**). The modified electric field cycle (with two consecutive triangular wave functions of the same polarity) confirmed this assumption; the repolarization current peaks appear at each rise/fall of the electric field, with the antiferroelectric ground state restored at zero field (Figure S57, Supporting Information). In the SmC_F_ (and TGB_C_F_) phase, the current peak again becomes double, but asymmetric, due to the complex nature of the switching in this phase that involves both repolarization and changes of the tilt and induction of orthogonal structure under the electric field.^[^ ^10^, ^11^ ^]^ The spontaneous electric polarization calculated from the current peak gradually increases and reaches ≈4.5 µC cm^2^ (Figure 4a), showing nearly perfect ordering of the dipole moments.

Dielectric spectroscopy measurements revealed a weak relaxation mode in the SmA and SmA_AF_ phases. This mode softens with decreasing temperature, as evidenced by a decrease in relaxation frequency and an increase in mode strength, indicating the onset of polar order in the SmA_F_ (TGB_A_F_) phase. In the SmA_AF_ phase, a second relaxation mode starts to appear at a higher frequency, though the dielectric response remains relatively weak compared to that in the lower‐temperature phases, possibly hinting at tilt fluctuations starting to develop on approaching the SmC_F_ (TGB_C_F_) phase (Figure S58, Supporting Information). In tilted phases, the dielectric response is substantial, characteristic of proper ferroelectric liquid crystalline phases with strong fluctuations of polar order direction.^[^ ^10^ ^]^

## 4 Conclusions

Summarizing the results, for the studied material with a strong longitudinal dipole moment, regardless of its optical purity, the higher‐temperature phases are non‐polar nematic (N), non‐polar orthogonal SmA, and antiferroelectric SmA_AF_. In the lower temperature range, the SmA_F_ and SmC_F_ phases, which appear in the racemic mixture, are in an enantiomerically pure material transformed into a twist grain boundary superstructure. This phase sequence, with a large temperature range of smectic phases preceding the TGB structure, is in strong contrast to non‐polar materials, where TGB phases typically emerge in a temperature range directly below the nematic phase, when the layer structure is weak. Moreover, for the system studied here, TGB phases emerge for mesogens featuring an (S)‐2‐methylbutyl end group, which is considered to have only weak helical twisting power,^[^ ^33^ ^]^ while for non‐polar materials, a significantly stronger twisting power is required to induce a TGB phase formation. This suggests that the mechanism driving the TGB structure in the studied material is fundamentally different and is influenced by polar interactions. The observed transitions can be accounted for by a continuous phenomenological model. The free energy includes elastic contributions and Landau terms describing the phase transitions from the nematic to the smectic A phase (at temperature *T_NS_*), from smectic A to smectic C phase (at *T_AC_*) and the transition to the polar phase (at *T_P_*). Details of the model are given in the Supporting Information. The most important terms in the elastic energy are due to the splay and twist deformation of the nematic director. In a racemic mixture, there is no tendency for a spontaneous twist, while it appears in an enantiomer. In both the racemic mixture and optically pure enantiomer, a spontaneous splay is favourable in polar phases due to the flexoelectric effect. If *T_NS_* > *T_P_* > *T_AC_*, the model describes transitions from the apolar nematic phase to apolar smectic A phase, then to the polar smectic A phase and finally to the polar smectic C phase upon the reduction of temperature. At the onset of the polar order (*T* < *T_P_*), polarization splay becomes favourable, which is directly related to the splay of the nematic director. In the SmA phase, splay of the director can be achieved by undulation of smectic layers, which comes with no energy penalty, because constant splay does not affect the smectic layer thickness. However, a favourable splay cannot be achieved everywhere, thus, regions of favourable splay with the up and down polarization interchange, being separated by regions (walls) without polar order (see Figure S59, Supporting Information). A phase transition from the SmA to antiferroelectric SmA_AF_ phase is thus observed. From the birefringence measurements for the studied material, the splay angle is estimated to be ±1.5 degree along the polar block. At temperatures close to *T_P_*, melting of the polar order is not energetically costly, but the energy cost increases with decreasing temperature as (*T_P_* − *T*)^2^. Thus, at some temperature *T_F_* the cost of the wall with no polar order becomes the same as the cost of the wall with polar order and unfavourable splay. This leads to a phase transition to the SmA_F_ phase where the neighbouring blocks of favourable splay have the same direction of polarization. With a further reduction of temperature, a transition to the ferroelectric SmC_F_ phase is obtained. The reason that the SmA_F_ and SmC_F_ phases are observed in a racemic mixture but not in an enentiomer lies in the fact that a chiral material prefers a spontaneous twist, which is impossible to accommodate along the layer without changing its thickness. In a chiral material, the transition from SmA_AF_ to the polar TGB_A_F_ phase is observed, because the walls between smectic blocks in the TGB structure can accommodate a favourable twist and unfavourable splay; the energy price of the latter being compensated by the energy gain due to the former. By assuming a constant splay of polarization within a block, the amplitude of the splay being small (as only a very weak change of optical birefringence was detected at the SmA‐SmA_AF_ phase transition), we estimated the order of magnitude of the blocks’ size in polar phases to be 10 nm (see Supporting Information), which is consistent with the widths of the blocks measured in the ordinary TGB_A phase.^[^ ^34^ ^]^ The width of the block is below the light diffraction limit, resembling blocks in the SmZ_A_ (N_x_) phase,^[^ ^35^, ^36^ ^]^ which are built of ferroelectric nematic domains. However, experimental confirmation of the blocks’ size and how they are connected remains an open question. The TGB and SmA_AF_ structures develop in a temperature range where the lamellar order is already strong, therefore we can disregard the possibility that the blocks’ interfaces are molten.^[^ ^37^ ^]^

## Conflict of Interest

The authors declare no conflict of interest.

## Supporting information



Supporting Information

## Data Availability

The data that support the findings of this study are available from the corresponding author upon reasonable request.
