# Supplementary material for: Twist Grain Boundary Phases in Proper Ferroelectric Liquid Crystals Realm
Source: Adv Sci (Weinh). 2025 Jul 13;12(38):e08405. doi: 10.1002/advs.202508405 (PMC12520520; doi:10.1002/advs.202508405)
Supplement: Supplementary file 1 — Supporting Information [file ADVS-12-e08405-s001.pdf]

ADVANCED SCIENCE
Open Access

Supporting Information

for *Adv. Sci.*, DOI 10.1002/advs.202508405

Twist Grain Boundary Phases in Proper Ferroelectric Liquid Crystals Realm

*Damian Pociecha, Jadwiga Szydlowska, Nataša Vaupotič, Katarzyna Kwiatkowska, Marijus Juodka, Julian Spiess, John MD Storey, Corrie T. Imrie, Rebecca Walker and Ewa Gorecka**

Twist Grain Boundary phases in proper ferroelectric liquid crystals realm

*Damian Pociecha, Jadwiga Szydlowska, Nataša Vaupotič, Katarzyna Kwiatkowska, Marijus Juodka, Julian Spiess, John MD Storey, Corrie T Imrie, Rebecca Walker, Ewa Gorecka**

**Synthetic Procedures and Structural Characterisation**	**1**
**Experimental**	**36**
**Additional Results**	**38**
**Phenomenological Model**	**49**
**References**	**54**

Synthetic Procedures and Structural Characterisation

Reagents All reagents and solvents that were available commercially were purchased from Sigma Aldrich, Fisher Scientific or Fluorochem and were used without further purification unless otherwise stated.

Thin Layer Chromatography Reactions were monitored using thin layer chromatography, and the appropriate solvent system, using aluminium-backed plates with a coating of Merck Kieselgel 60 F254 silica which were purchased from Merck KGaA. The spots on the plate were visualised by UV light (254 nm) or by oxidation using either a potassium permanganate stain, iodine or p-anisaldehyde dip.

Column Chromatography For normal phase column chromatography, the separations were carried out using silica gel grade 60 Å, 40-63 μm particle size, purchased from Fluorochem and using an appropriate solvent system.

Structure Characterisation All final products and intermediates that were synthesised were characterised using ^{1}H NMR, ^{13}C NMR and infrared spectroscopies. The NMR spectra were recorded on a 400 MHz Bruker Avance III HD NMR spectrometer. The infrared spectra were recorded on a Perkin Elmer Spectrum Two FTIR spectrometer with an ATR diamond cell.

The synthetic routes to the (S)-enantiomer and racemic versions of **RW4*** were identical, apart from utilisation of (S)-1-bromo-2-methylbutane and 1-bromo-2-methylbutane, respectively, in Step 1, and summarised in ***Scheme 1***.

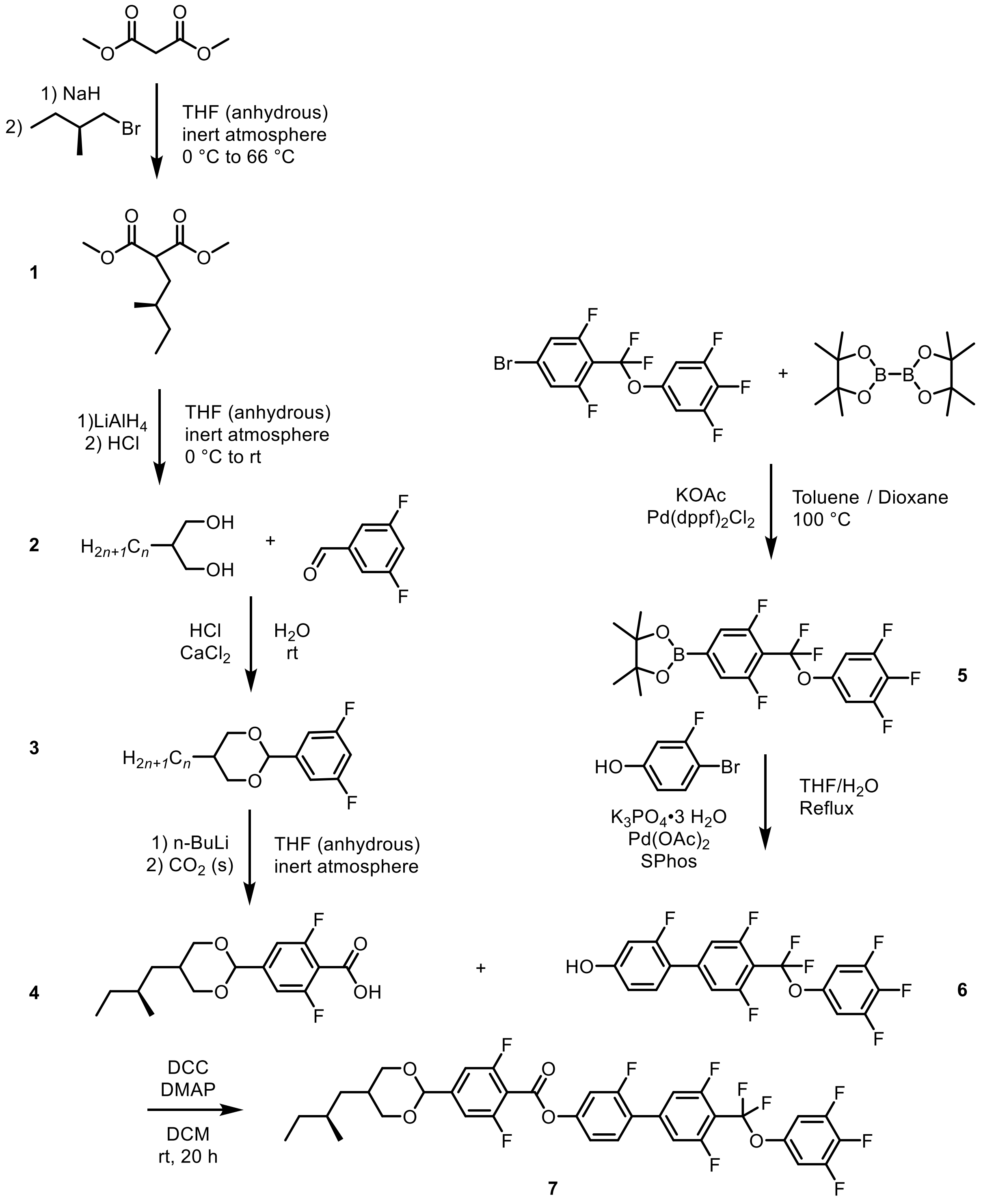

*Scheme S1. Synthetic route to new materials reported. Final product 7 was obtained as S-enantiomer (**S-RW4***) and as racemic mixture (**rac-RW4***)*

Dimethyl 2-[(2S)-2-methylbutyl]propanedioate (1(S)) /

Dimethyl 2-(2-methylbutyl)propanedioate (1(rac))

NaH (1.1 eq) was placed in a 2-necked RBF with a magnetic stirrer under an argon atmosphere. Hexane (3 x 4 mL) was added with a syringe, the mixture was stirred, followed by a removal of the hexane - mineral oil solution using a syringe. Then, anhydrous THF (15 mL) was added to the RBF using a syringe, and the reaction mixture cooled down to 0 ^{o}C using an ice bath. Dimethylmalonate (1.1 eq) was then added dropwise using a syringe and the reaction was left to stir for 30 minutes until all hydrogen evolution had ceased. Using the same technique, (S)-1-bromo-2-methylbutane* (1 eq) was added to the reaction mixture. The ice bath was then removed, and the reaction mixture stirred at reflux for 8h. The reaction was quenched using HCl (1 N), and the product was extracted using of Et_2O (3 x 10 mL). Combined organic layers were washed with water (2 x 30 mL), brine (2 x 30 mL) and dried over $MgSO_4$. The solvent was removed *in vacuo* and flash chromatography (8:2 PE/EtOAc, Rf = 0.48) was performed to yield the pure compound as a clear liquid.

*Procedure for **1(rac)** used 1-bromo-2-methylbutane.

1(S):

Yield: 62.8 %

^{1}**H NMR** (400 MHz, $CDCl_3$): δ ppm = 3.68 – 3.59 (6H, m, -(O-$\underline{CH_3}$)$_2$), 3.42 – 3.32 (1H, m, -CO-$\underline{CH}$(-CH_2)-CO-), 1.91 – 1.82 (1H, m, -CH-$\underline{CH}$(-H)-CH-), 1.63 – 1.54 (1H, m, -CH-CH($\underline{-H}$)-CH-), 1.33 – 1.18 (2H, m, -CH-$\underline{CH_2}$-CH_3), 1.12 – 1.02 (1H, m, -CH(-H)-$\underline{CH}$(-CH_3)-CH_2-), 0.81 – 0.73 (6H, m, -CH(-$\underline{CH_3}$); -CH_2-$\underline{CH_3}$).

^{13}**C NMR** (101 MHz, $CDCl_3$): δ ppm = 170.09, 169.96, 52.33, 52.28, 49.69, 35.50, 32.37, 29.14, 18.57, 10.96.

IR (ν_{max}/cm^{-1}): 2957 (sp^3 C-H stretch), 2933 (sp^3 C-H stretch), 2877 (sp^3 C-H stretch), 1734 (C=O stretch, ester).

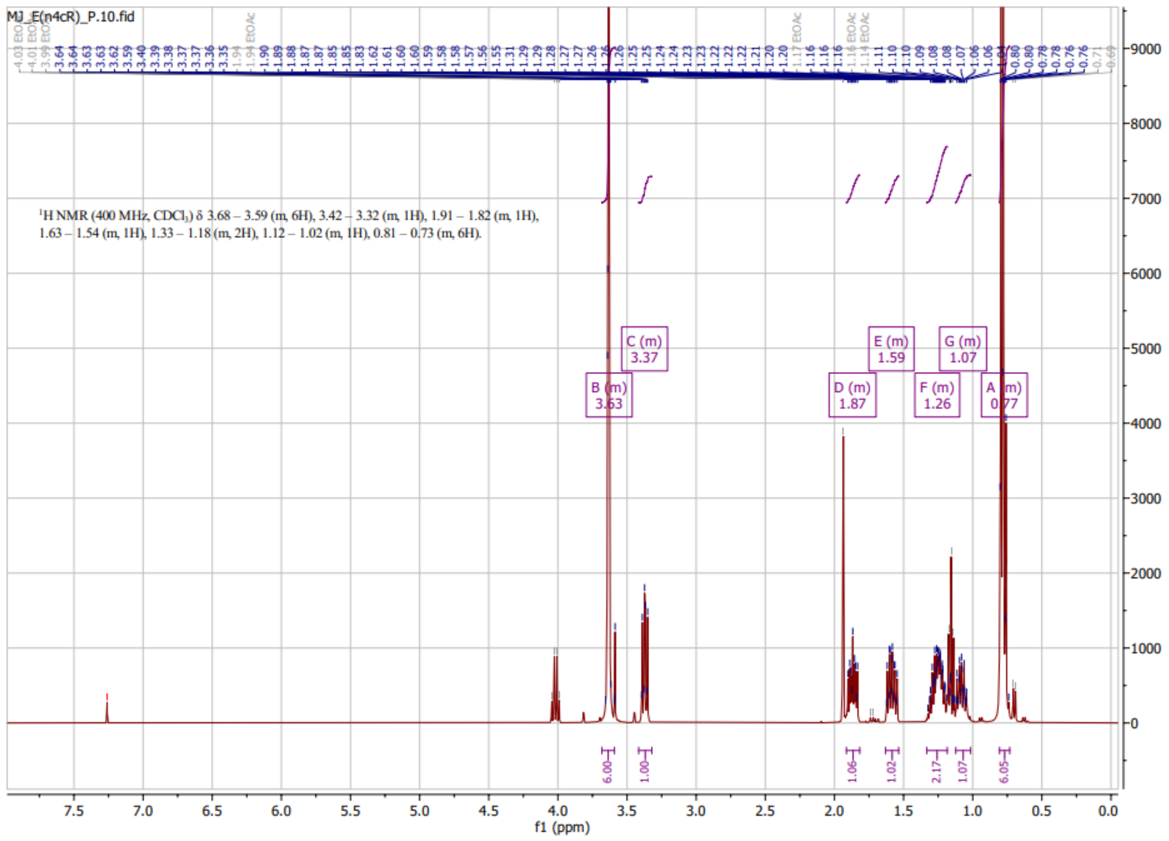

Figure S1: ^{1}H NMR spectrum of **1(S)** in $CDCl_3$.

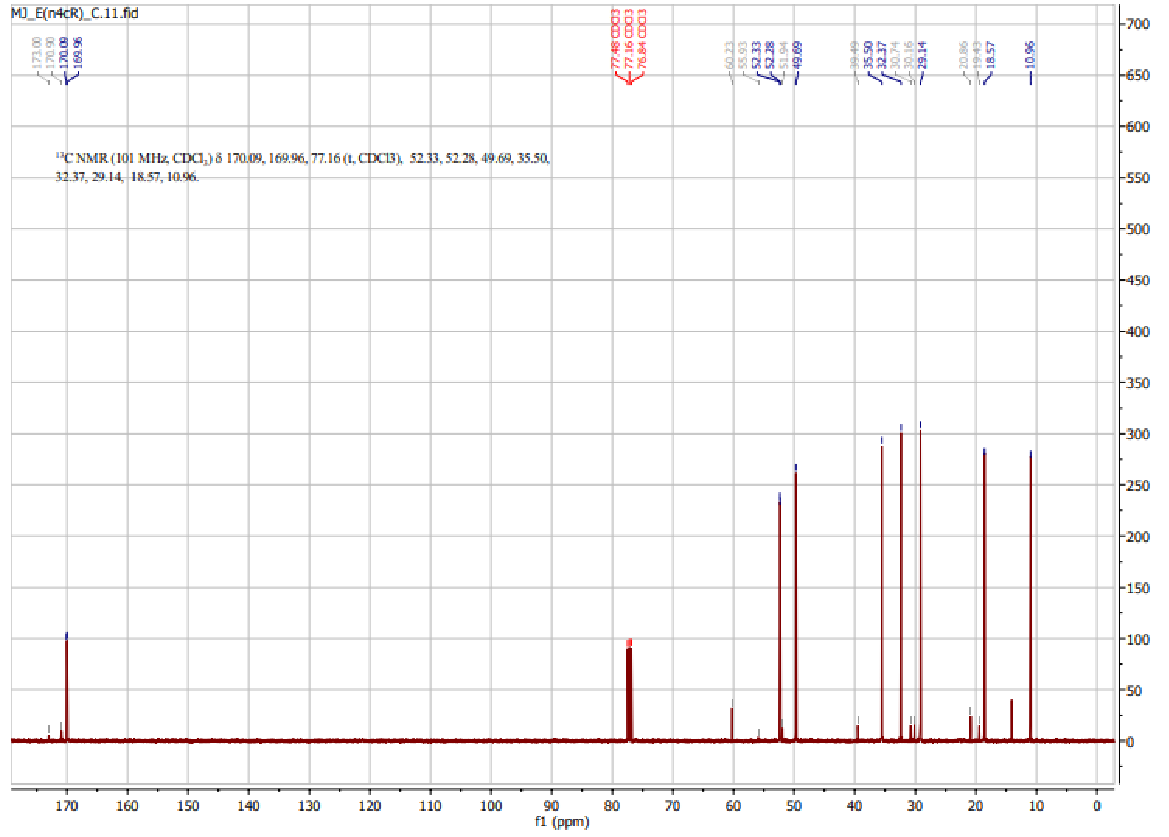

Figure S2: ^{13}C NMR spectrum of **1(S)** in $CDCl_3$.

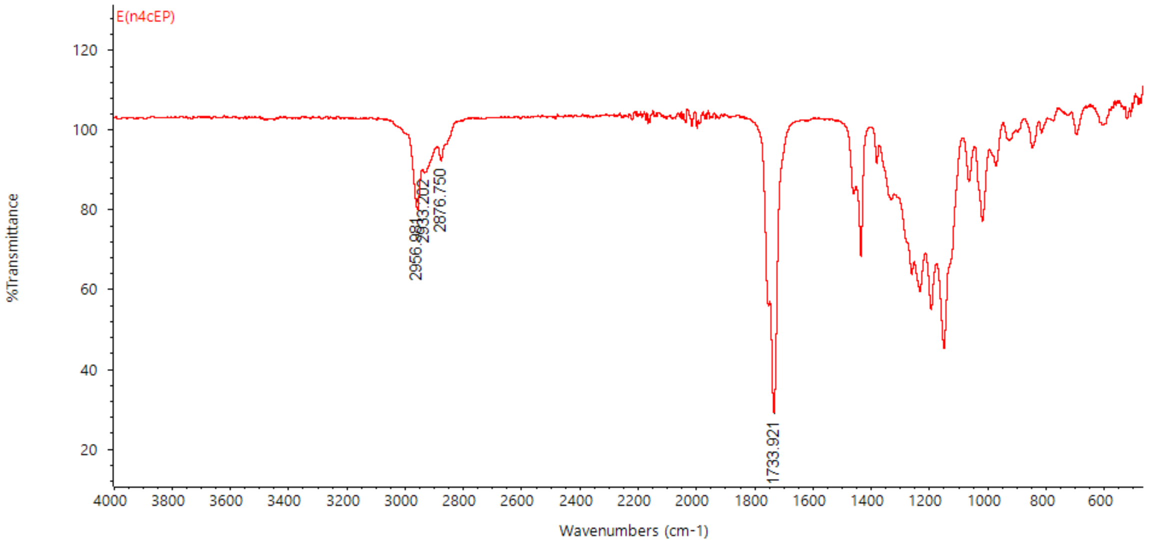

Figure S3: IR *s*pectrum of **1(S)**.

1(rac):

Yield: 37.6 %

^{1}H NMR (400 MHz, $CDCl_3$): δ ppm = 3.70 (6H, d, *J* 1.6 Hz, -$(OCH_3)_2$), 3.44 (1H dd, *J* 8.8 Hz, 6.6 Hz, -$(CO)_2$-CH-CH_2-), 1.99 – 1.89 (1H, m, -CH-CH(-H)-CH-), 1.71 – 1.59 (1H m, -CH-CH(-H)-CH-), 1.38 – 1.25 (2H, m, -CH-CH_2-CH_3), 1.21 – 1.09 (1H, m,-CH-CH-(CH_3)-CH_2-), 0.91 – 0.76 (6H, m, -CH_2-CH_3, -CH-CH_3).

^{13}C NMR (101 MHz, $CDCl_3$): δ ppm = 170.31, 170.17, 52.55, 52.50, 49.87, 49.85, 35.65, 35.63, 32.51, 32.49, 29.28, 29.26, 18.73, 18.71, 11.14, 11.12.

IR (ν_{max}/cm^{-1}): 2958 (sp^3 C-H stretch), 2933 (sp^3 C-H stretch), 2877 (sp^3 C-H stretch), 1733 (C=O stretch, ester).

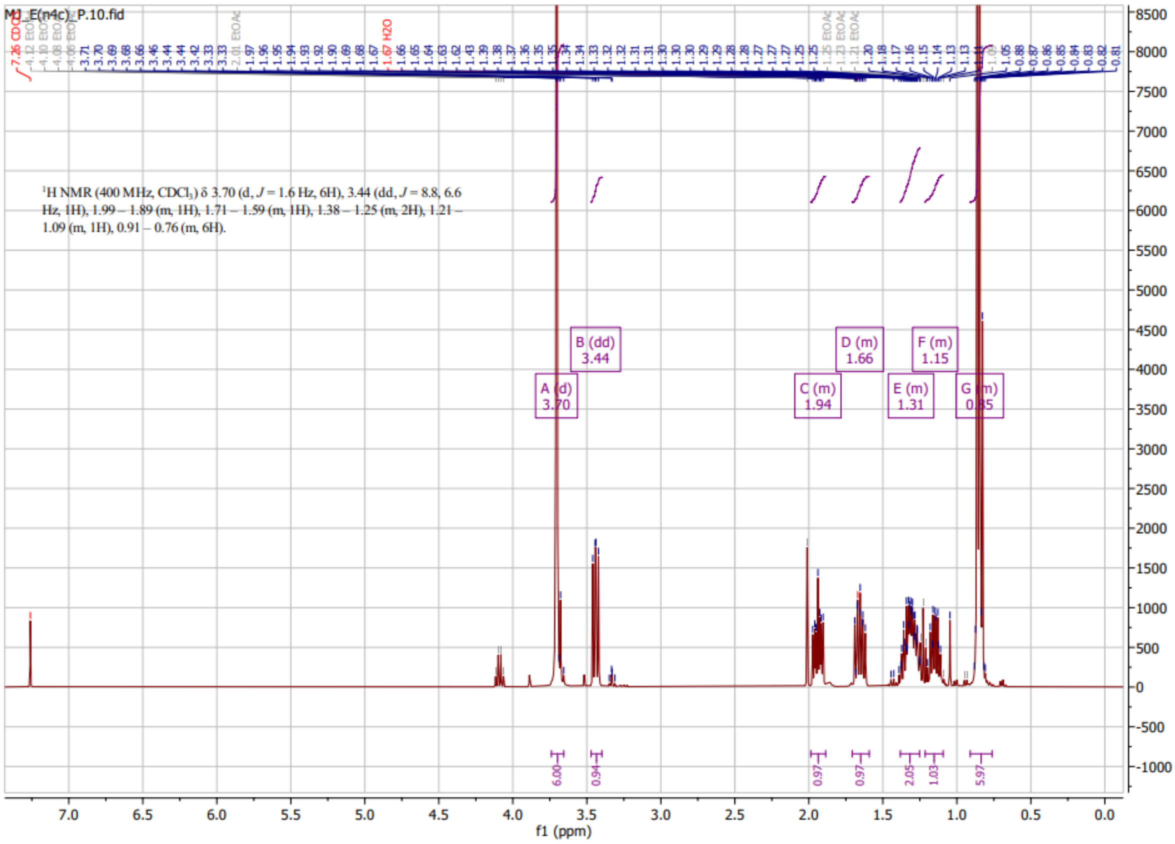

Figure S4: ¹H NMR spectrum of **1(rac)** in CDCl₃.

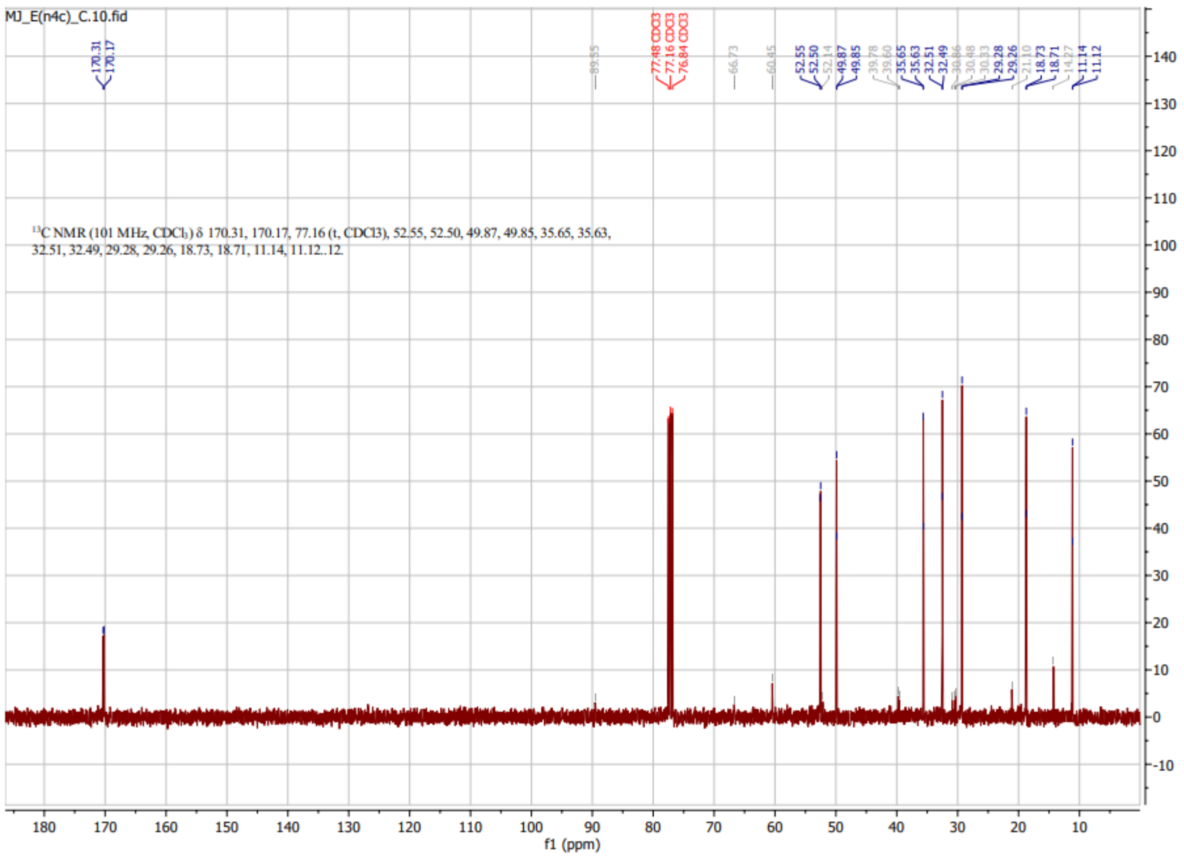

Figure S5: ^{13}C NMR spectrum of **1(rac)** in $CDCl_3$.

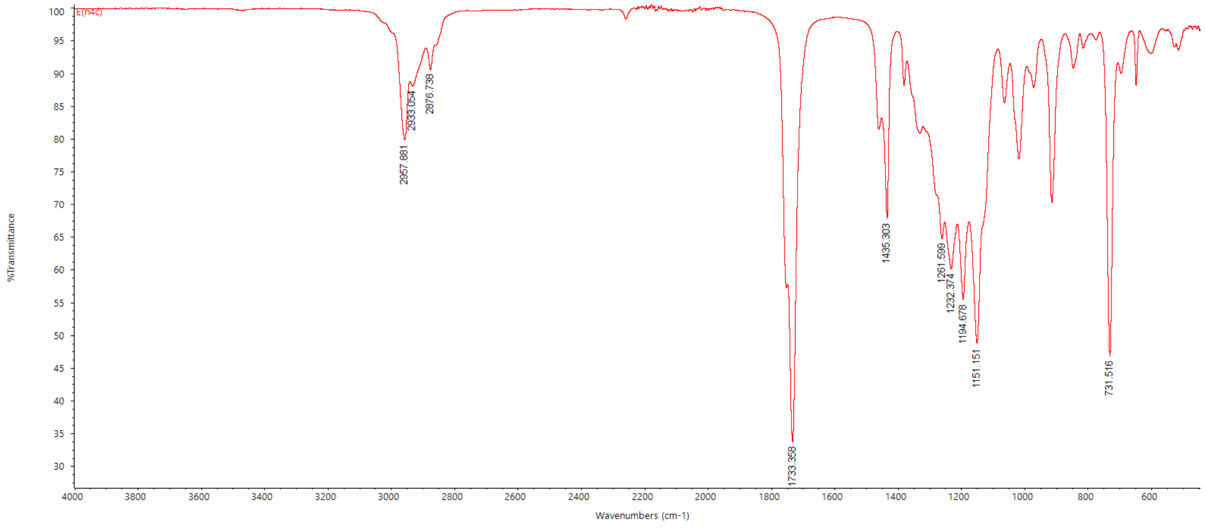

Figure S6: IR *s*pectrum of **1(rac)**.

2-[(2S)-2-methylbutyl]propane-1,3-diol (2(S)) /

2-[2-methylbutyl]propane-1,3-diol (2(rac))

Anhydrous THF (20 mL) was added to a 2-necked RBF under argon atmosphere. To the stirred solvent, $LiAlH_4$ solution (2 M, 4.1 eq) was added using a syringe. The solution was cooled to 0 ^oC using an ice bath, and the 2-alkyl-dimethylmalonate (1 eq) was added dropwise using a syringe. The reaction mixture was allowed to slowly warm to room temperature and stirred for 8h. The reaction mixture was then cooled down to 0 ^oC once again and acidified with HCl (2 N) followed by the addition of dilute acetic acid solution (1 M, 10 mL) to quench the reaction. The product was then extracted with Et_2O (3 x 10 mL), combined organic layers were washed with water (2 x 20 mL), brine (40 mL), dried over $MgSO_4$ and purified with flash chromatography (250 mL of 8:2, 100 mL of 1:1, 200 mL of 3:7, PE/EtOAc) to yield a colourless oil (Rf = 0.15 (1:1 PE/EtOAc)).

2(S):

Yield: 45.8 %

^{1}H NMR (400 MHz, $CDCl_3$): δ ppm = 3.85 – 3.74 (2H, m, -CH-$\underline{CH_2}$-O-), 3.68 – 3.55 (2H, m,
-CH-$\underline{CH_2}$-O-), 2.47 (1H, s,-OH), 2.15 (1H, s, -OH), 1.94 – 1.83 (1H, m, -CH_2-$\underline{CH}$-(CH_2-)$_2$), 1.43 – 1.29 (2H, m, -CH-$\underline{CH(-H)}$-CH-), 1.28 – 1.07 (2H, m, -CH-$\underline{CH(-H)}$-CH_3), 1.00 – 0.91 (1H, m,-CH_2-$\underline{CH}$(-CH_3)-CH_2-), 0.91 – 0.80 (6H, m,-CH-$\underline{CH_3}$; -CH_2-$\underline{CH_3}$).

^{13}C NMR (101 MHz, $CDCl_3$): δ ppm = 66.43, 65.62, 39.44, 34.78, 31.81, 29.78, 19.34, 11.30.

IR (v_{max}/cm^{-1}): 3600-3000 (broad, -OH stretch), 2958 (sp^3 C-H stretch), 2917 (sp^3 C-H stretch), 2875 (sp^3 C-H stretch).

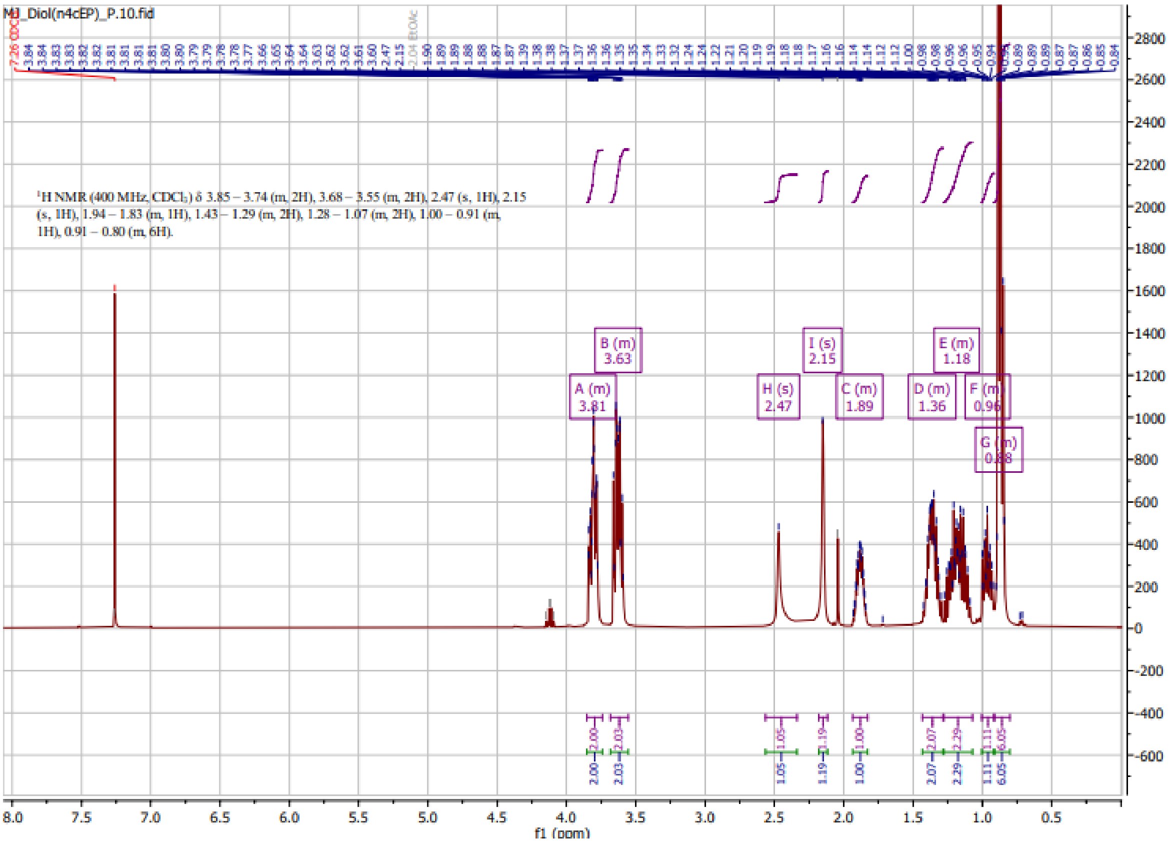

Figure S7: ^{1}H NMR spectrum of **2(S)** in $CDCl_3$.

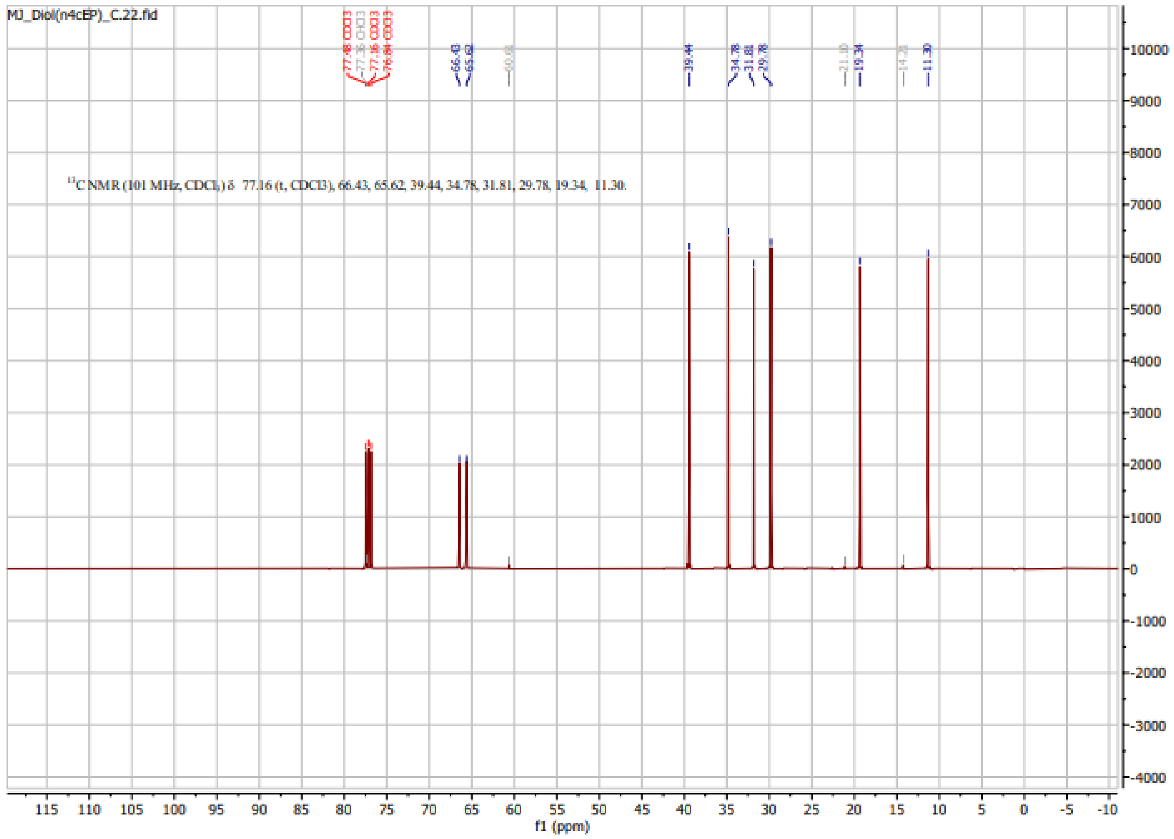

Figure S8: ^{13}C NMR spectrum of **2(S)** in $CDCl_3$.

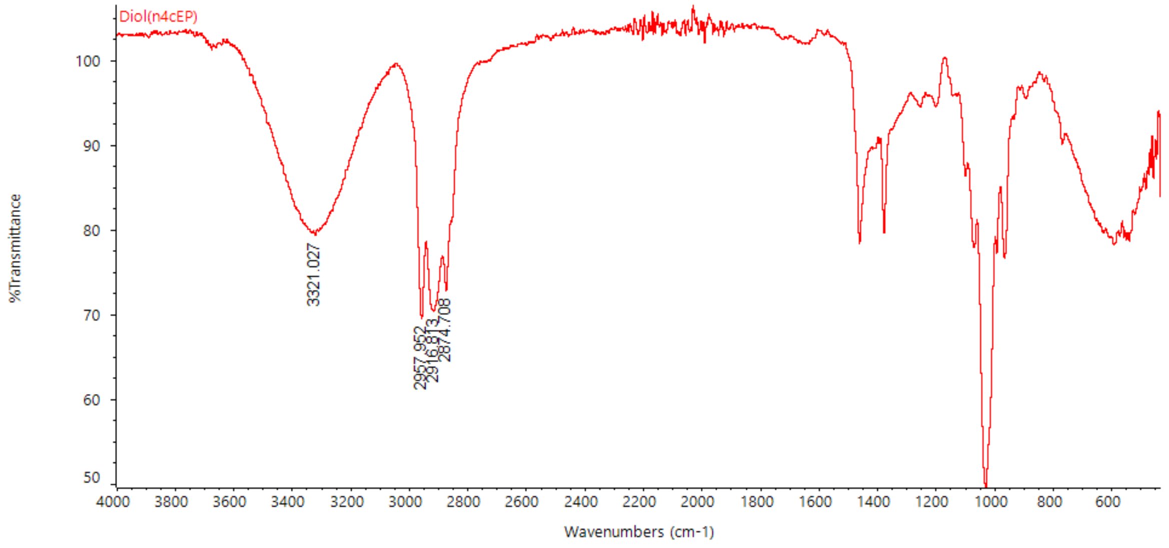

Figure S9: IR *s*pectrum of **2(S)**.

2(rac):

Yield: 31.3 %

^{1}H NMR (400 MHz, $CDCl_3$): δ ppm = 3.83 – 3.72 (2H, m, -CH-$\underline{CH_2}$-O-), 3.65 – 3.52 (2H, m, -CH-$\underline{CH_2}$-O-), 2.91 (2H, s, $(\text{-OH})_2$), 1.91 – 1.81 (1H, m, ,-CH_2-$\underline{CH}$-(CH_2-O-)$_2$), 1.43 – 1.27 (2H, m, -CH-$\underline{CH_2}$-CH(-CH_3)-), 1.22 – 1.07 (2H, m, -CH(-CH_3)-$\underline{CH_2}$-CH_3), 0.98 – 0.90 (1H, m, -CH_2-$\underline{CH}$(-CH_3)-CH_2-), 0.89 – 0.80 (6H, m, -CH-$\underline{CH_3}$; -CH_2-$\underline{CH_3}$).

^{13}C NMR (101 MHz, $CDCl_3$): δ ppm = 67.44, 66.63, 39.48, 34.78, 31.91, 29.84, 19.42, 11.37.

IR (ν_{max}/cm^{-1}): 3600-3000 (broad, -OH stretch), 2958 (sp^3 C-H stretch), 2920- (sp^3 C-H stretch), 2874 (sp^3 C-H stretch).

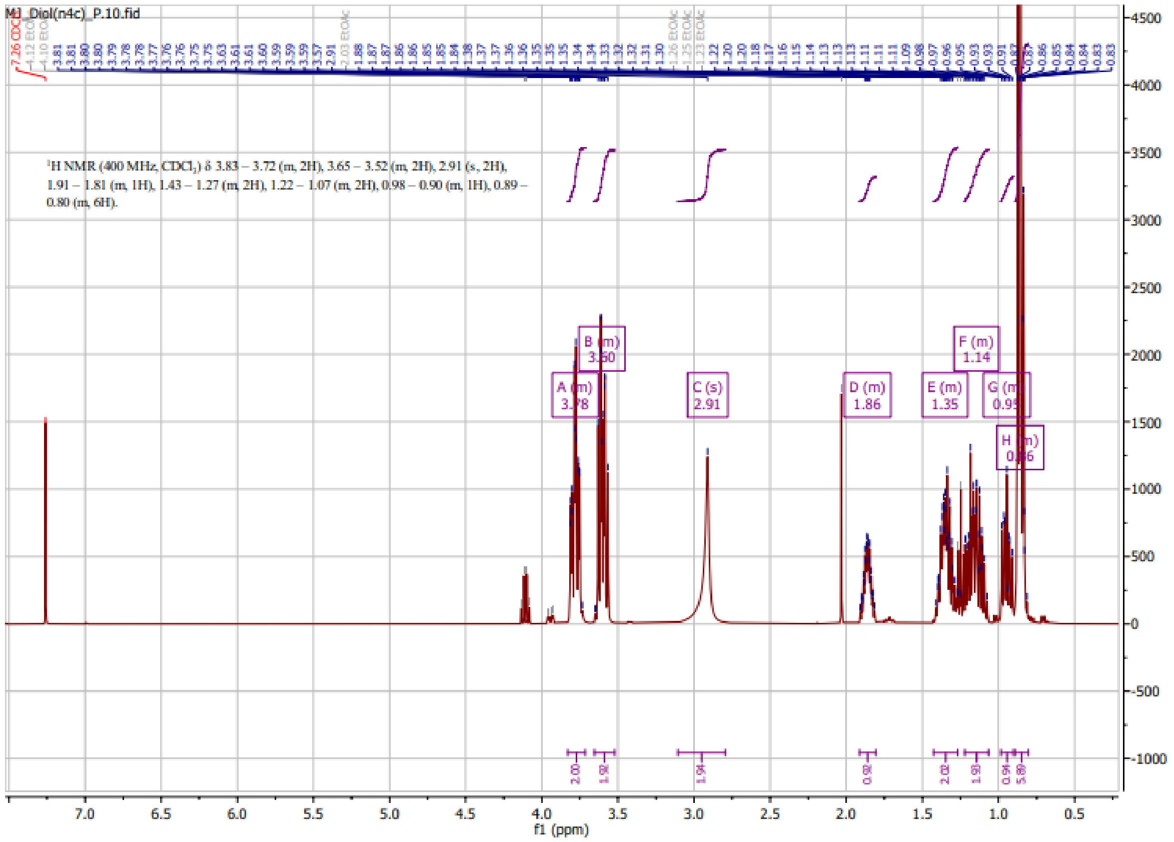

Figure S10: ^{1}H NMR spectrum of **2(rac)** in $CDCl_3$.

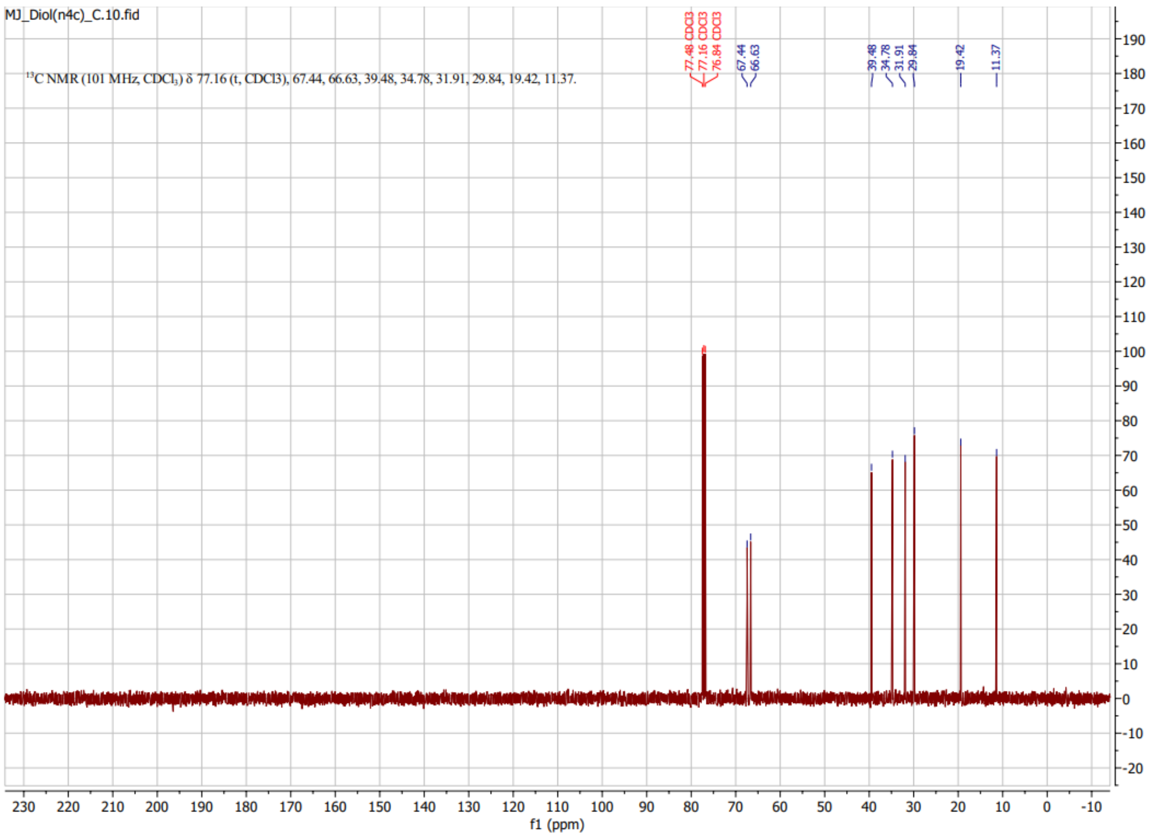

Figure S11: ^{13}C NMR spectrum of **2(rac)** in $CDCl_3$.

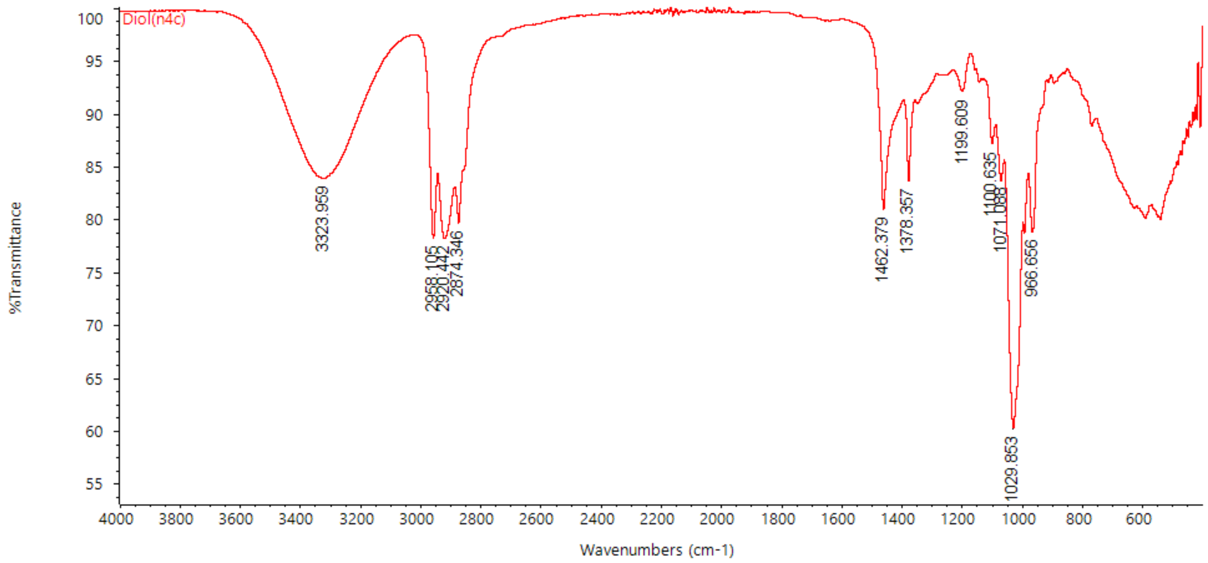

Figure S12: IR spectrum of **2(rac)**.

2-(3,5-difluorophenyl)-5-[(2S)-2-methylbutyl]-1,3-dioxane (3(S)) /

2-(3,5-difluorophenyl)-5-[2-methylbutyl]-1,3-dioxane (3(rac))[1]

In a RBF, equipped with magnetic stirrer, 2-alkylpropane-1,3-diol (1.1 eq) and 3,5-difluorobenzaldehyde (1 eq) are added into 1M HCl saturated aqueous $CaCl_2$ solution. The stirring is then turned on and the reaction is monitored with TLC (96:4, PE/EtOAc) until a full conversion is seen. Then, the reaction mixture is diluted with H_2O (50 mL) and crude product is extracted with DCM (3 x 50 mL). Collected organic extracts are combined and washed with brine (80 mL), dried with anhydrous $MgSO_4$. ^{1}H NMR ($CDCl_3$) is then performed and, in case of some unreacted aldehyde remaining, the crude is purified using Biotage® Selekt (PE/EtOAc gradient) to yield a colourless oil. (Rf (*trans*- isomer)= 0.55 (96/4 PE/EtOAc))

3(S):

Yield: 95.5 %

***^{1}H NMR** (400 MHz, $CDCl_3$): δ ppm = 7.07 – 6.97 (2H, m, Ar-H), 6.76 (1H, tt, *J* 8.9 Hz, 2.4 Hz, Ar-H), 5.36 (1H, s, Ar-CH-), 4.20 (2H, dddd, *J* 15.7 Hz, 11.2 Hz, 4.6 Hz, 2.3 Hz, -CH-CH_2-O-), 3.50 (2H, td, *J* 11.1 Hz, 7.2 Hz, -CH-CH_2-O-), 2.27 – 2.14 (1H, m, -CH(-H)-CH-(CH_2-O-)$_2$), 1.39 – 1.30 (2H, m, -CH-CH_2-CH(-CH_3)-), 1.20 – 1.12 (1H, m, -CH(-CH_3)-CH(-H)-CH_2-), 1.10 – 1.02 (1H, m, -CH(-CH_3)-CH(-H)-CH_2-), 0.90 – 0.84 (7H, m, -CH_2-CH_3, -CH-CH(-CH_3)-CH_2-).

^{13}C NMR (101 MHz, $CDCl_3$): δ ppm = 162.98 (dd, $J_{C\text{-}F\text{-}i}$ = 248.1 Hz, $J_{C\text{-}F\text{-}m}$ = 12.4 Hz), 142.27 (t, $J_{C\text{-}F\text{-}o}$ = 9.2 Hz), 109.96 – 108.87 (m), 104.09 (t, $J_{C\text{-}F\text{-}m}$ = 25.4 Hz), 99.75 (t, $J_{C\text{-}F\text{-}l.r.c.}$ = 2.6 Hz), 72.98, 72.72, 35.08, 31.91, 31.30, 29.77, 19.37, 11.28. (*i* – ipso coupling; *o* – ortho coupling; *m* – meta coupling; l.r.c. – long range coupling)

IR (ν_{max}/cm^{-1}): 3082 (sp^2 hybridised C-H stretching), 2962 (sp^3 hybridised C-H stretching), 2926 (sp^3 hybridised C-H stretching), 2876 (sp^3 hybridised C-H stretching), 1599 (-C=C- stretching, aromatic).

*Only *trans*-isomer peaks elucidated. *Cis*-/*trans*- ratio: 0.17/1

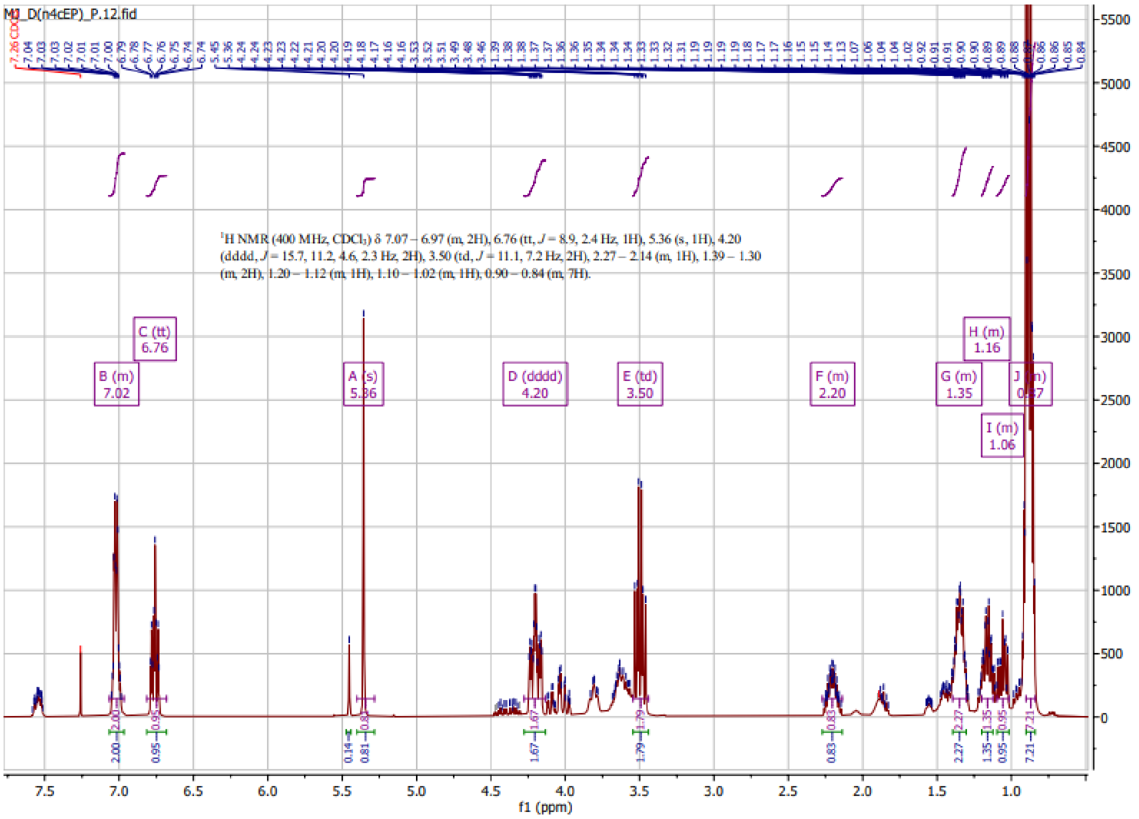

Figure S13: ^{1}H NMR spectrum of **3(S)** in $CDCl_3$.

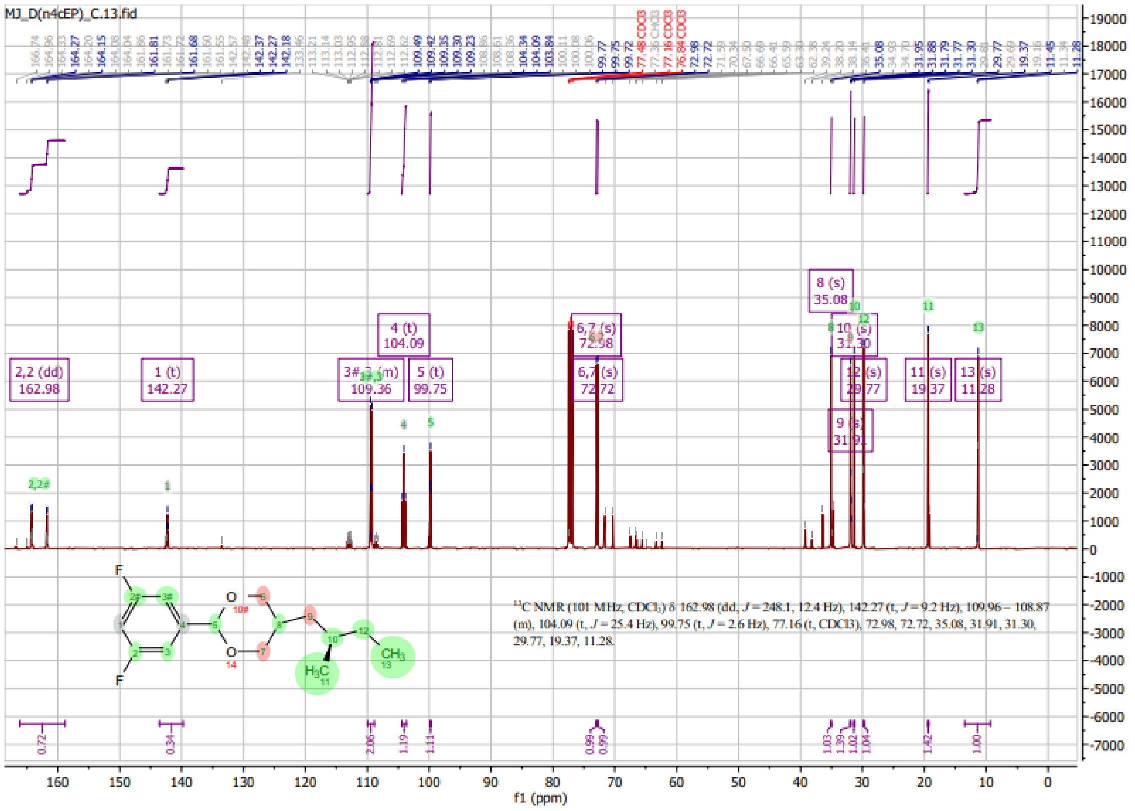

Figure S14: ^{13}C NMR spectrum of **3(S)** in $CDCl_3$.

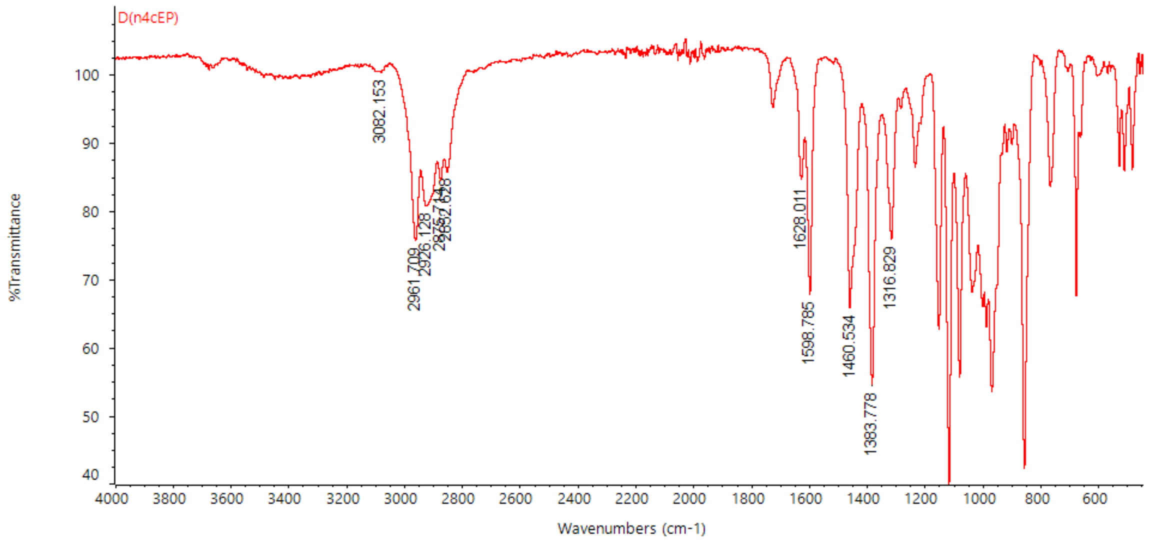

Figure S15: IR *s*pectrum of **3(S)**.

3(rac):

Yield: 78.6 %

***^{1}H NMR** (400 MHz, $CDCl_3$): δ ppm = 7.07 – 6.98 (2H, m, Ar-H), 6.77 (1H, tt, *J* 8.9 Hz, 2.5 Hz, Ar-H), 5.36 (1H, s, Ar-CH-), 4.21 (2H, dddd, *J* 15.8 Hz, 11.2 Hz, 4.6 Hz, 2.3 Hz, -CH-CH_2-O-), 3.49 (2H, td, *J* 11.1 Hz, 7.3 Hz, -CH-CH_2-O-), 2.28 – 2.14 (1H, m, -CH_2-CH-(CH_2-O-)$_2$), 1.41 – 1.29 (2H, m, -CH-CH_2-CH(-CH_3)-), 1.23 – 1.11 (1H, m, -CH(-CH_3)- CH(-H)-CH_2-), 1.11 – 1.00 (1H, m, -CH(-CH_3)-CH(-H)-CH_2-), 0.91 – 0.86 (7H, m, -CH_2-CH_3, -CH-CH(-CH_3)-CH_2-).

^{13}C NMR (101 MHz, $CDCl_3$): δ ppm = 162.96 (dd, $J_{C\text{-}F\text{-}i}$ = 248.1 Hz, $J_{C\text{-}F\text{-}m}$ = 12.4 Hz), 142.37 (t, $J_{C\text{-}F\text{-}o}$ = 9.2 Hz), 109.75 – 108.89 (m), 104.00 (t, $J_{C\text{-}F\text{-}m}$ = 25.3 Hz), 99.69 (t, $J_{C\text{-}F\text{-}l.r.c.}$ = 2.6 Hz), 72.93, 72.68, 35.06, 31.94, 31.29, 29.76, 19.33, 11.24. (*i* – ipso coupling; *o* – ortho coupling; *m* – meta coupling; l.r.c. – long range coupling)

IR (ν_{max}/cm^{-1}): 3092 (sp^2 hybridised C-H stretching), 2962 (sp^3 hybridised C-H stretching), 2924 (sp^3 hybridised C-H stretching), 2849 (sp^3 hybridised C-H stretching), 2008 (C-H bending, aromatic overtone), 1599 (-C=C- stretching, aromatic).

*Only *trans*-isomer peaks elucidated. *Cis-/trans-* ratio: 0.14/1

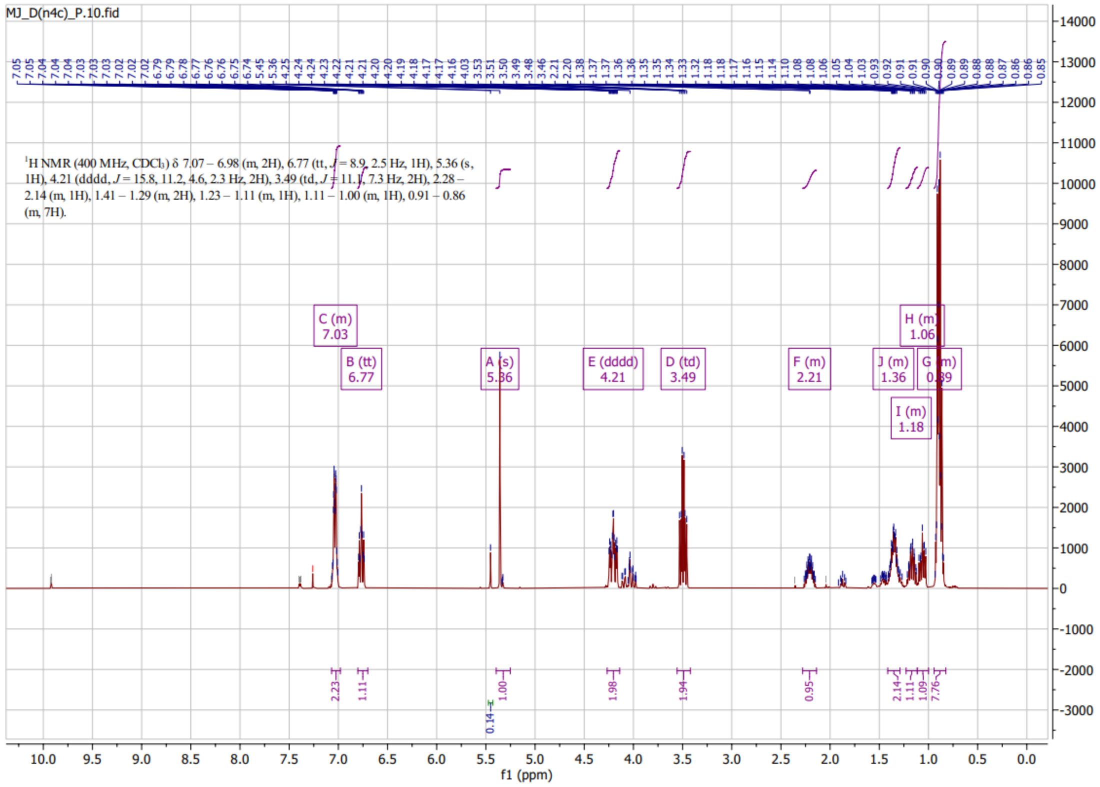

Figure S16: ^{1}H NMR spectrum of **3(rac)** in CDCl$_3$.

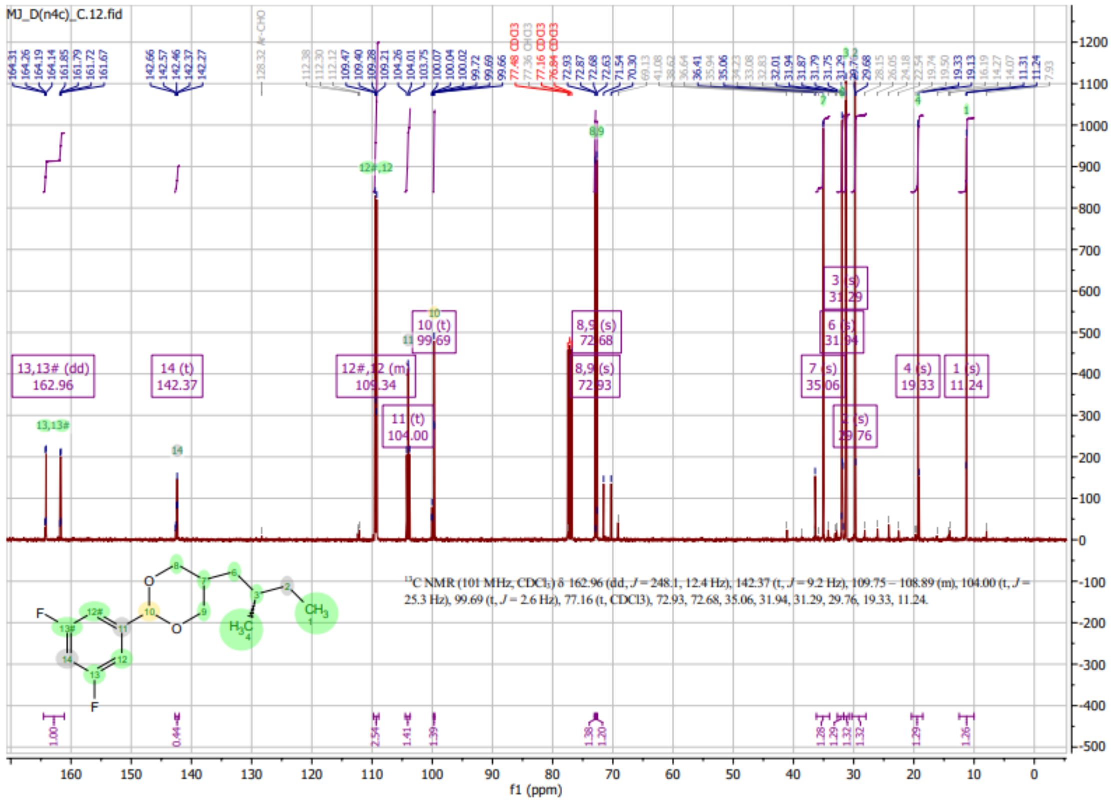

Figure S17: ^{13}C NMR spectrum of **3(rac)** in CDCl$_3$.

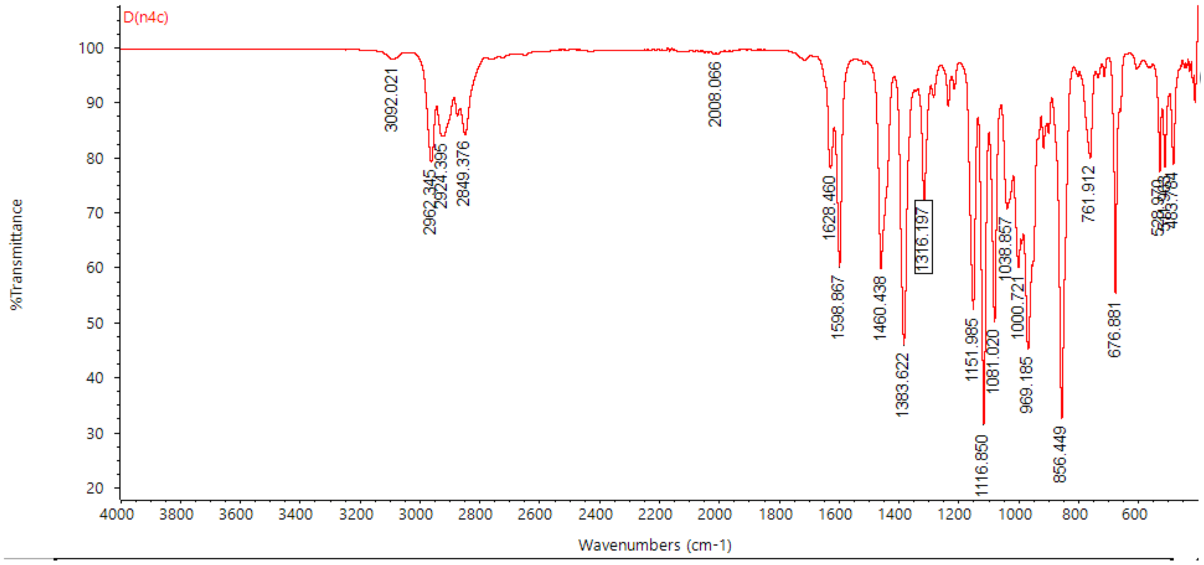

Figure S18: IR spectrum of **3(rac)**.

2,6-difluoro-4-[5-[(2S)-2-methylbutyl]-1,3-dioxan-2-yl]benzoic acid (4(S)) / 2,6-difluoro-4-[5-[2-methylbutyl]-1,3-dioxan-2-yl]benzoic acid (4(rac))[2]

Anhydrous 2-(3,5-difluorophenyl)-5-alkyl-1,3-dioxane (1 eq) was added to a RBF under an argon atmosphere. Anhydrous THF (25 mL) was added, the reaction vessel was cooled down to -78 ^{o}C using acetone/dry ice bath. Then, *n*-BuLi (2.5 M, 1.1 eq) was added, and the reaction mixture was stirred under inert environment for at least 1 h. An excess of solid CO_2 was then added directly into the reaction vessel which was left to slowly heat up to room temperature. The reaction mixture was then acidified with HCl (1 M) to a pH=4, THF was removed *in vacuo.* The concentrated crude was diluted with Et_2O (30 mL) and washed with H_2O (2 x 20 mL). Then, diisopropylamine (1N) was added to form a water-soluble salt with the reaction product, and two additional washes with H_2O were carried out (2 x 20 mL). The aqueous layers were combined and acidified until a white precipitate formation was observed. The crystals were then filtered off under vacuum, washed with H_2O (15 mL) and, to ensure the highest possible purity whilst minimising product loss, recrystallised in hexane/toluene mixture (1/4) to yield large white needle-like crystals.

4(S):

Yield: 82.5 %

m.p. = 113.4 °C

^{1}H NMR (400 MHz, $CDCl_3$): δ ppm = 7.18 – 7.09 (2H, m, Ar-H), 5.37 (1H, s, Ar-CH-), 4.22 (2H, dddd, *J* 15.6 Hz, 11.4 Hz, 4.6 Hz, 2.2 Hz, -CH-$\underline{CH_2}$-O-), 3.51 (2H, td, *J* 11.1 Hz, 7.2 Hz, -CH-$\underline{CH_2}$-O-), 2.26 – 2.15 (1H, m, -CH_2-$\underline{CH}$-(CH_2-O-)$_2$), 1.40 – 1.30 (2H, m, -CH-$\underline{CH2}$-CH(-CH_3)-), 1.21 – 1.13 (1H, m, -CH(-CH_3)-$\underline{CH(-H)}$-CH_3), 1.10 – 1.03 (1H, m, -CH(-CH_3)-$\underline{CH(-H)}$-CH_3), (7H, m, -CH_2-$\underline{CH_3}$, -CH_2-$\underline{CH(-CH_3)}$-CH(-H)-).

^{13}C NMR (101 MHz, $CDCl_3$): δ ppm = 166.14 (t, $J_{C\text{-}F\text{-}l.r.c.}$ = 1.8 Hz), 161.23 (dd, $J_{C\text{-}F\text{-}i}$ = 259.4 Hz, $J_{C\text{-}F\text{-}m}$ = 5.7 Hz), 145.39 (t, $J_{C\text{-}F\text{-}o}$ = 10.1 Hz), 110.44 – 109.99 (m), 109.28 (t, $J_{C\text{-}F\text{-}m}$ = 16.4 Hz), 98.85 (t, $J_{C\text{-}F\text{-}l.r.c.}$ = 2.3 Hz), 72.89, 72.64, 34.94, 31.82, 31.20, 29.67, 19.28, 11.19. (*i* – ipso coupling; *o* – ortho coupling; *m* – meta coupling; l.r.c. – long range coupling)

IR (ν_{max}/cm^{-1}): 3400-2500 (OH stretch, carboxylic acid), 3080 (sp^2 hybridised -C-H stretch), 2967 (sp^3 hybridised -C-H stretch), 2899 (sp^3 hybridised -C-H stretch) 2001 (aromatic overtone), 1699 (C=O stretching, carboxylic acid), 1636 (C=C stretch, aromatic).

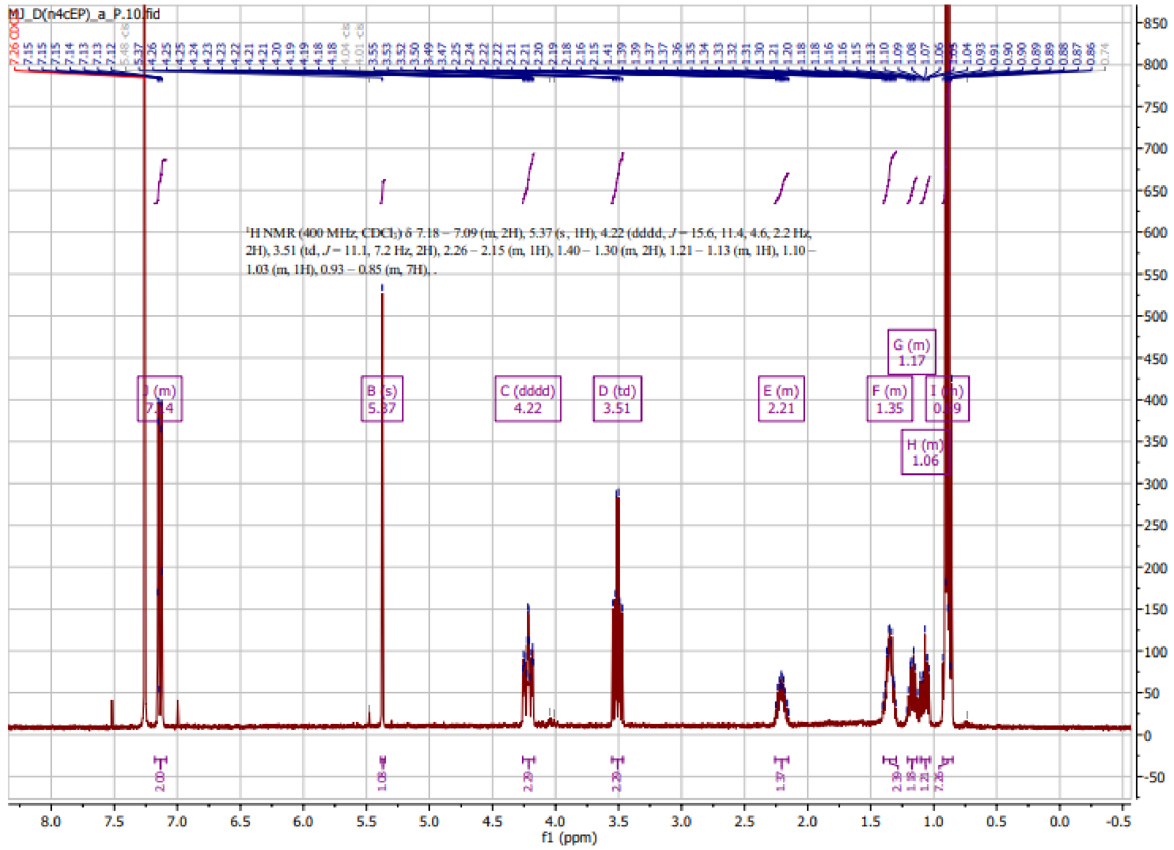

Figure S19: ^{1}H NMR spectrum of **4(S)** in $CDCl_3$.

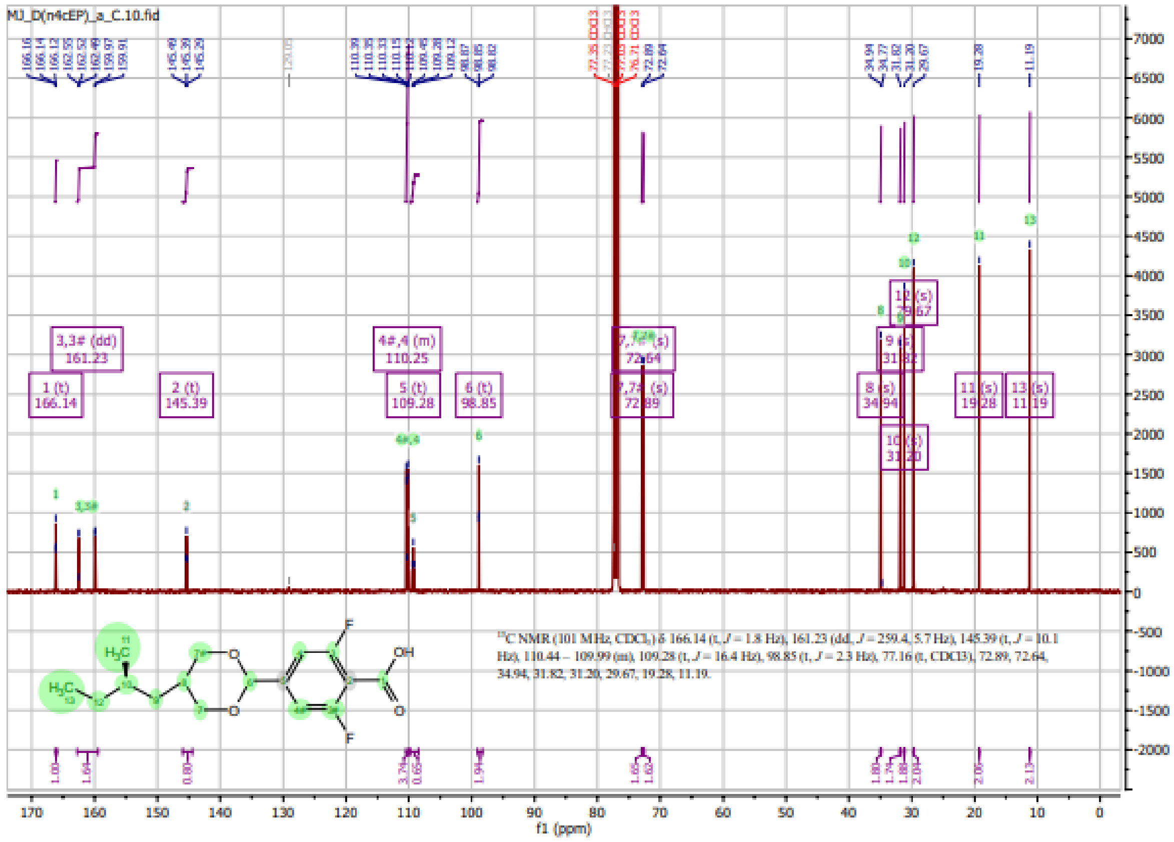

Figure S20: ^{13}C NMR spectrum of **4(S)** in $CDCl_3$.

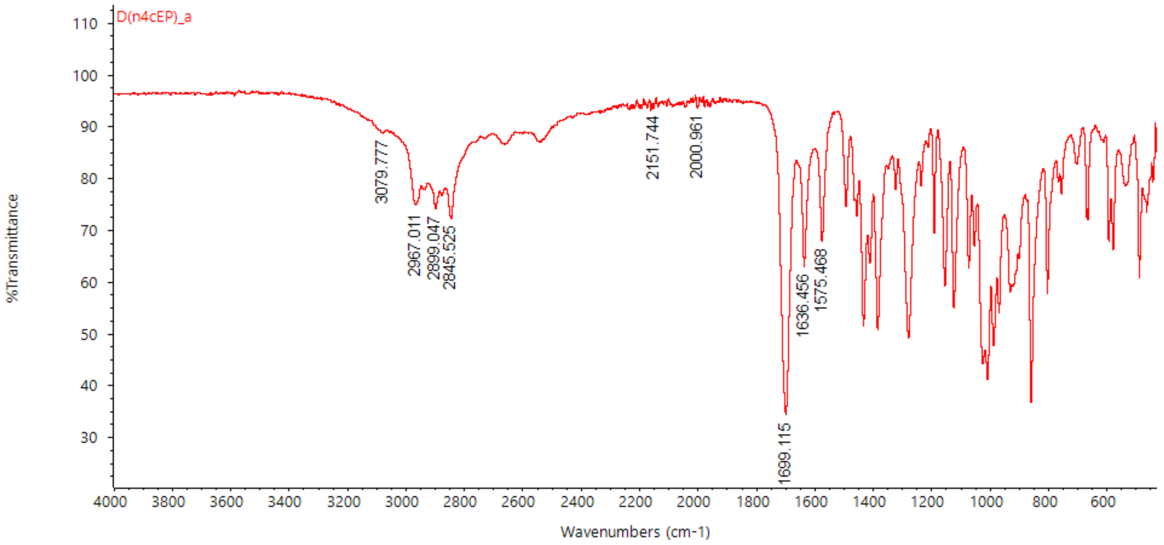

Figure S21: IR *s*pectrum of **4(S)**.

4(rac):

Yield: 78.3 %

m.p. = 111.7 °C

^{1}H NMR (400 MHz, $CDCl_3$): δ ppm = 10.97 (1H, s, -COOH), 7.20 – 7.04 (2H, m, Ar-H), 5.38 (1H, s, Ar-CH-), 4.22 (2H, dddd, *J* 15.6 Hz, 11.2 Hz, 4.7 Hz, 2.3 Hz, -CH-CH_2-O-), 3.51 (2H, td, *J* 11.1 Hz, 7.1 Hz, -CH-CH_2-O-), 2.30 – 2.12 (1H, m, -CH_2-CH-(CH_2-O-)$_2$), 1.41 – 1.28 (2H, m, -CH-CH2-CH(-CH_3)-), 1.23 – 1.12 (1H, m, -CH(-CH_3)-CH(-H)-CH_3), 1.10 – 0.99 (1H, m, -CH(-CH_3)-CH(-H)-CH_3), 0.96 – 0.75 (7H, m, -CH_2-CH_3, -CH_2-CH(-CH_3)-CH(-H)-).

^{13}C NMR (101 MHz, $CDCl_3$): δ ppm = 166.63, 161.36 (dd, $J_{C\text{-}F\text{-}i}$ = 259.4 Hz, $J_{C\text{-}F\text{-}m}$ = 5.7 Hz), 145.53 (t, $J_{C\text{-}F\text{-}o}$ = 10.0 Hz), 110.77 – 110.02 (m), 109.43 (t, $J_{C\text{-}F\text{-}m}$ = 16.3 Hz), 98.98 (t, $J_{C\text{-}F\text{-}l.r.c.}$ = 2.3 Hz), 73.01, 72.77, 35.06, 31.95, 31.33, 29.79, 19.40, 11.31.
(*i* – ipso coupling; *o* – ortho coupling; *m* – meta coupling; l.r.c. – long range coupling)

IR (ν_{max}/cm^{-1}): 3400-2500 (OH stretch, carboxylic acid), 3087 (sp^2 hybridised -C-H stretch), 2960 (sp^3 hybridised -C-H stretch), 2926 (sp^3 hybridised -C-H stretch) 1988 (aromatic overtone), 1702 (C=O stretching, carboxylic acid), 1637 (C=C stretch, aromatic).

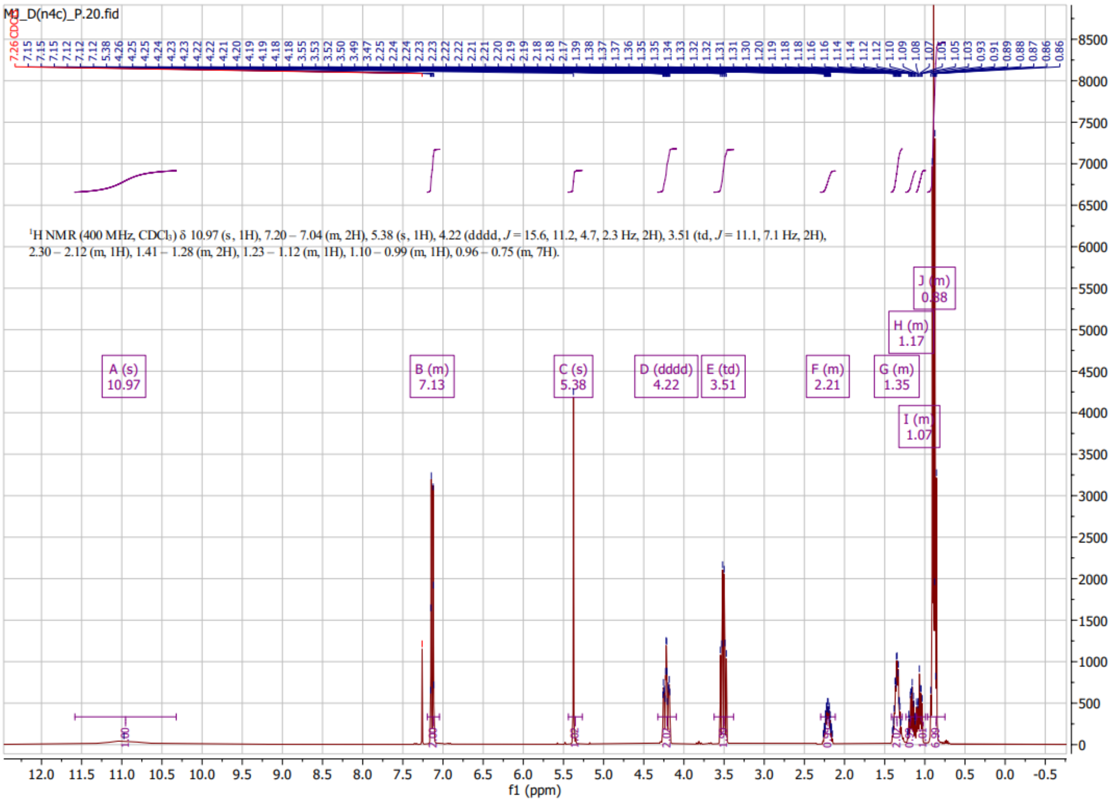

Figure S22: ^{1}H NMR spectrum of **4(S)** in $CDCl_3$.

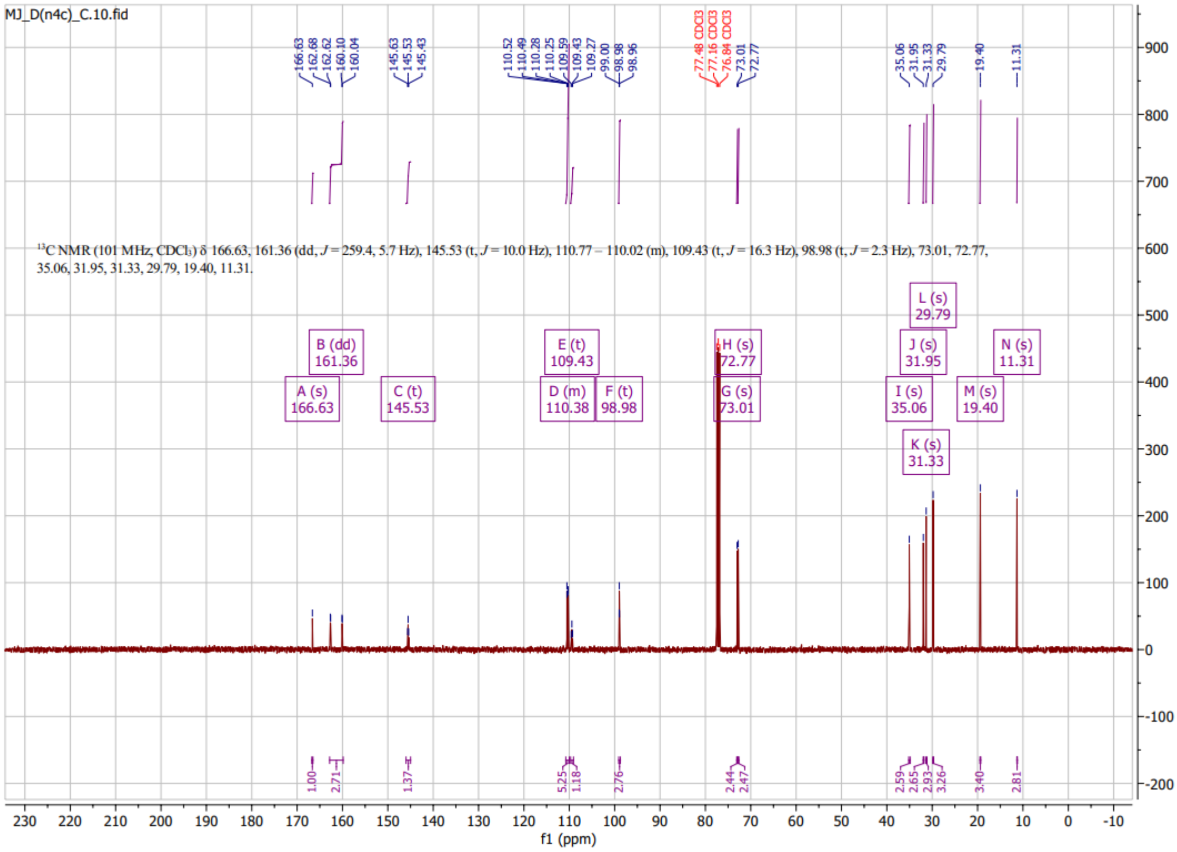

Figure S23: ^{13}C NMR spectrum of **4(S)** in $CDCl_3$.

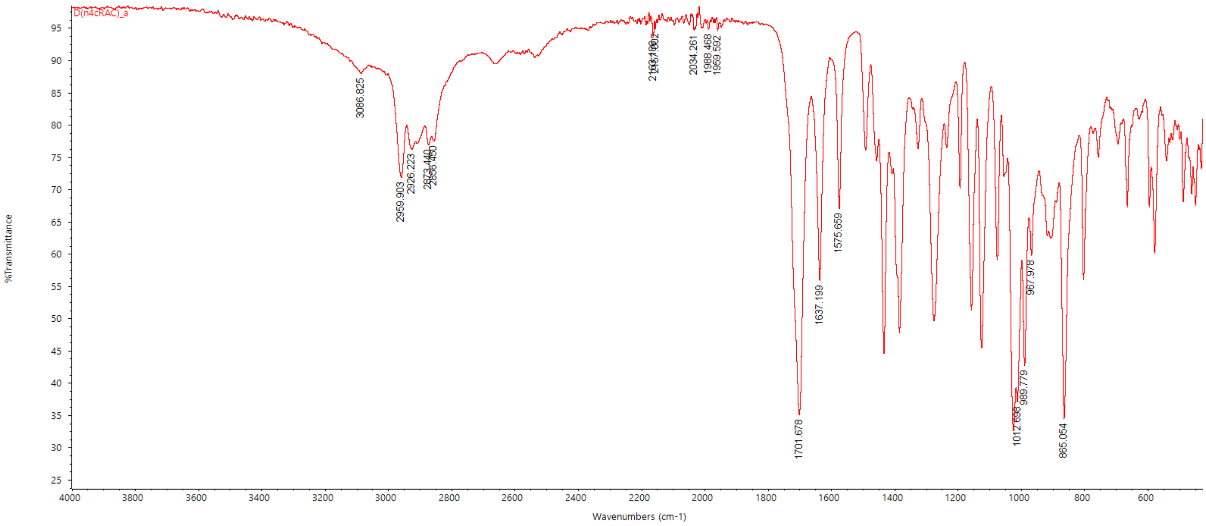

Figure S24: IR *s*pectrum of **4(S)**.

2-(4-(difluoro(3,4,5-trifluorophenoxy)methyl)-3,5-difluorophenyl)-4,4,5,5-tetramethyl-1,3,2-dioxaborolane (5)[3]

5-Bromo-2-(difluoro(3,4,5-trifluorophenoxy)methyl)-1,3-difluorobenzene (4.95 g, 12.7 mmol, 1.00 eq.), bis(pinacolato)diboron (3.23 g, 12.7 mmol, 1.00 eq.) and dry potassium acetate (3.75 g, 38.2 mmol, 3.00 eq.) were suspended in a 1:1 mixture of dry toluene and dry 1,4-dioxane (200 mL) and sparged with argon for 1 hour. After adding the Pd-catalyst (1,1'-Bis(diphenylphosphino)ferrocene]palladium(II) dichloride, 278 mg, 381 µmol, 0.03 eq.) the mixture was stirred at 100°C for 20 h. The reaction mixture was allowed to cool down to rt and 1 M HCl was added to pH 4. The phases were separated, and the aqueous phase was extracted with toluene (3 x 50 mL). The combined organic extracts were washed with water (3 x 50 mL), while brine was added to enhance the separation. The combined organic extracts were dried over $MgSO_4$, and the solvent was removed under reduced pressure. The crude product was purified by column chromatography (DCM : *n*-hexane 1:1, Rf = 0.67) and recrystallisation in ethanol.

Yield: 3.81 g (75 %), white crystalline powder.

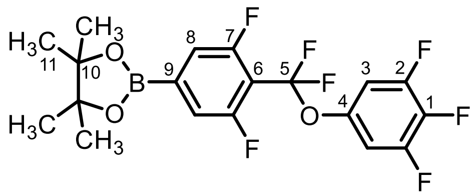

^{1}H NMR (400 MHz, $CDCl_3$): δ = 7.38 (d, $^3J_{HF}$ = 9.60 Hz, 2H, *H*-8), 6.96 (dd, $^3J_{HF}$ = 7.79 Hz, $^4J_{HF}$ = 6.00 Hz, 2H, *H*-3), 1.35 (s, 12H, *H*-11) ppm. **^{19}F NMR** (376 MHz, $CDCl_3$): δ = −61.8 (t, $^4J_{FF}$ = 27.1 Hz, 2F, *F*-5), −111.7 (td, $^4J_{FF}$ = 27.1 Hz, $^3J_{HF}$ = 9.84 Hz, 2F, *F*-7), −132.5 (dd, $^3J_{FF}$ = 20.9 Hz, $^3J_{HF}$ = 7.91 Hz, 2F, *F*-2), −163.5 (tt, $^3J_{FF}$ = 20.7 Hz, $^4J_{HF}$ = 5.62 Hz, 1F, *F*-1) ppm. **^{13}C NMR** (100 MHz, $CDCl_3$): δ = 159.6 (dt, $^1J_{CF}$ = 259 Hz, $^4J_{CF}$ = 2.20 Hz, *C*-7), 151.0 (app. dq, $^1J_{CF}$ = 251 Hz, $^2J_{CF}$ = 5.13 Hz, *C*-2), 144.7 (tt, $^2J_{CF}$ = 17.6 Hz, $^2J_{CF}$ = 2.20 Hz, *C*-6), 138.5 (dt, $^1J_{CF}$ = 250 Hz, $^2J_{CF}$ = 15.2 Hz, *C*-1), 135.8 (br, *C*-9), 120.3 (t, $^1J_{CF}$ = 266 Hz, *C*-5), 118.1 (m, *C*-3 or *C*-8), 111.7 (m, *C*-4), 107.5 (m, *C*-3 or *C*-8). 84.9 (s, *C*-10), 24.9 (s, *C*-11) ppm.
IR (ν_{max}/cm^{-1}): 3117 (=C−H), 2981 (−C−H), 1632 (C=C), 1043 (C−F) cm^{-1}.

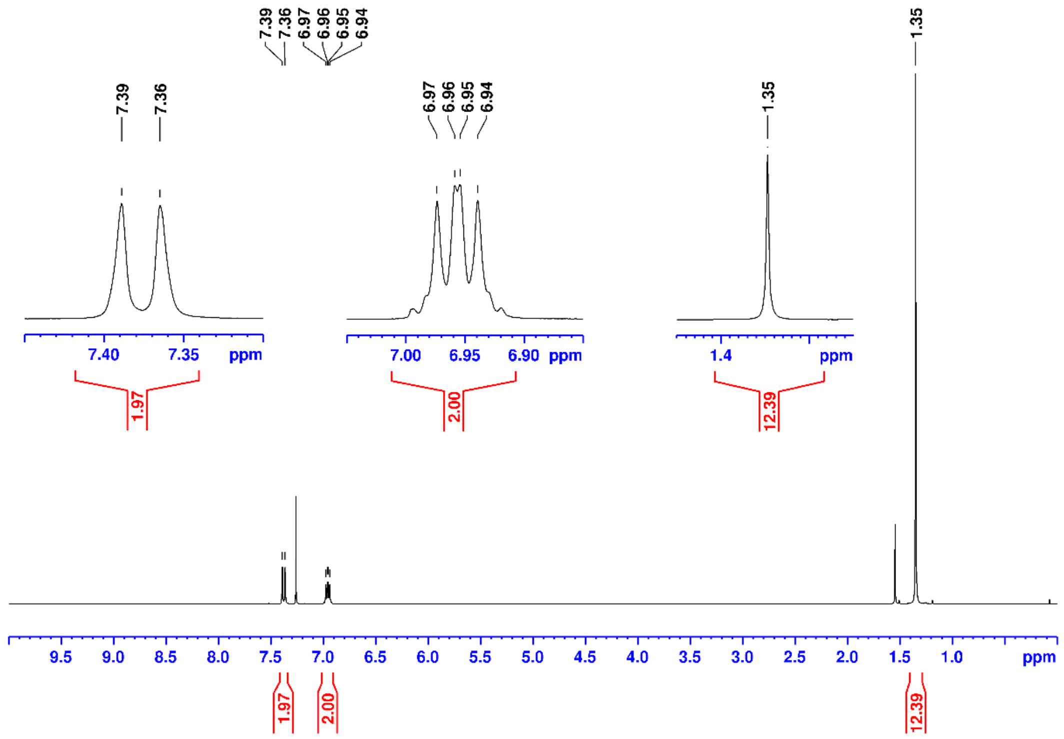

Figure S25: ^{1}H NMR spectrum of **5** in $CDCl_3$.

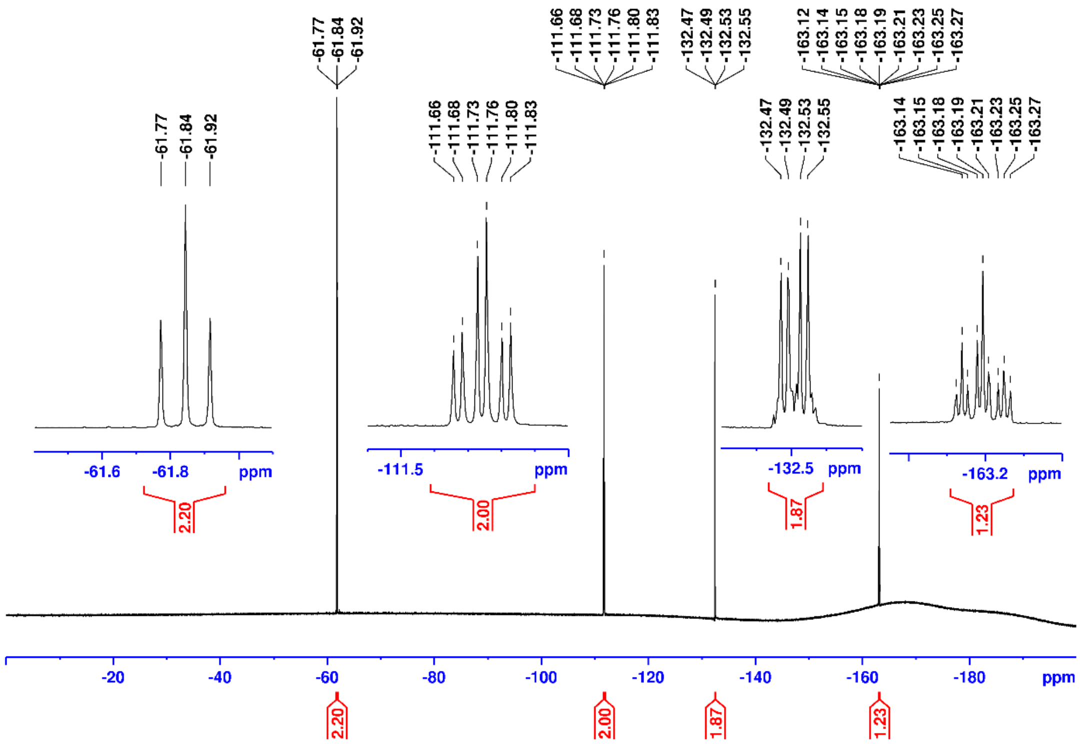

Figure S26: ^{19}F NMR spectrum of **5** in $CDCl_3$.

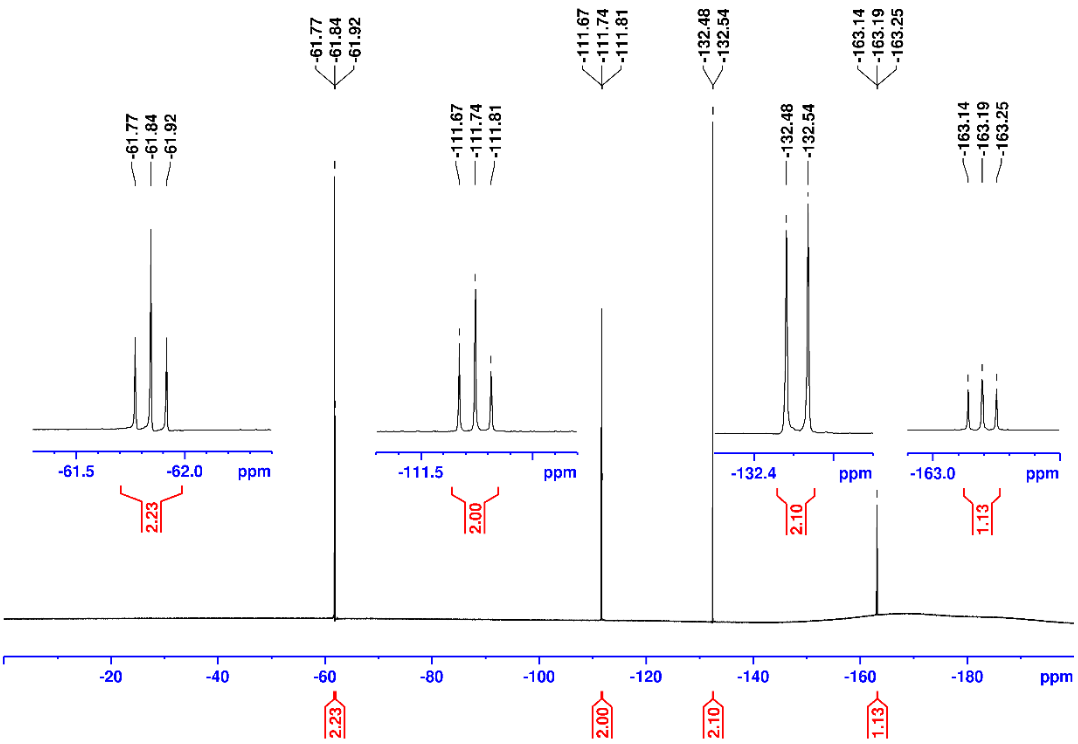

Figure S27: $^{19}F\{^1H\}$ NMR spectrum of **5** in $CDCl_3$.

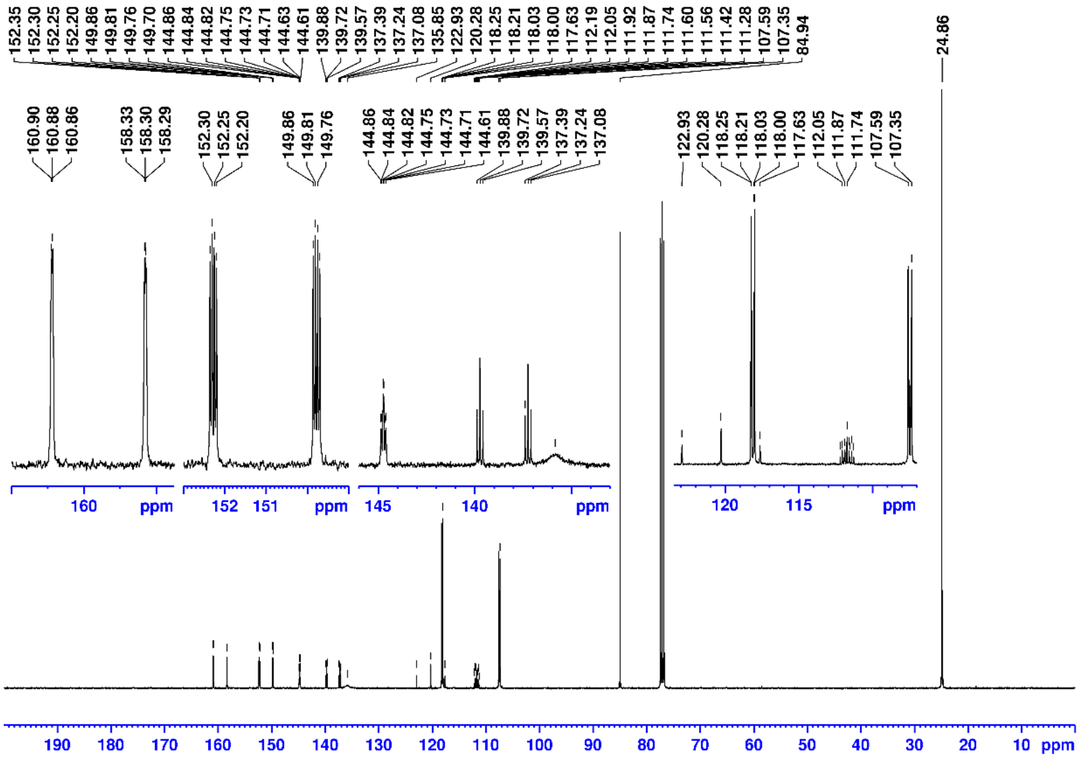

Figure S28: ^{13}C NMR spectra of **5** in $CDCl_3$.

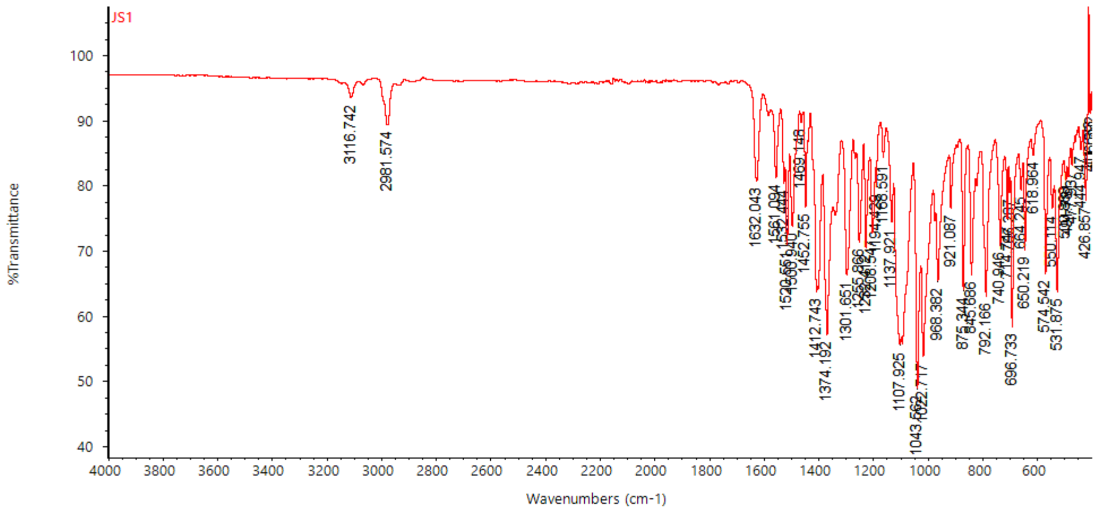

Figure S29: IR spectrum of **5**.

4'-(difluoro(3,4,5-trifluorophenoxy)methyl)-2,3',5'-trifluoro-[1,1'-biphenyl]-4-ol (6)[3]

Anhydrous potassium phosphate (3.41 g, 16.1 mmol, 3.50 eq.) was dissolved in 2.78 mL of water to form a solution of potassium phosphate trihydrate. **5** (2.00 g, 4.59 mmol, 1.00 eq.) and 4-bromo-3-fluorophenol (946 mg, 4.95 mmol, 1.08 eq.) were dissolved in 40 mL of THF and the potassium phosphate trihydrate solution was added. The mixture was sparged with argon for 30 min and then refluxed for 1 h. SPhos-ligand (188 mg, 459 µmol, 0.10 eq.) and palladium acetate (61.8 mg, 275 µmol, 0.06 eq.) were added and it was refluxed for 4 hours. After cooling down to rt, the mixture was acidified with 1 M HCl to pH = 4 and extracted with DCM (3 x 50 mL). The combined organic extracts were dried over $MgSO_4$ and the solvent was removed under reduced pressure. The crude product in form of an orange oil was further purified by column chromatography (DCM : *n*-hexane : ethyl acetate 2:7:1, Rf = 0.30) and recrystallisation in hexane.

Yield: 347 mg (18%), off-white crystalline powder.

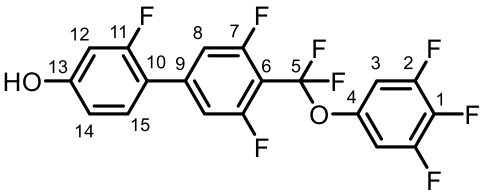

^{1}H NMR (400 MHz, $CDCl_3$): δ = 7.31 (app. t, $^3J_{HH}$ = 8.64 Hz, 1H, *H*-Ar), 7.17 (d, $^3J_{HF}$ = 11.2 Hz, 2H, *H*-8), 6.98 (dd, $^3J_{HF}$ = 7.88 Hz, $^4J_{HF}$ = 5.94 Hz,

2H, H-3), 6.76 – 6.68 (m, 2H, H-Ar) ppm. **^{19}F NMR** (376 MHz, 298 K, $CDCl_3$): δ = −61.7 (t, $^4J_{FF}$ = 26.2 Hz, 2F, F-5), −111.7 (td, $^4J_{FF}$ = 26.3 Hz, $^3J_{HF}$ = 10.5 Hz, 2F, F-7), −114.4 (m, 1F, F-11), −132.5 (dd, $^3J_{FF}$ = 20.6 Hz, $^3J_{HF}$ = 8.16 Hz, 2F, F-2), −163.5 (tt, $^3J_{FF}$ = 21.1 Hz, $^4J_{HF}$ = 5.99 Hz, 1F, F-1) ppm. **^{13}C NMR** (100 MHz, $CDCl_3$): δ = 160.4 (d, $^1J_{CF}$ = 251 Hz, C-11), 159.9 (dm, $^1J_{CF}$ = 257 Hz, C-7), 157.8 (d, $^2J_{CF}$ = 11.7 Hz), 151.0 (app. dq, $^1J_{CF}$ = 251 Hz, $^2J_{CF}$ = 5.26 Hz, C-2), 144.7 (tt, $^2J_{CF}$ = 11.7 Hz, $^2J_{CF}$ = 2.20 Hz, C-6), 141.5 (t, $^2J_{CF}$ = 11.2 Hz), 138.5 (dt, $^1J_{CF}$ = 250 Hz, $^2J_{CF}$ = 15.2 Hz, C-1), 130.9 (d, $^2J_{CF}$ = 4.40 Hz), 120.3 (t, $^1J_{CF}$ = 266 Hz, C-5), 118.2 (dt, $^2J_{CF}$ = 12.5 Hz, $^3J_{CF}$ = 2.20 Hz), 112.6 (dt, $^2J_{CF}$ = 24.0 Hz, $^3J_{CF}$ = 23.35 Hz), 112.2 (d, $^3J_{CF}$ = 2.94 Hz), 108.1 (m, C-4), 107.5 (m), 104.2 (d, $^2J_{CF}$ = 25.8 Hz) ppm.

IR (ν_{max}/cm^{-1}): 3432 (−O−H), 3098 (=C−H), 1621 (C=C), 1019 (C−F) cm^{-1}.

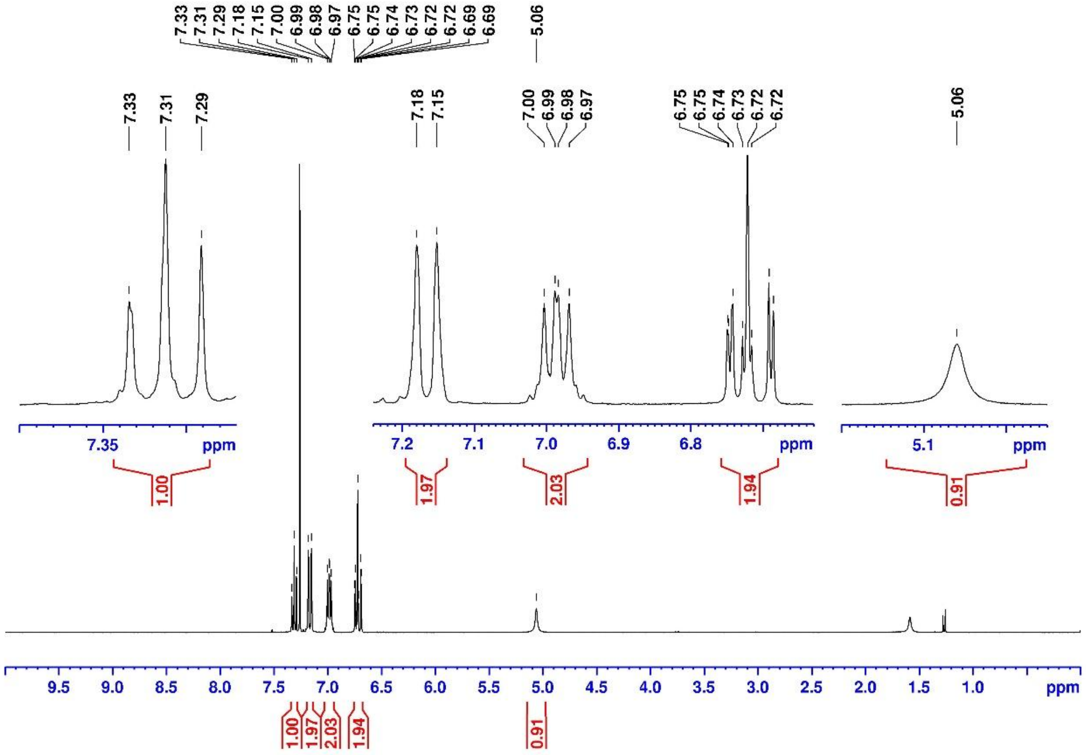

Figure S30: ^{1}H NMR spectrum of **6** in $CDCl_3$.

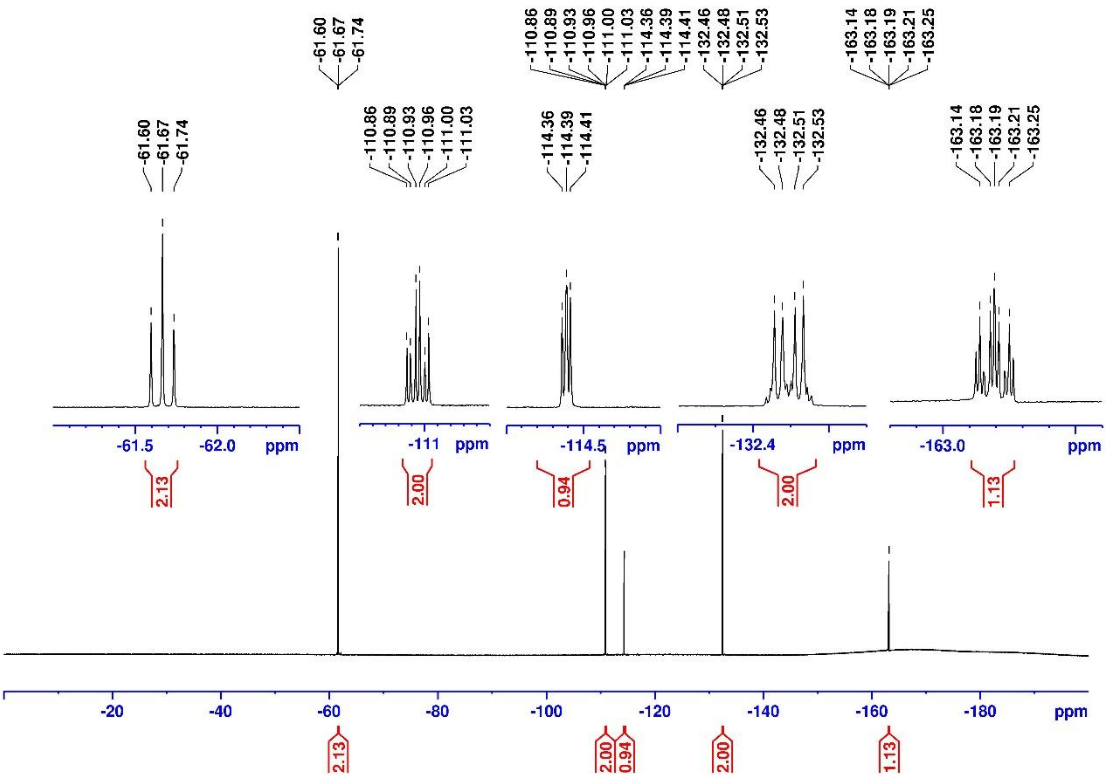

Figure S31: ^{19}F NMR spectrum of **6** in $CDCl_3$.

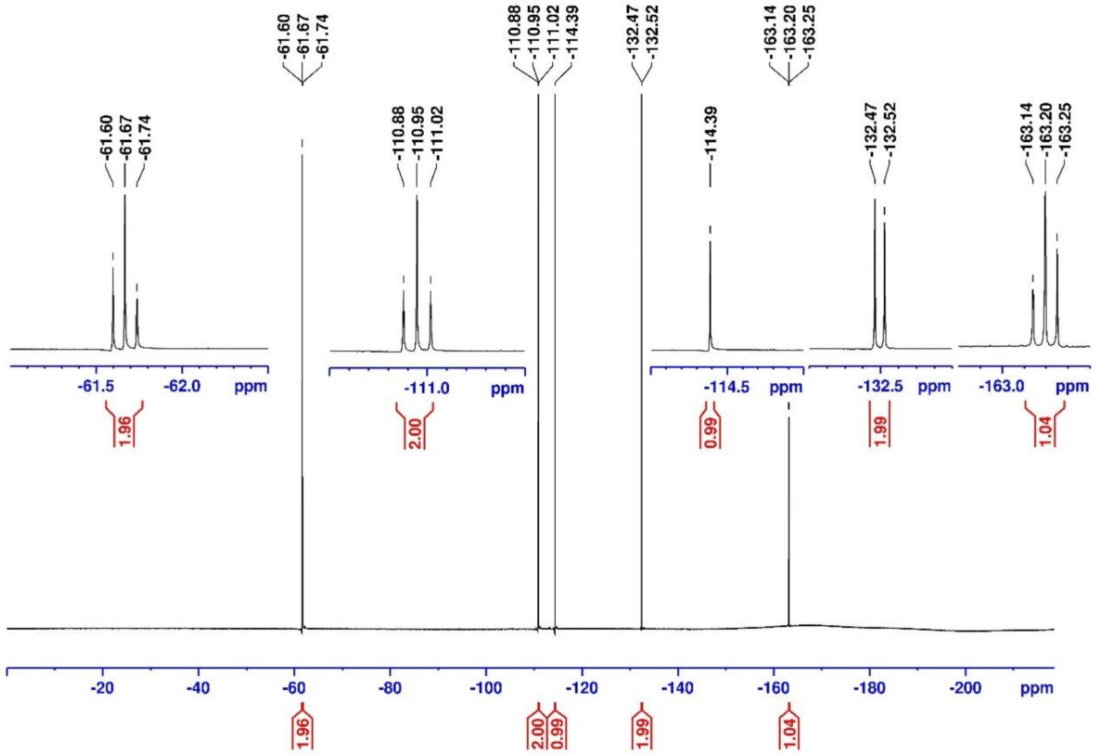

Figure S32: $^{19}F\{^1H\}$ NMR spectrum of **6** in $CDCl_3$.

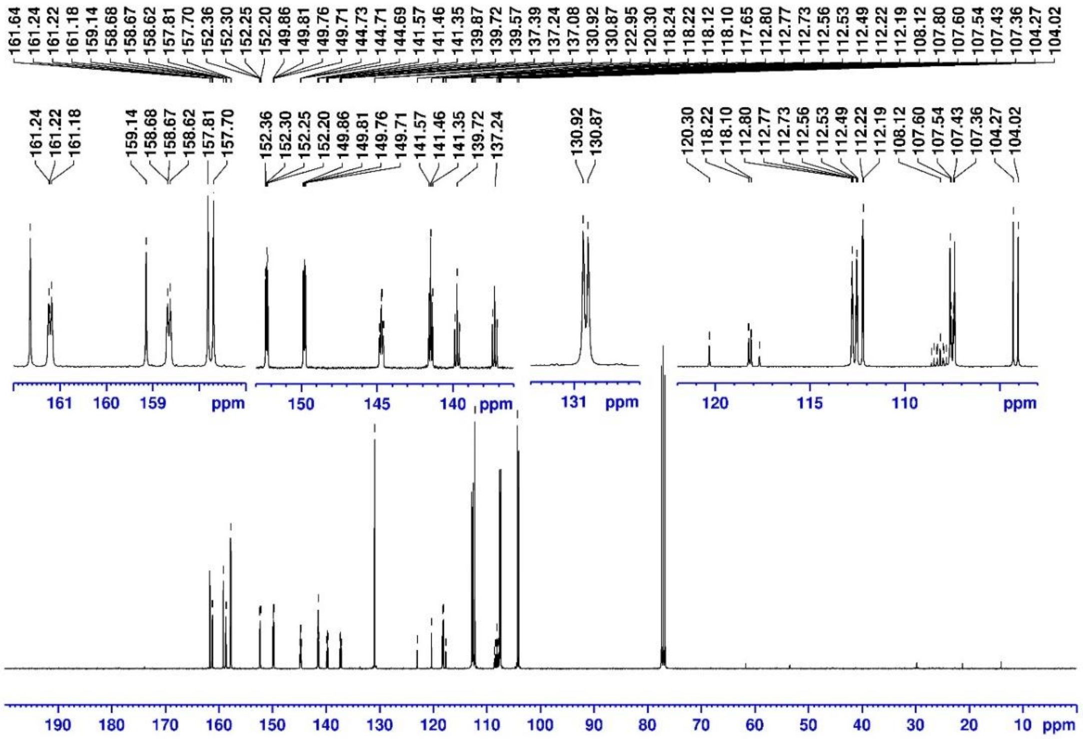

Figure S33: ^{13}C NMR spectrum of **6** in $CDCl_3$.

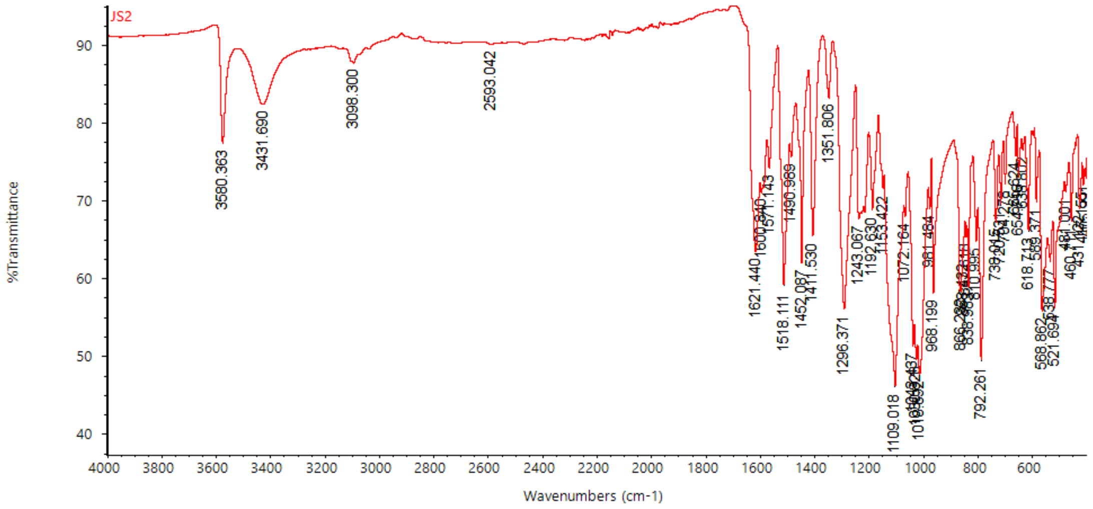

Figure S34: IR spectrum of **6.**

4'-(difluoro(3,4,5-trifluorophenoxy)methyl)-2,3',5'-trifluoro-[1,1'-biphenyl]-4-yl (S)-2,6-difluoro-4-(5-(2-methylbutyl)-1,3-dioxan-2-yl)benzoate (7(S), S-RW4*)

(S)-2,6-Difluoro-4-(5-(2-methylbutyl)-1,3-dioxan-2-yl)benzoic acid (**4**, 100 mg, 318 µmol, 1.00 eq.) was dissolved in 10 mL of DCM and DCC (85.3 mg, 414 µmol, 1.30 eq.) were added and the mixture was stirred until it changed to opaque (2 min). **6** (147 mg, 350 µmol, 1.10 eq.) and DMAP (5.05 mg, 41.4 µmol, 0.13 eq.) were added which created a clean mixture again. It was stirred for 22 h at rt. During the reaction a white precipitant formed, which was removed through filtration. The filtrate was dried in vacuo. The crude product was purified by column chromatography (DCM : petroleum ether 1:1, Rf = 0.62) and recrystallisation in ethanol.

Yield: 136 mg (60%), white solid.

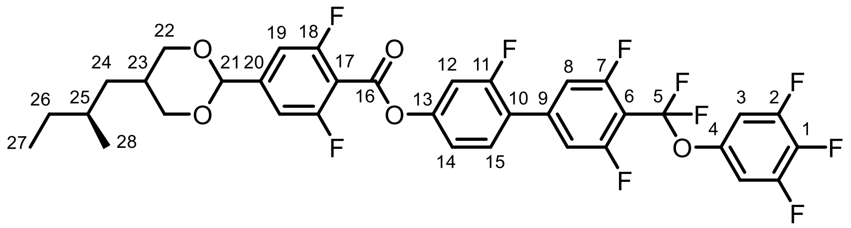

^{1}H-NMR (400 MHz, $CDCl_3$): δ = 7.49 (app. t, $^{3}J_{HF}$ = 8.57 Hz, 1H, *H*-Ar), 7.25 – 7.17 (m, 6H, *H*-Ar), 7.00 (dd, $^{3}J_{HF}$ = 7.67 Hz, $^{4}J_{HF}$ = 5.99 Hz, 2H, *H*-3), 5.41 (s, 1H, *H*-21), 4.29 - 4.19 (m, 2H, *H*-22), 3.58 – 3.48 (m, 2H, *H*-22), 2.32 – 2.16 (m, 1H, *H*-23), 1.43 – 1.29 (m, 2H, *H*-24), 1.27 – 1.04 (m, 2H, *H*-Al), 0.96 – 0.85 (m, 7H, *H*-Al) ppm. **^{19}F-NMR** (376 MHz, 298 K, $CDCl_3$): δ = −61.8 (t, $^{4}J_{FF}$ = 26.3 Hz, 2F, *F*-5), −108.4 (d, $^{3}J_{HF}$ = 8.79 Hz, 2F, *F*-18), −110.3 (td, $^{4}J_{FF}$ = 26.5 Hz, $^{3}J_{HF}$ = 10.2 Hz, 2F, *F*-7), −113.6 (app. t, $^{3}J_{HF}$ = 9.54 Hz 1F, *F*-11), −132.4 (dd, $^{3}J_{FF}$ = 20.7 Hz, $^{3}J_{HF}$ = 8.08 Hz, 2F, *F*-2), −163.5 (tt, $^{3}J_{FF}$ = 20.4 Hz, $^{4}J_{HF}$ = 5.45 Hz, 1F, *F*-1) ppm. **^{13}C-NMR** (101 MHz, $CDCl_3$) δ ppm: 161.09 (dd, $J_{C\text{-}F\text{-}i}$ = 258.5 Hz, $J_{C\text{-}F\text{-}m}$ = 5.7 Hz), 160.09 (dd, $J_{C\text{-}F\text{-}i}$ = 257.8 Hz, $J_{C\text{-}F\text{-}m}$ = 6.0 Hz), 159.65 (d, $J_{C\text{-}F\text{-}i}$ = 252.5 Hz), 159.33 (t, $J_{C\text{-}F\text{-}l.r.c.}$ = 1.9 Hz), 151.77 (d, $J_{C\text{-}F\text{-}m}$ = 11.1 Hz), 151.16 (ddd, $J_{C\text{-}F\text{-}i}$ = 251.0 Hz, $J_{C\text{-}F\text{-}o}$ = 10.7 Hz, $J_{C\text{-}F\text{-}m}$ = 5.3 Hz), 145.87 (t, $J_{C\text{-}F\text{-}m}$ = 9.9 Hz), 145.04 – 144.40 (m), 140.80 (app. t, $J_{C\text{-}F\text{-}m}$ = 11.1 Hz), 138.62 (dt, $J_{C\text{-}F\text{-}i}$ = 250.4 Hz, $J_{C\text{-}F\text{-}o}$ = 15.2 Hz), 130.75 (d, $J_{C\text{-}F\text{-}m}$ = 3.8 Hz), 123.99 – 123.48 (m), 120.27 (t, $J_{C\text{-}F\text{-}i}$ = 266.3 Hz), 118.48 (d, $J_{C\text{-}F\text{-}p}$ = 3.7 Hz), 113.27 (dt, $J_{C\text{-}F\text{-}o}$ = 24.4 Hz, $J_{C\text{-}F\text{-}m}$ = 3.5 Hz), 110.97 (d, $J_{C\text{-}F\text{-}o}$ = 26.0 Hz), 110.46 (dd, $J_{C\text{-}F\text{-}o}$ = 23.8, $J_{C\text{-}F\text{-}p}$ = 3.3 Hz), 109.46 (t, $J_{C\text{-}F\text{-}o}$ = 16.9 Hz), 109.17 – 108.63 (m), 107.81 – 107.33 (m), 98.92 (t, $J_{C\text{-}F\text{-}l.r.c.}$ = 2.2 Hz), 73.05, 72.81, 35.07, 31.98, 31.35, 29.80, 19.41, 11.33.

IR (ν_{max}/cm^{-1}): 3100 (=C−H), 2963 (−C−H), 2919 (−C−H), 2876 (−C−H), 1748 (C=O), 1639 (C=C), 1033 (C−F) cm^{-1}.

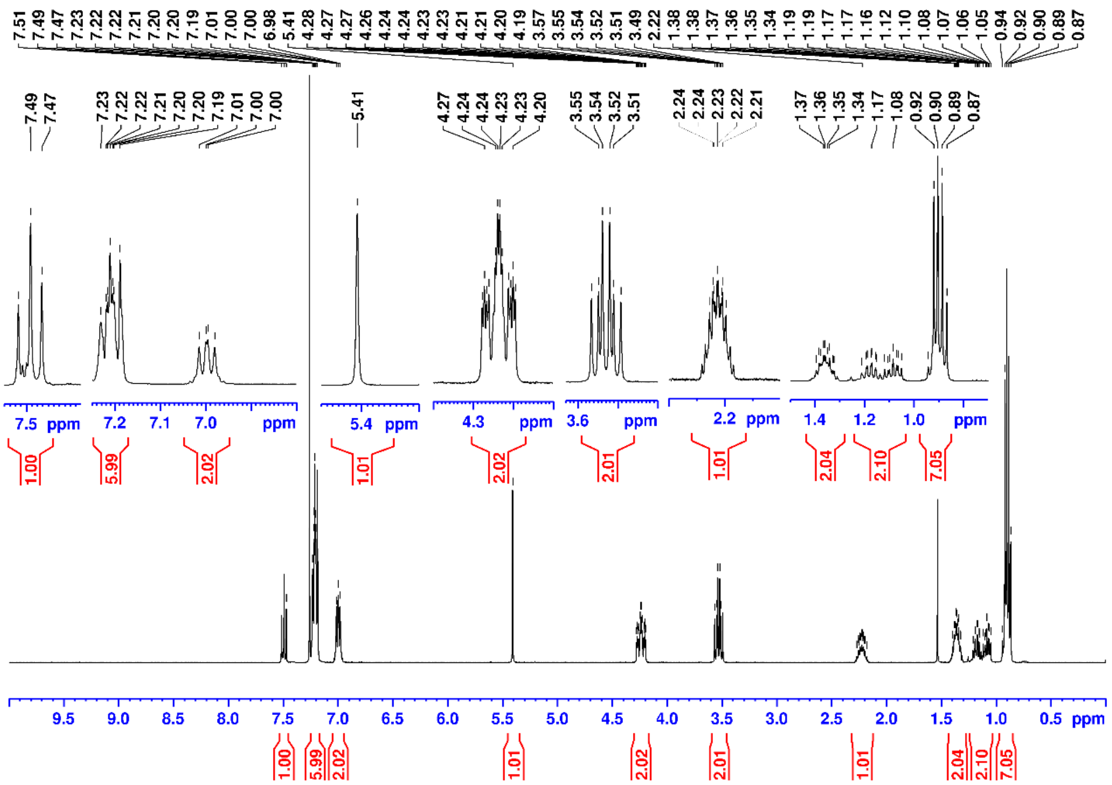

Figure S35: ^{1}H NMR spectrum of compound **S-RW4*** in $CDCl_3$.

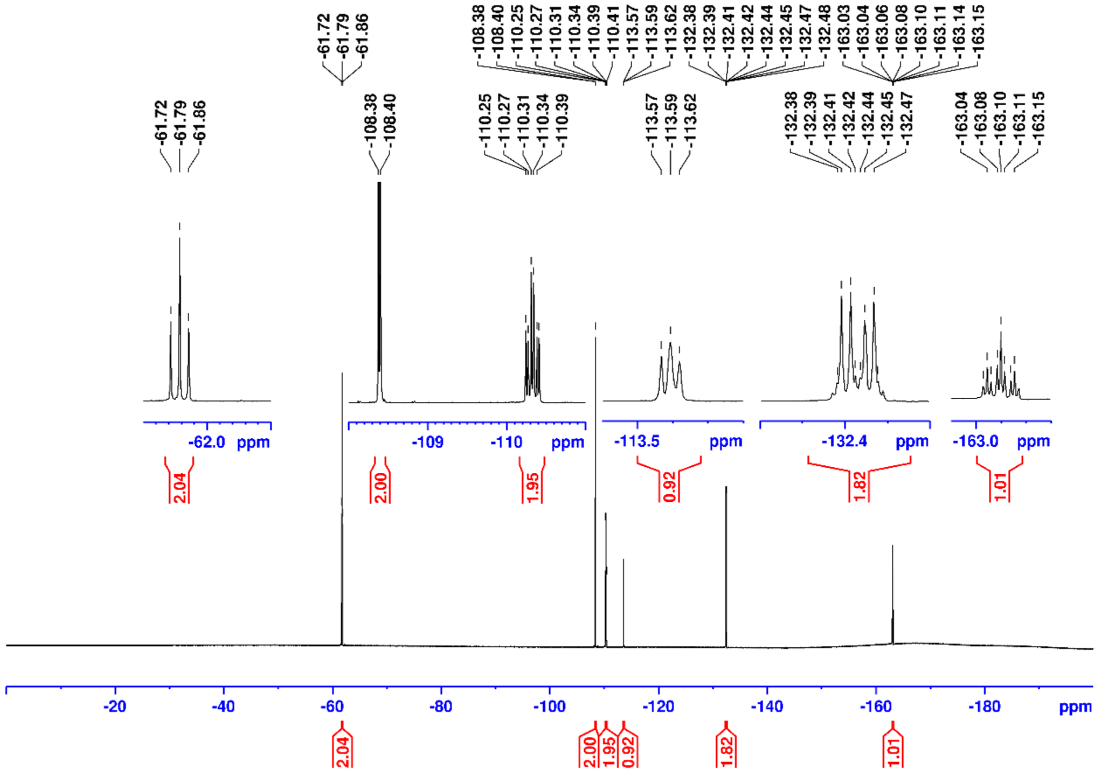

Figure S36: ^{19}F NMR spectrum of compound **S-RW4*** in $CDCl_3$.

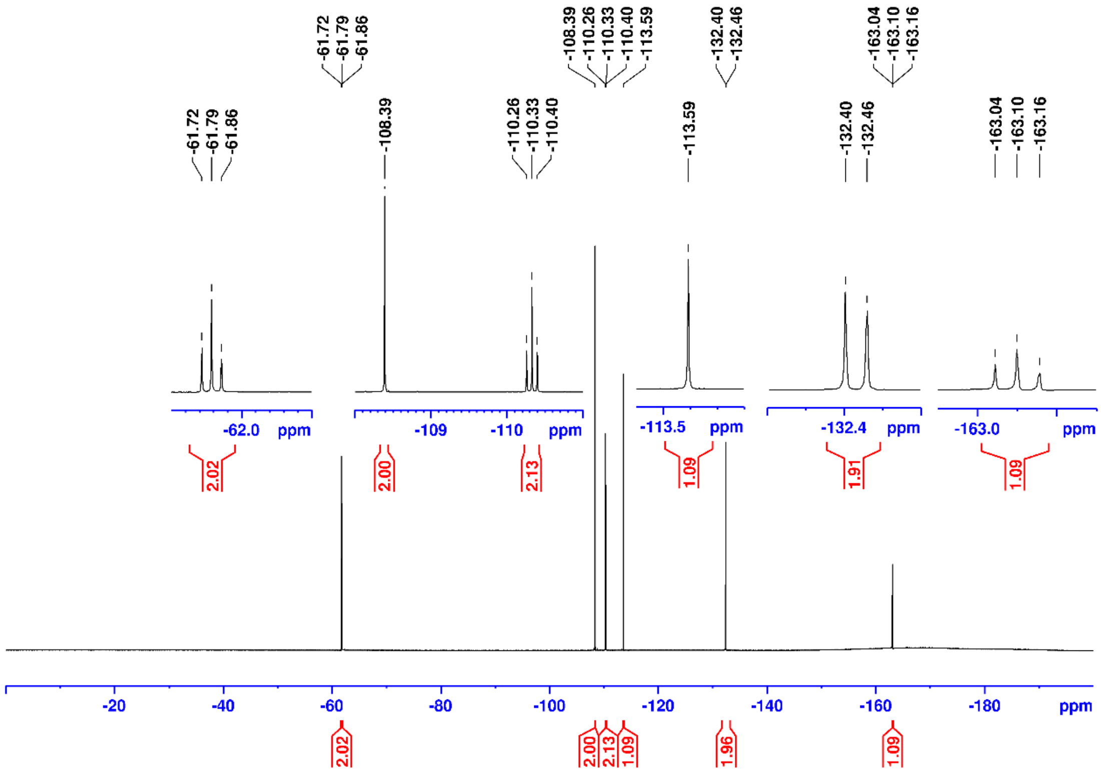

Figure S37: $^{19}F\{^1H\}$ NMR spectrum of **S-RW4***in $CDCl_3$.

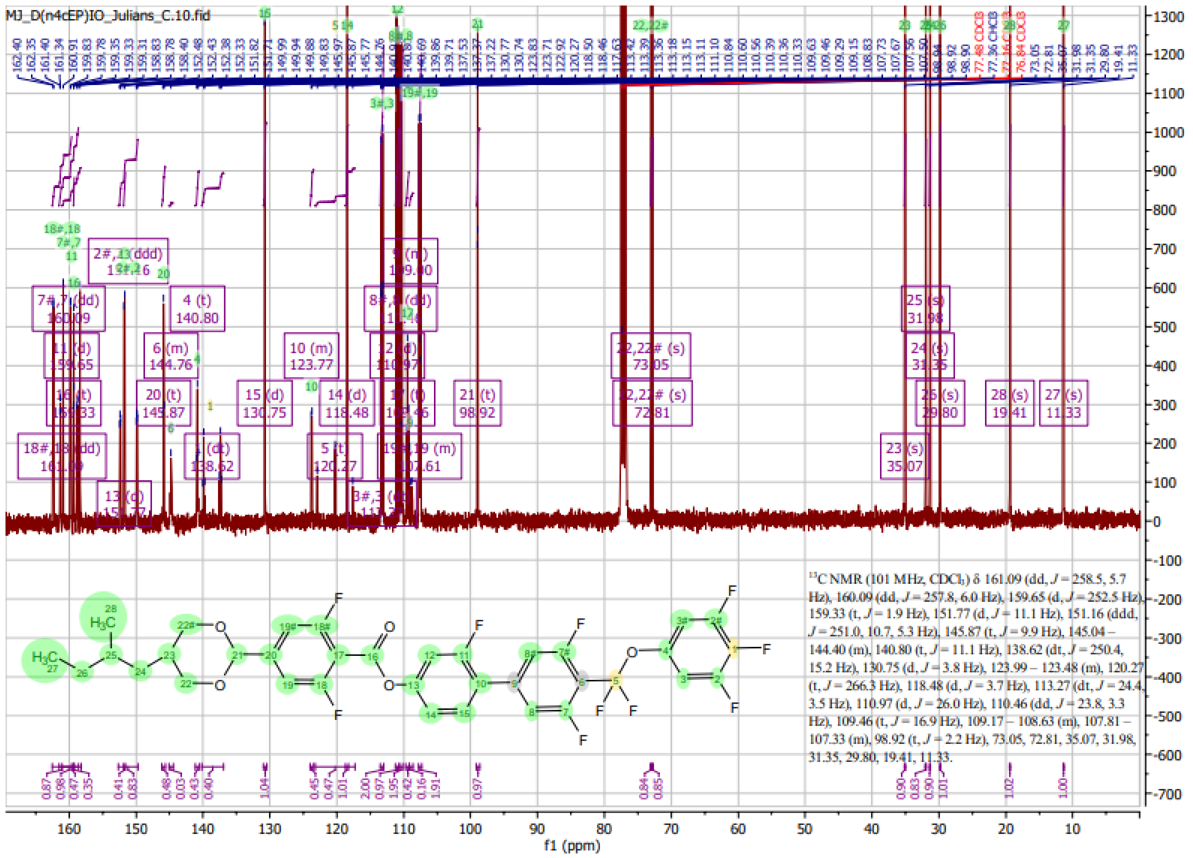

Figure S38: ^{13}C NMR spectrum of **S-RW4***in CDCl$_3$.

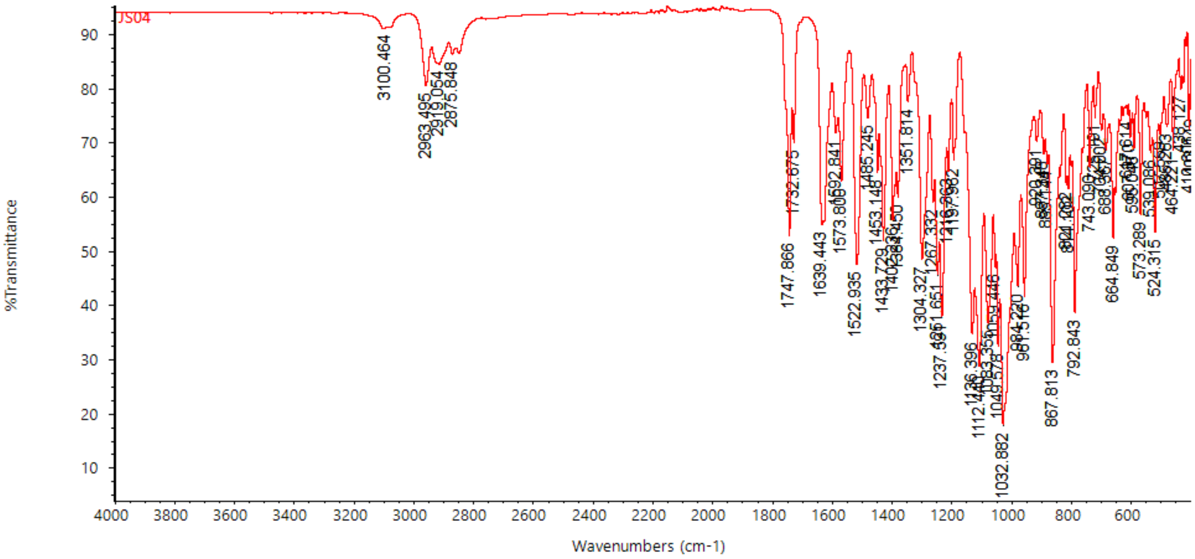

Figure S39: IR spectrum of compound **S-RW4***.

4'-(difluoro(3,4,5-trifluorophenoxy)methyl)-2,3',5'-trifluoro-[1,1'-biphenyl]-4-yl (+/-)-2,6-difluoro-4-(5-(2-methylbutyl)-1,3-dioxan-2-yl)benzoate (7(rac), rac-RW4*)

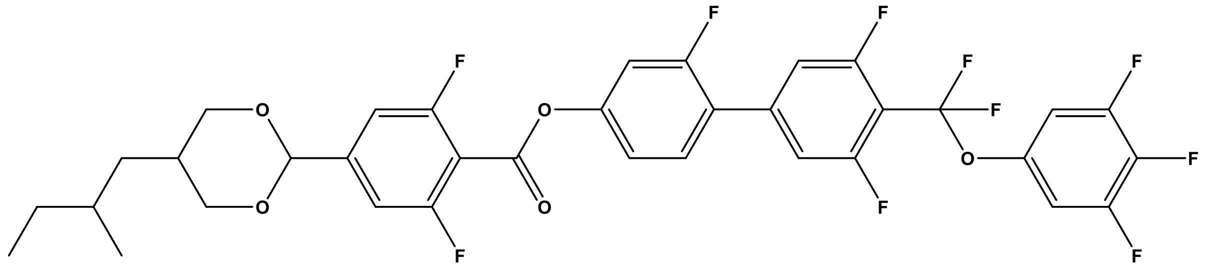

Prepared using the same method as the compound 7(S)

Yield: 37 mg (46%), white solid.

^{1}H-NMR (400 MHz, $CDCl_3$) δ ppm: 7.57 – 7.39 (1H, m, Ar-H), 7.26 – 7.11 (6H, m, Ar-H), 7.06 – 6.90 (2H, m, Ar-H), 5.41 (1H, s, Ar-CH), 4.31 – 4.16 (2H, m, -CH-CH_2-O-), 3.62 – 3.44 (2H, m, -CH-CH_2-O-), 2.32 – 2.09 (1H, m, , -CH_2-CH-(CH_2-O-)$_2$), 1.46 – 1.26 (2H,-CH-CH_2-CH- m,), 1.24 – 1.14 (1H, m,-CH-CH(-H)-CH_3), 1.13 – 1.03 (1H, m, -CH-CH(-H)-CH_3), 0.97 – 0.80 (7H, m, -(CH_2)$_2$-CH-CH_3; -CH_2-CH_3). **^{19}F-NMR** (376 MHz, CDCl3) δ ppm: -61.78 (t, J = 26.4 Hz), -108.39, -110.33 (t, J = 26.3 Hz), -113.60, -132.44 (d, J = 21.0 Hz), -163.11 (t, J = 20.7 Hz). **^{13}C-NMR** (101 MHz, $CDCl_3$) δ ppm: 161.09 (dd, $J_{C\text{-}F\text{-}i}$ = 258.5 Hz, $J_{C\text{-}F\text{-}m}$ = 5.7 Hz), 160.09 (dd, $J_{C\text{-}F\text{-}i}$ = 257.9 Hz, $J_{C\text{-}F\text{-}m}$ = 6.2 Hz), 159.65 (d, $J_{C\text{-}F\text{-}i}$ = 252.4 Hz), 159.33 (m), 151.77 (d, $J_{C\text{-}F\text{-}m}$ = 11.0 Hz), 151.16 (ddd, $J_{C\text{-}F\text{-}i}$ = 251.2 Hz, $J_{C\text{-}F\text{-}o}$ = 10.7 Hz, $J_{C\text{-}F\text{-}m}$ = 5.4 Hz), 145.87 (t, $J_{C\text{-}F\text{-}m}$ = 9.9 Hz), 145.04 – 144.40 (m), 140.80 (app. t, $J_{C\text{-}F\text{-}m}$ = 10.9 Hz), 138.62 (dt, $J_{C\text{-}F\text{-}i}$ = 250.3 Hz, $J_{C\text{-}F\text{-}o}$ = 15.3 Hz), 130.76 (d, $J_{C\text{-}F\text{-}m}$ = 3.7 Hz), 123.78 (d, J = 12.6 Hz), 120.27 (t, $J_{C\text{-}F\text{-}i}$ = 267.3 Hz), 118.49 (d, $J_{C\text{-}F\text{-}p}$ = 3.8 Hz), 113.63 – 112.78 (m), 110.97 (d, $J_{C\text{-}F\text{-}o}$ = 25.9 Hz), 110.46 (dd, $J_{C\text{-}F\text{-}o}$ = 23.7, $J_{C\text{-}F\text{-}p}$ = 3.3 Hz), 109.46 (t, $J_{C\text{-}F\text{-}o}$ = 16.9 Hz), 109.20 – 108.76 (m), 107.90 – 107.28 (m), 99.05 – 98.74 (m), 73.06, 72.81, 35.08, 31.99, 31.35, 29.81, 19.42, 11.33.

IR (ν_{max}/cm^{-1}): 3107 (=C−H), 2964 (−C−H), 2931 (−C−H), 2853 (−C−H), 1758 (C=O), 1635 (C=C).

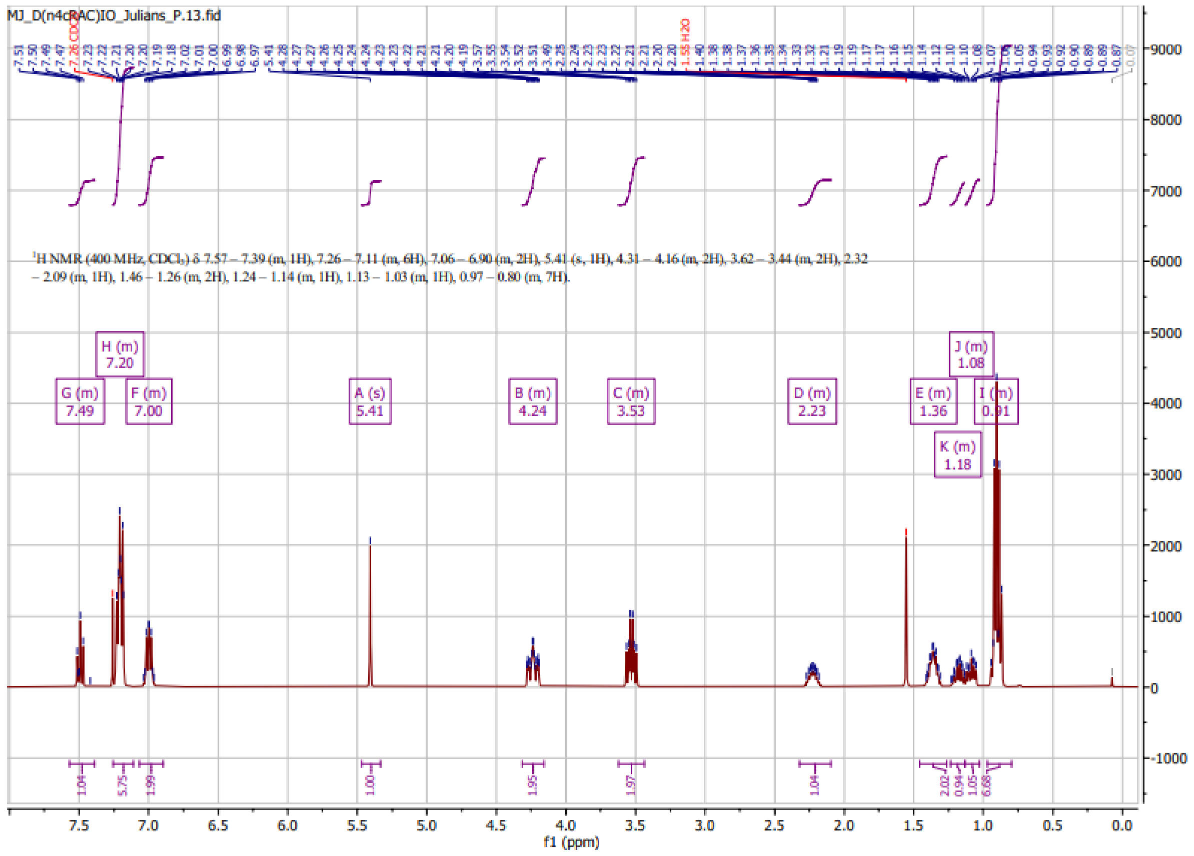

Figure S40: ^{1}H NMR spectrum of compound **rac-RW4*** in $CDCl_3$.

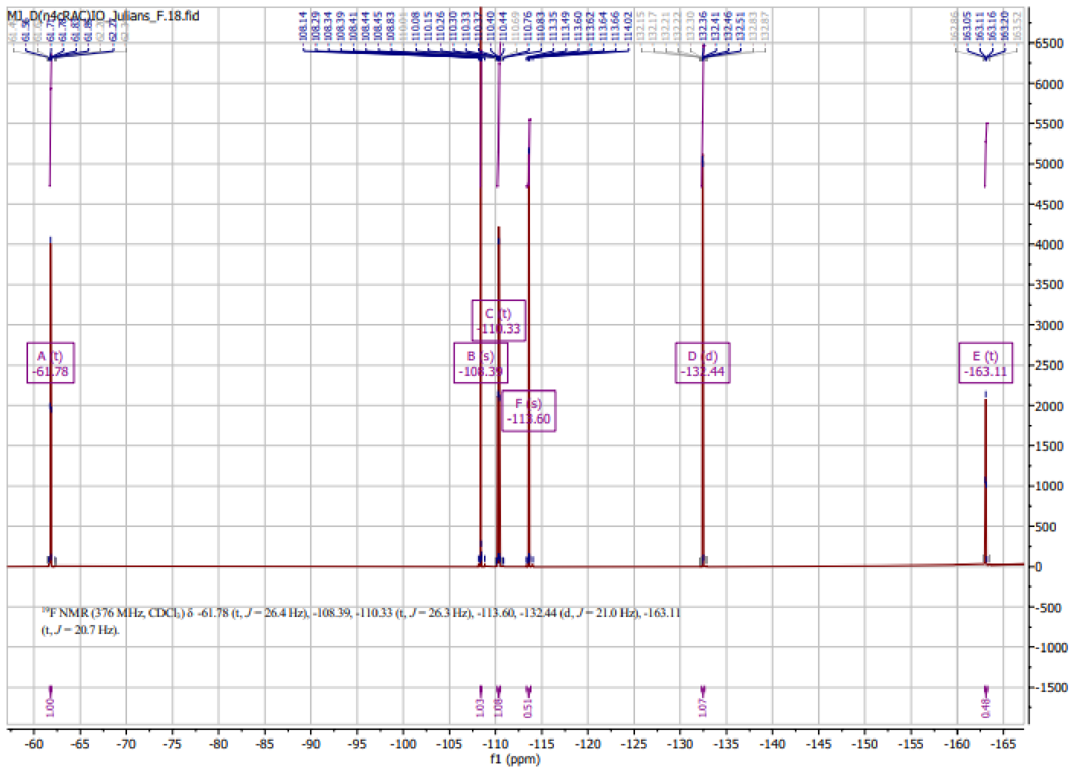

Figure 41: ^{19}F NMR spectrum of compound **rac-RW4*** in $CDCl_3$.

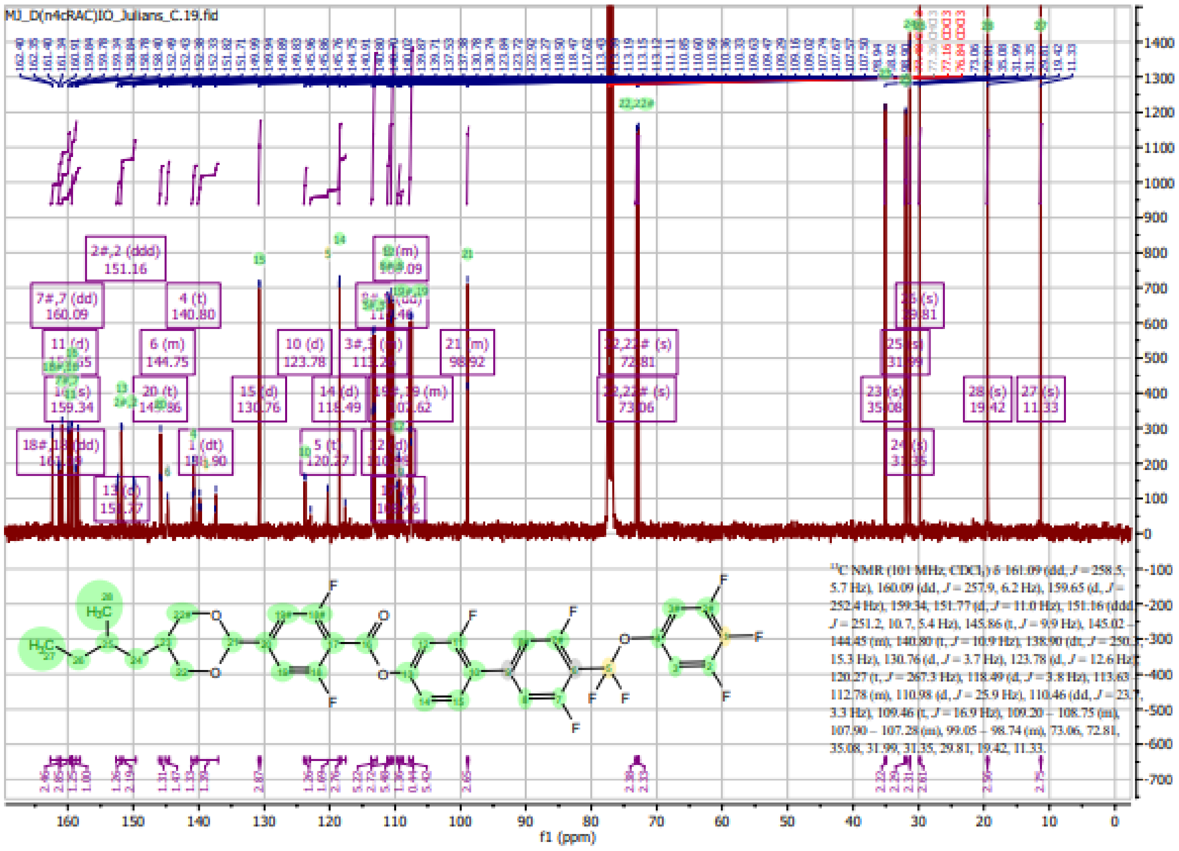

Figure S42: ^{13}C NMR spectrum of compound **rac-RW4*** in $CDCl_3$.

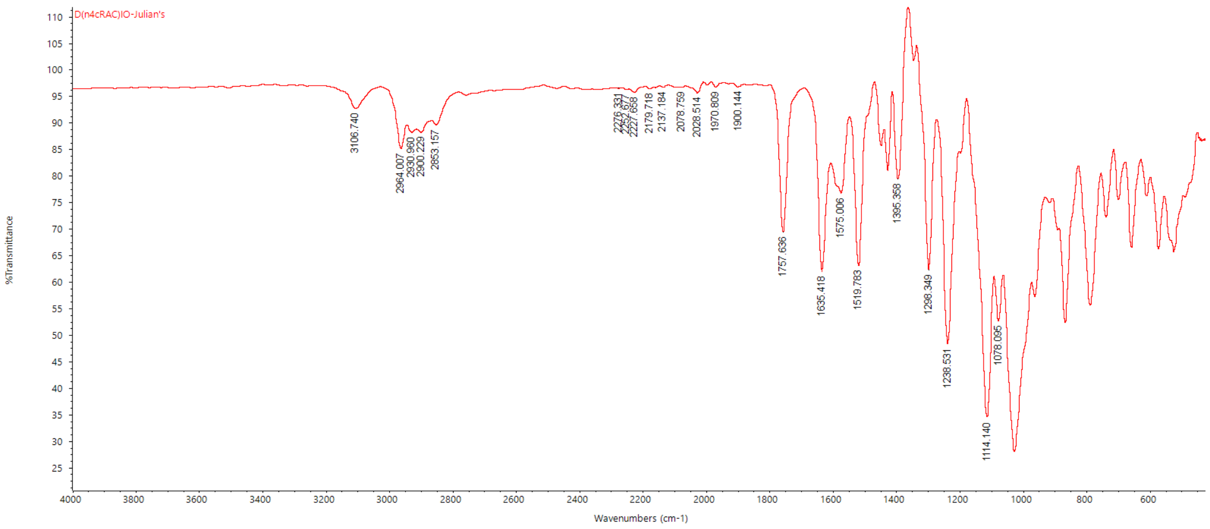

Figure S43: IR spectrum of compound **rac-RW4***.

Experimental methods

Calorimetric studies: Differential scanning calorimetry was performed using a Mettler Toledo DSC3 instrument equipped with TSO 801RO sample robots and calibrated using indium and zinc standards. Heating and cooling rates were 10 K min^{-1}, between heating and cooling runs a 3 min isotherm steps were applied. Samples were measured under a nitrogen atmosphere.

X-ray diffraction (XRD) studies in broad diffraction angle range were performed with Bruker GADDS system equipped with micro-focus type X-ray tube with Cu anode and dedicated Montel optics, diffraction patterns were recorded with Vantec 2000 area detector. Samples were prepared in the form of small droplets placed on a heated surface and their temperature was controlled with a modified Linkam heating stage. For precise determination of smectic layer spacing temperature evolution small angle X-ray diffraction experiments were performed, using Bruker Nanostar system (micro-focus type X-ray tube with Cu anode and dedicated Montel optics, MRI TCPU-H heating stage, Vantec 2000 area detector). Samples were prepared in thin-walled glass capillaries, with 1.5 mm diameter. The x-ray patterns was analyzed using TOPAS software.

Microscopic studies: Optical textures of LC phases were studied using a Zeiss Axio Imager A2m polarized light microscope, equipped with a Linkam LTS420 heating stage. Samples were prepared in commercial cells (AWAT) of various thicknesses (1.5–20 µm) with ITO electrodes and surfactant layers for planar or homeotropic alignment, in the case of planar cells parallel rubbing on both surfaces was applied. Cells for in-plane switching (~3-µm-thick, with planar alignment layers and 3 millimeter distance between electrodes) were provided by prof. O. Lavrentovich group at Kent State University.

Selective light reflection and absorption studies were carried out for material placed in the glass cell (3:10-µm-thick) with planar anchoring. The measurements were performed with a fiber-coupled spectrometer (Filmetrics F20-UV) mounted to the Zeiss Axio Imager A2m microscope working either in reflection or transmission mode. The tested sample area was confined to ~50 microns.

Optical birefringence measurements were measured with a setup based on a photoelastic modulator (PEM-90, Hinds) working at the base frequency f=50 kHz. As a light source, a halogen lamp (Hamamatsu LC8) equipped with a narrow bandpass filter (532±3 nm) was used. Samples were prepared in glass cells with a thickness of 1.5 µm, having surfactant layers for planar anchoring and parallel rubbing assuring uniform alignment of the optical axis in nematic phases. The sample and PEM were placed between crossed linear polarizers, with axes rotated ±45 deg with respect to the PEM axis, and the intensity of the light transmitted through this set-up was measured with a photodiode (FLC Electronics PIN-20). The registered signal was de-

convoluted with a lock-in amplifier (EG&G 7265) into 1f and 2f components to yield a retardation induced by the sample.

Dielectric spectroscopy: The complex dielectric permittivity was measured in the 1 Hz–10 MHz frequency range using a Solartron 1260 impedance analyzer. The material was placed in 3:10-μm-thick glass cell with gold electrodes (without surfactant to avoid the influence of the high capacitance of a thin polymer layer). The amplitude of the applied ac voltage, 20 mV, was low enough to avoid Fréedericksz transition in nematic phases.

Polarization current measurements: electric polarization measurements were performed using cells with gold electrodes and no surfactant layers. The spontaneous polarization was calculated by analyzing the current flow through a resistor (500 Ω) connected in series with the cell, upon application of triangular-wave voltage, saturation of the current vs. applied electric field in few temperatures was checked. Siglent SDG2042X arbitrary waveform generator, FLC A200 amplifier and Siglent SDS2000X Plus oscilloscope were used.

Second Harmonic Generation: Second Harmonic Generation: The SHG response was investigated using a setup based on a solid-state IR laser EKSPLA NL202, λ=1064 nm. A series of collimated (~1 mm 1/e2), 9-ns laser pulses at a 10 Hz repetition rate and <2 mJ pulse energy were applied. The pulse energy was adjusted to avoid material decomposition. The IR beam was incident onto an LC cell of thickness 10 μm. An IR pass filter was placed at the entrance to the sample and a green pass filter at the exit of the sample. The emitted SHG radiation was detected using a photon counting head (Hamamatsu H7421) with a power supply unit (C8137). The SHG signal intensity was monitored with an oscilloscope (Agilent Technologies DSO6034A) using a custom-written Python script. The samples with in-plane electrodes were used. Optical SHG images were recording with homemade microscopic setup.

Additional results

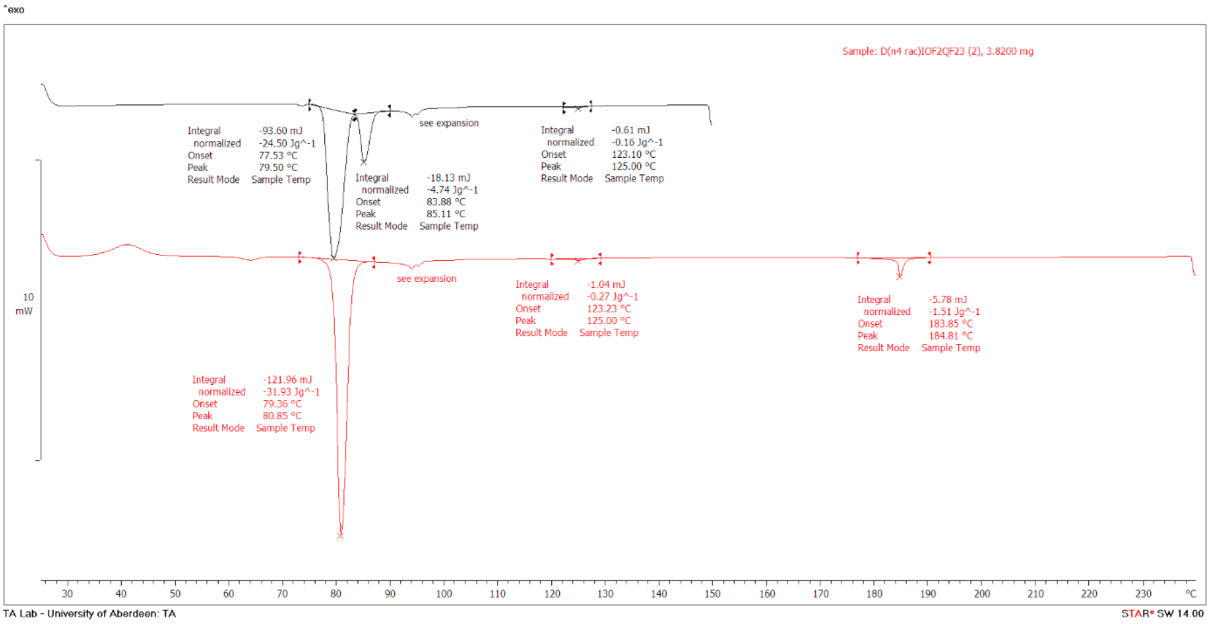

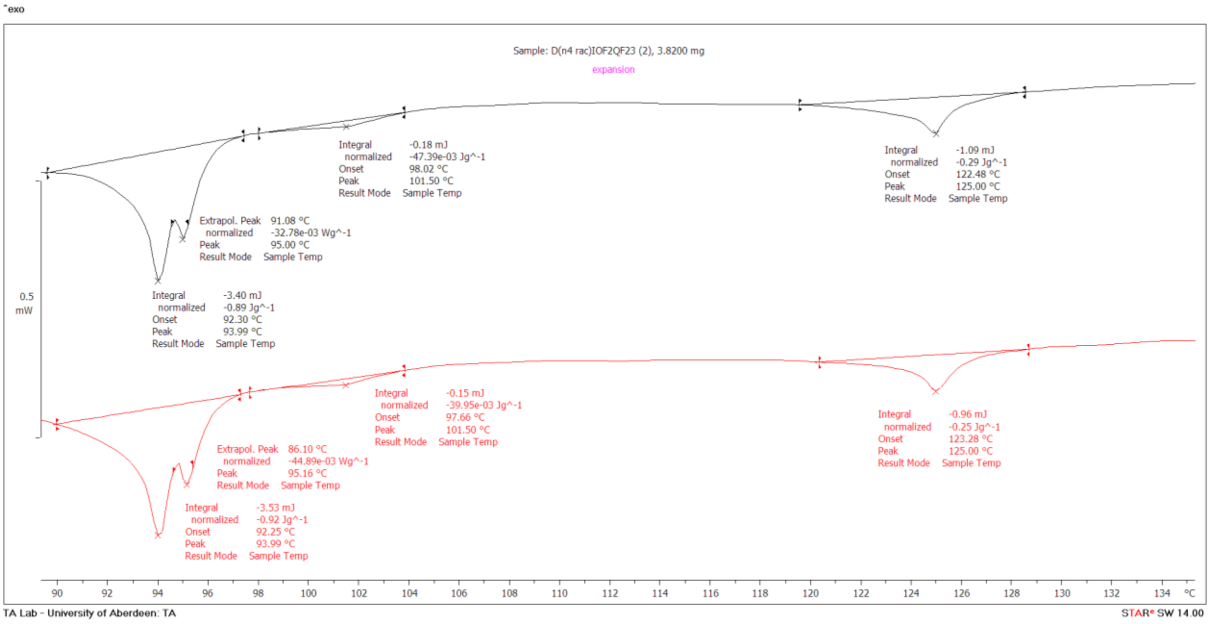

Figure S45: Heating DSC trace of **rac-RW4*,** full temperature range (top) and expanded region (bottom).

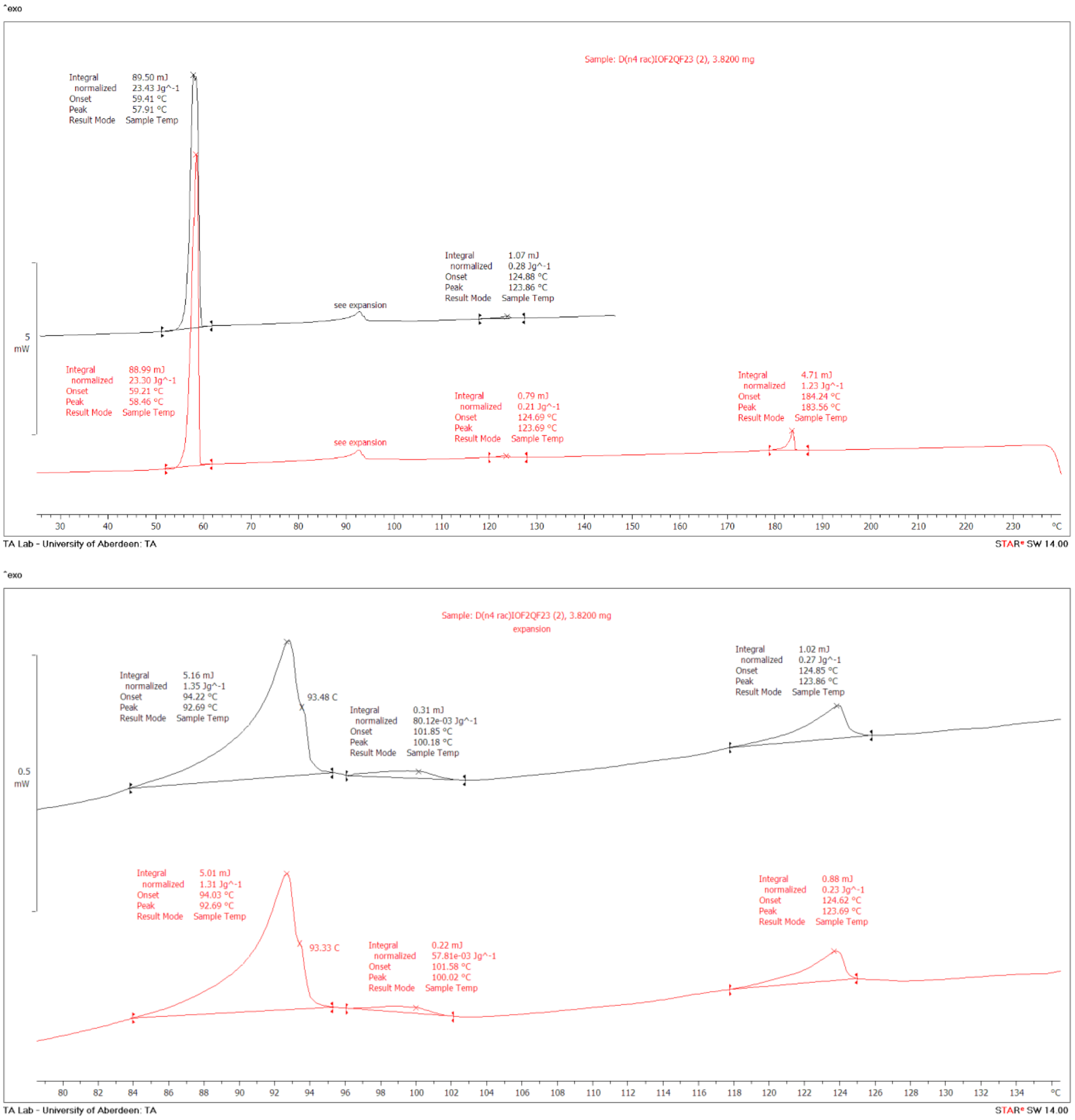

Figure S46: Cooling DSC trace of **rac-RW4*,** full temperature range (top) and expanded region (bottom).

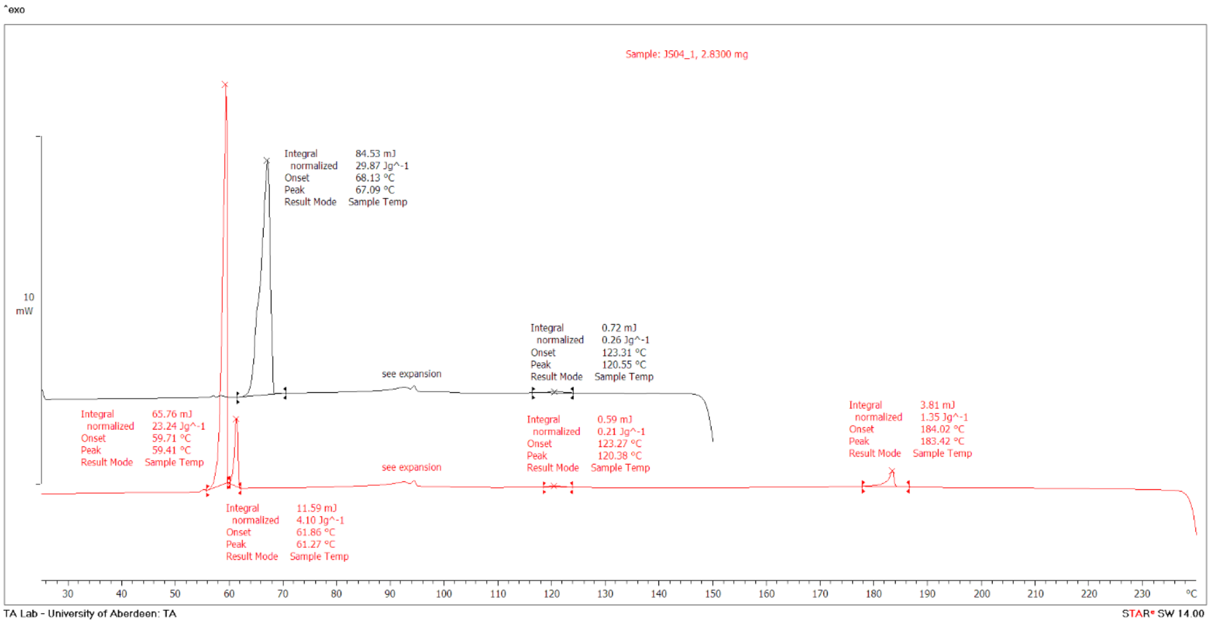

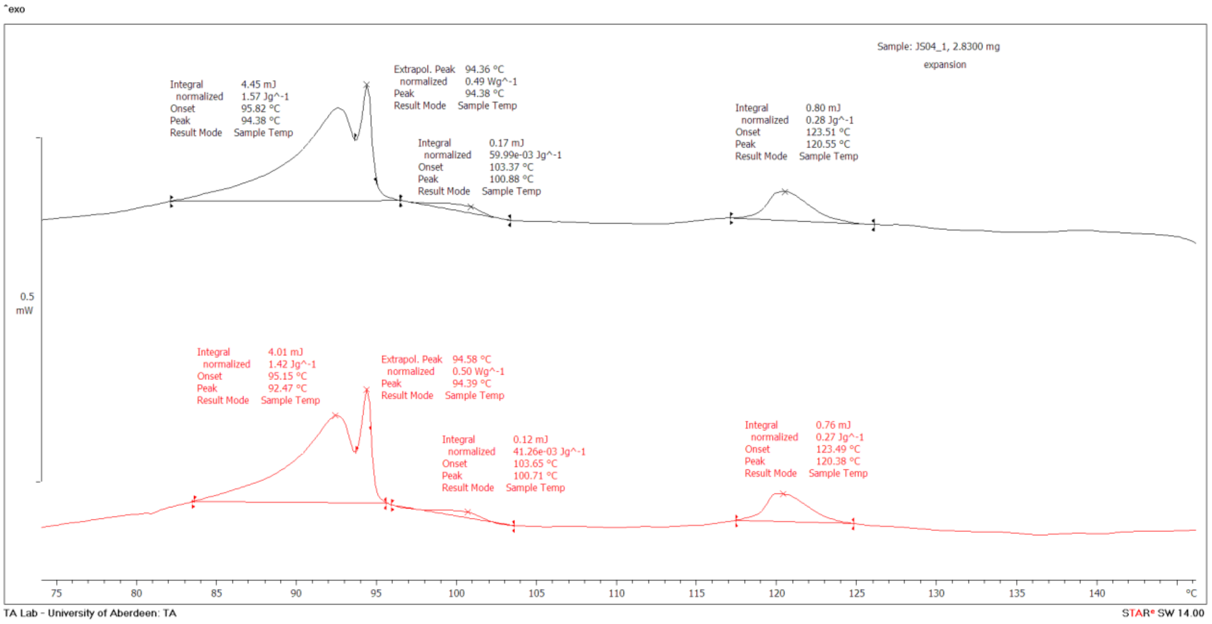

Figure S47: Cooling DSC trace of **S-RW4*,** full temperature range (top) and expanded region (bottom).

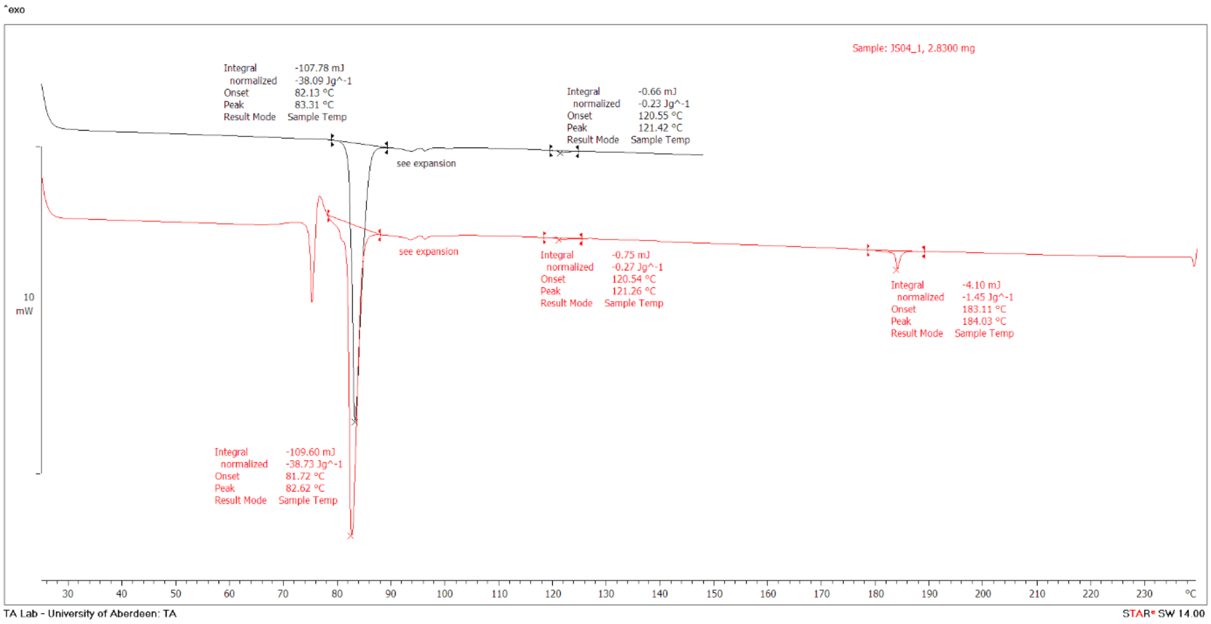

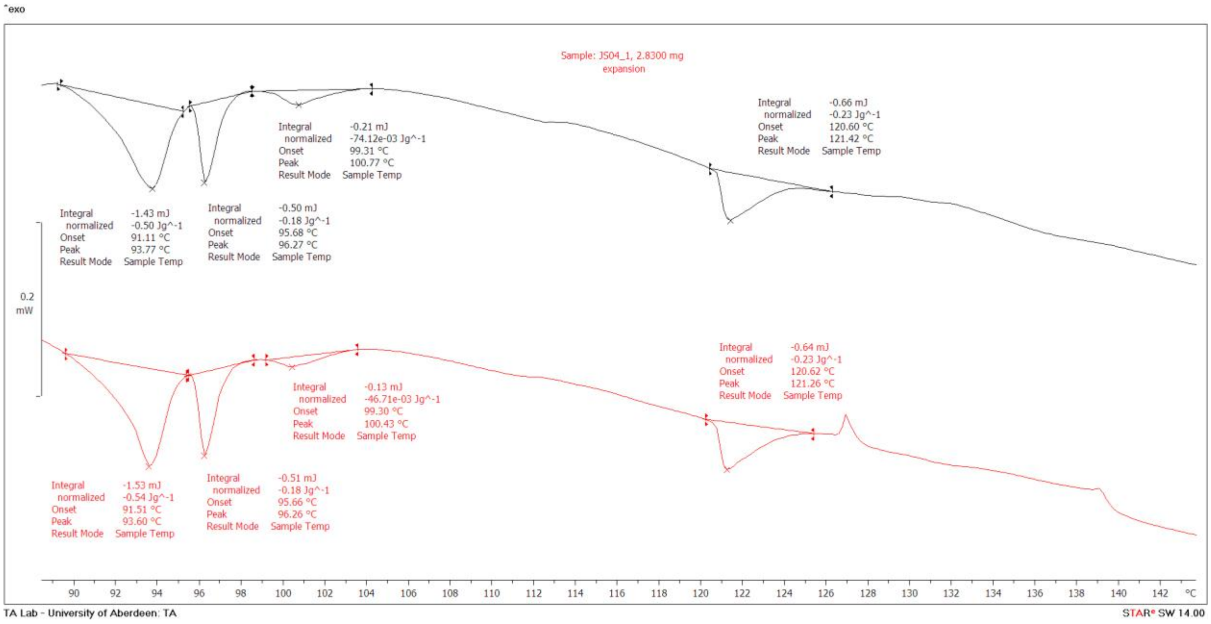

Figure S48: Heating DSC trace of **S-RW4***, full temperature range (top) and expanded region (bottom).

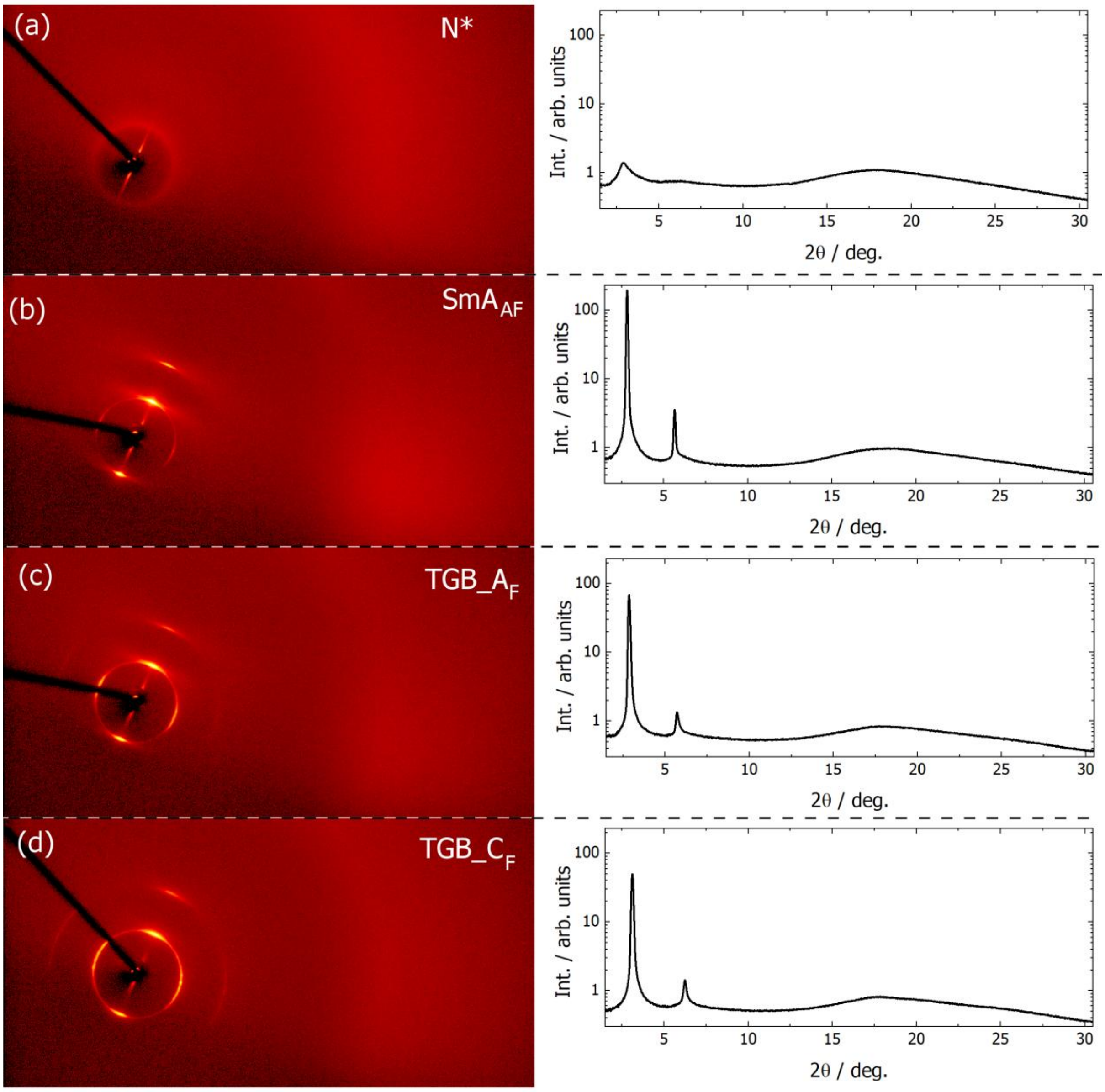

Figure S49: Wide angle X-ray diffraction patterns recorded in various phases of S-**RW4*** compound, diffractograms on the right were obtained by integration of 2D patterns presented on left, by integration over azimuthal angle.

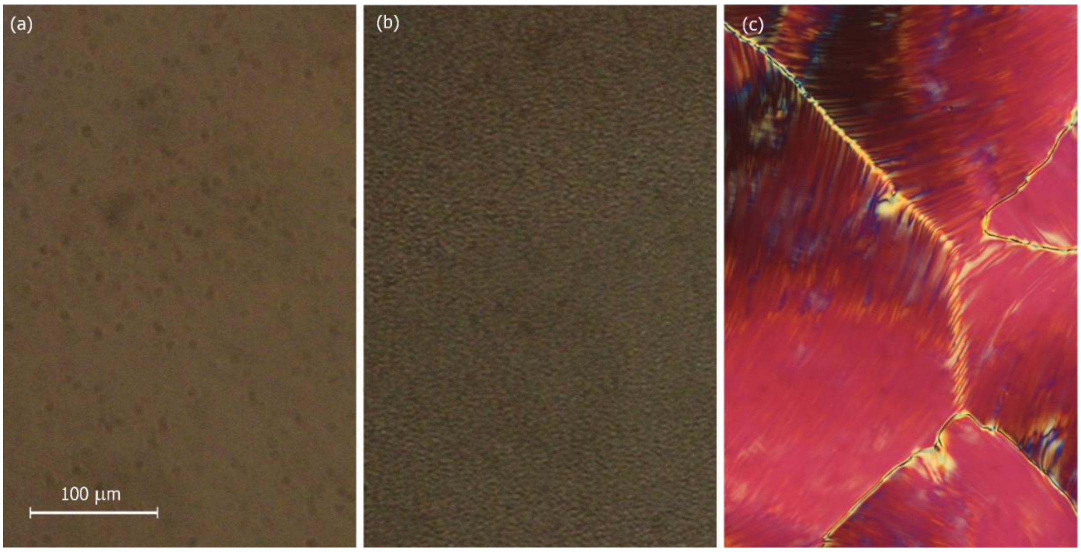

Figure S50: Optical texture of rac-**RW4*** compound observed in 3-µm-thick cell with homeotropic anchoring: in SmA_{AF} phase perfect homeotropic texture is obtained(a), in SmA_F phase small wrinkle-like defects develop (b) and the texture gradually rebuilds into planar one (c). Note, that the photos presented in panels (a) and (b) were overexposed.

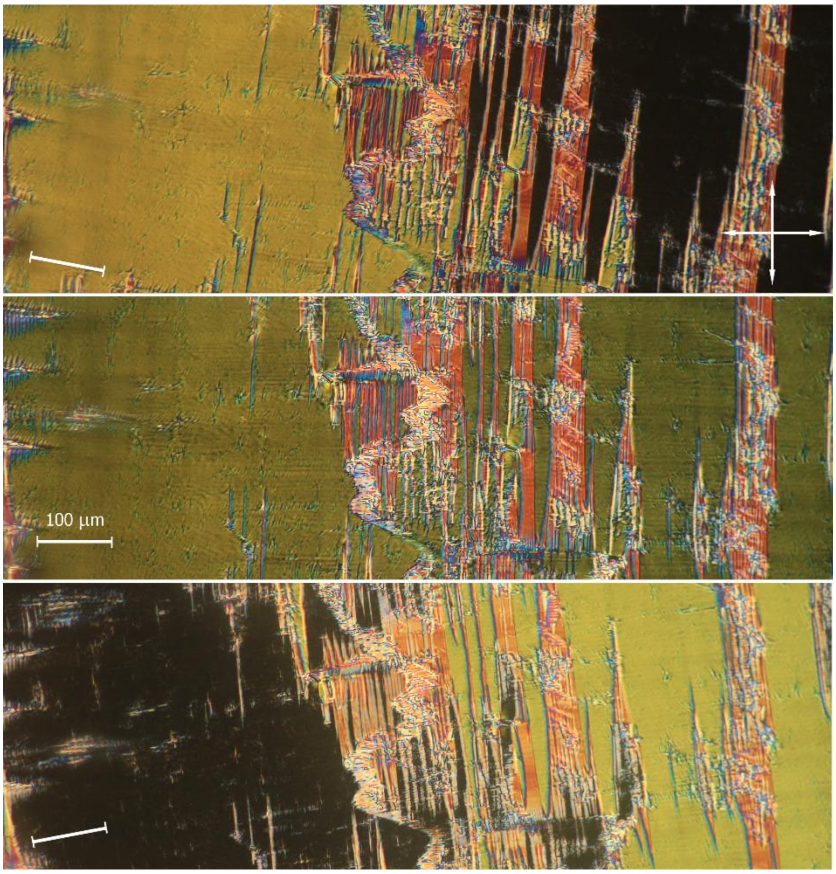

Figure S51: Optical texture of SmC_F phase rac-**RW4*** compound in 5-µm-thick cell with planar anchoring, showing tilted domains which can be brought into extinction by rotating the sample with respect to crossed polarizers (arrows). Scale bar shows the rubbing direction.

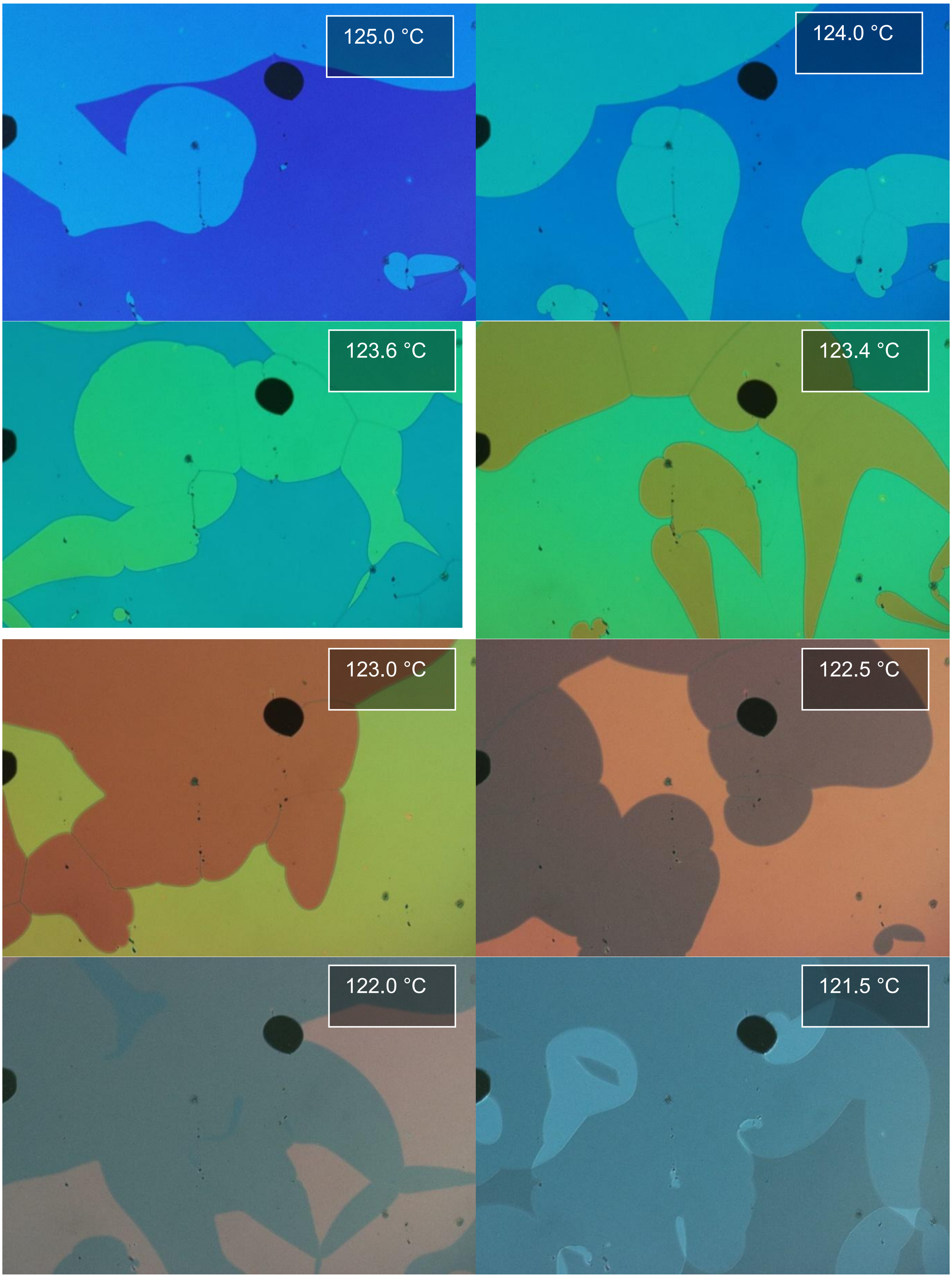

Figure S52: Grandjean textures of N* phase of S-**RW4***, changes of the colours are due to unwinding of the helix.

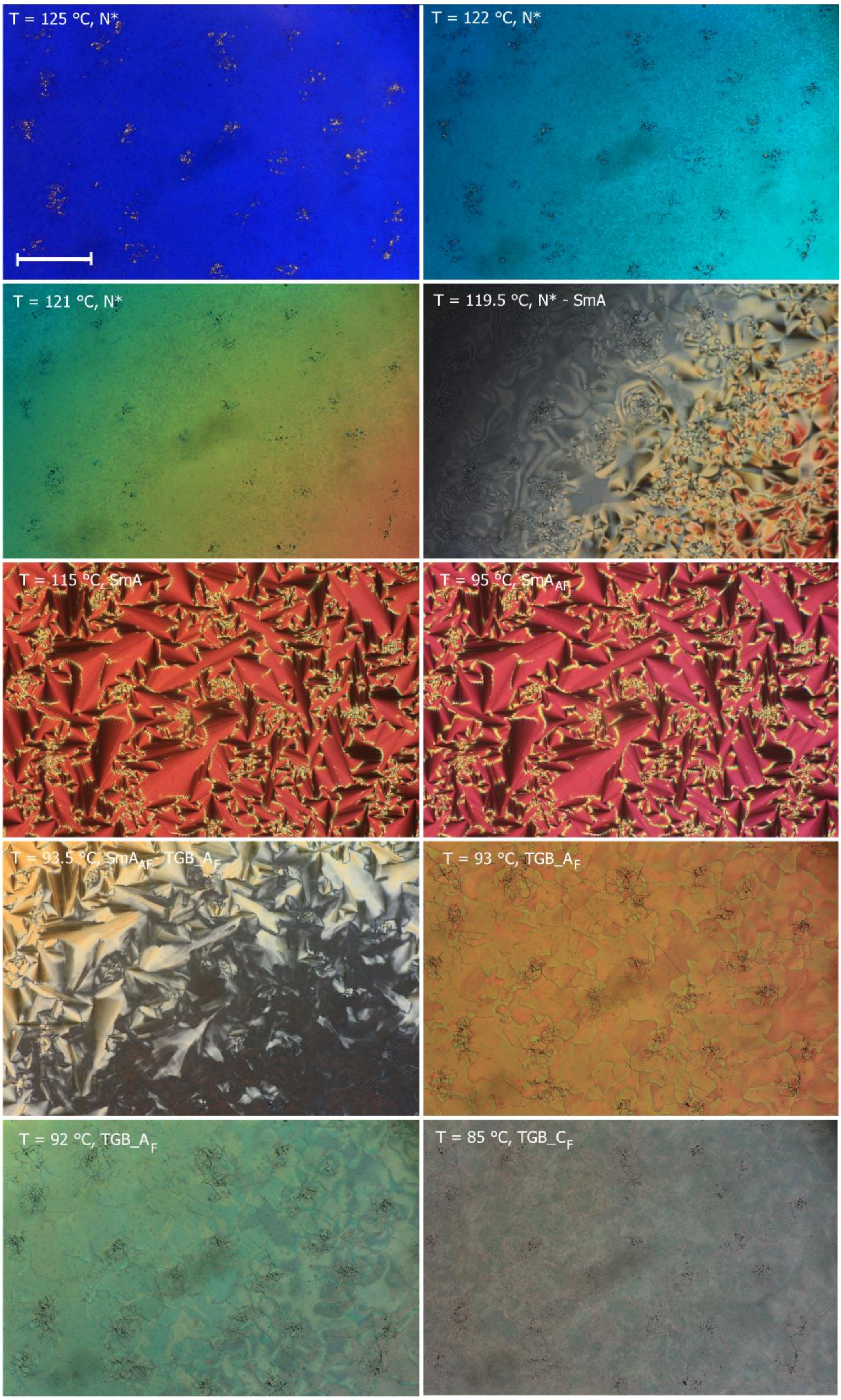

Figure S53: Optical textures of LC phases of S-**RW4*** observed in 3-μm-thick cell with degenerate planar anchoring.

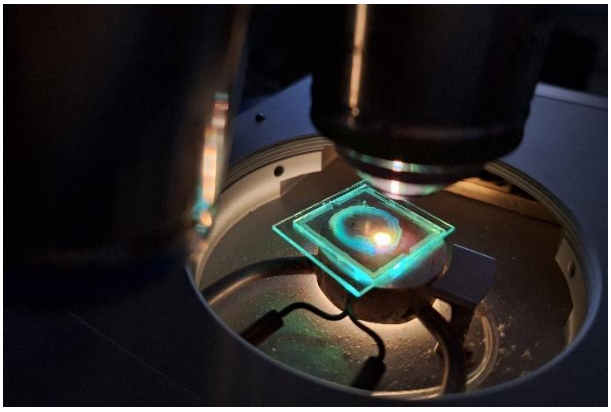

Figure S54: Selective reflection from the sample in TGB_A_F phase.

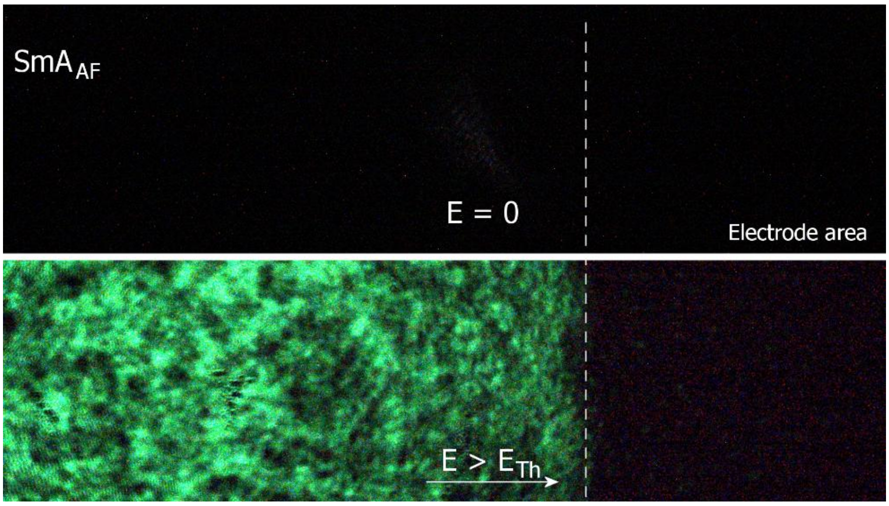

Figure S55: SHG microscopy images recorded in SmA_{AF} phase of rac-**RW4*** in cell with in-plane electrodes. Ground state is clearly SHG silent, while under applied electric field (area on the left from dashed line) strong SHG activity is detected.

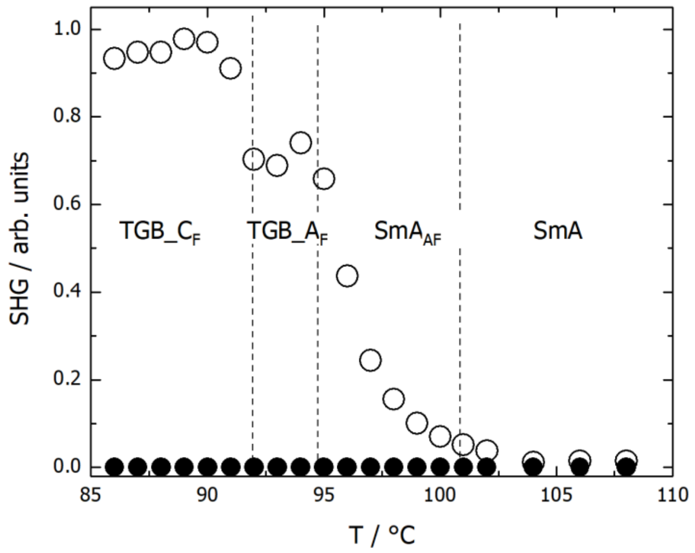

Figure S56: SHG intensity recorded on cooling for S-**RW4*** under applied electric field (open circles) and without electric field (solid circles). Note that the ground state of all the phases is SHG silent, evidencing full compensation of electric polarization.

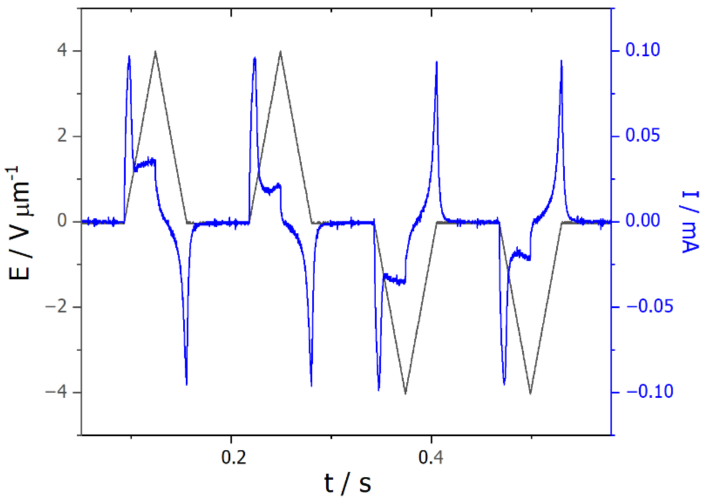

Figure S57: Switching current (blue line) recorded under application of modified triangular-wave voltage (black line) for SmA$_{AF}$ phase of S-**RW4*** compound. Repolarization current peaks appearing at each rise/fall of electric field confirm that the antiferroelectric ground state is restored at zero electric field.

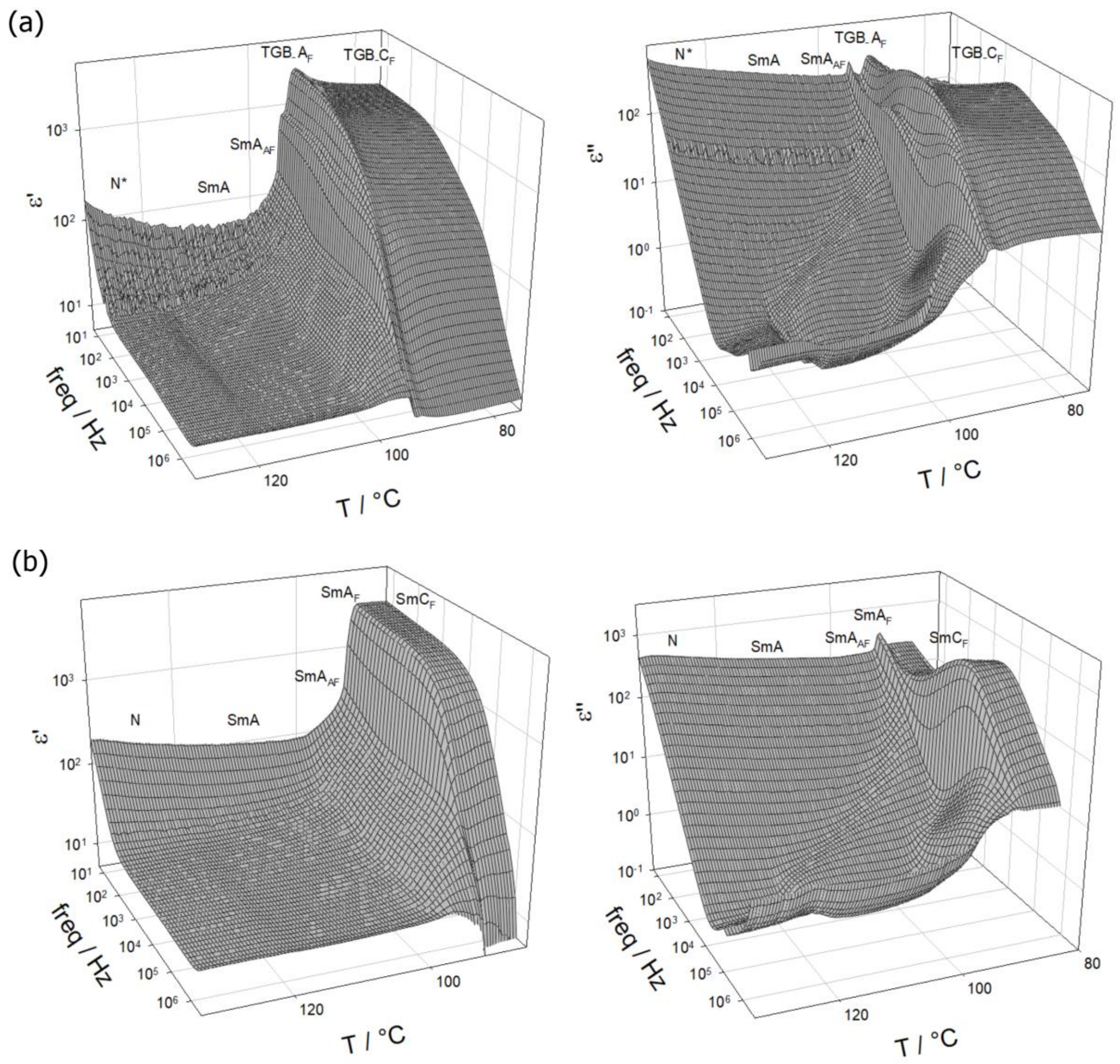

Figure S58: Real and imaginary parts of dielectric permittivity measured in cells with Au electrodes for (a) S-**RW4*** and (b) rac-**RW4***.

Phenomenological Model

To consider in more detail observed phase sequences, we employ a phenomenological model. The bulk free energy density is written as a sum of a nematic (f_n), smectic (f_s) and polar (f_P) contributions, assuming a constant value of the nematic order parameter. The nematic contribution to the free energy density can be expressed as:

$$f_n = \frac{1}{2}K_1\big(\vec{n}(\nabla\cdot\vec{n}) - \vec{S}\big)^2 + \frac{1}{2}K_2(\vec{n}\cdot(\nabla\times\vec{n}) - q_0)^2 + \frac{1}{2}K_3\big(\vec{n}\times(\nabla\times\vec{n})\big)^2 \ , \qquad (1)$$

where $\vec{n}$ is the nematic director and K_1, K_2 and K_3 are the splay, twist and bend elastic constants. $\vec{S} = \gamma\vec{P}/K_1$ is a preferred spontaneous splay due to the flexoelectric effect [4], $\vec{P}$ being a spontaneous polarization and γ a parameter related to the flexoelectric coefficient. If material is chiral, then q_0 is related to the pitch p_0 of the spontaneous twist ($q_0 = 2\pi/p_0$). In the case of a racemic mixture q_0 should be set to zero.

The smectic contribution can be expressed as [5]:

$$f_s = \frac{1}{2}a(T)|\psi|^2 + \frac{1}{4}b|\psi|^4 + c_{\parallel}(T)|(\vec{n}\cdot\nabla - iq_s)\psi|^2 + c_{\perp}|(\vec{n}\times\nabla)\psi|^2 \ , \qquad (2)$$

where ψ is the smectic order parameter. If the smectic layer normal is along the z-direction, then $\psi = \psi_0 e^{iq_s z}$, $q_s = 2\pi/d_s$ and d_s is a smectic layer thickness. The temperature dependent Landau parameter $a(T)$ is negative at $T < T_{NS}$. At $T < T_{NS}$ a smectic phase is stable and the term with Landau parameter b stabilizes a finite magnitude (ψ_0) of the smectic order parameter. The sign of the parameter $c_{\parallel}(T)$ defines the type of the smectic phase. In the SmA phase, $c_{\parallel}(T)$ is positive, and it is negative in the SmC phase, where a finite tilt of the director with respect to smectic layer normal in stabilized by the last term in f_s, with parameter $c_{\perp}$ being positive. At $T = T_{AC}$, $c_{\parallel} = 0$. If $T_{AC} < T_{NS}$, the expression for f_s describes a material with a phase sequence $\mathrm{N} \rightarrow \mathrm{SmA} \rightarrow \mathrm{SmC}$.

Several terms have already been proposed for the free energy density due to the polar ordering [6–9]. Here, we consider only the most relevant ones needed to describe the observed phase sequences. To simplify the estimates, we assume a second order phase transition to the polar phase, thus

$$f_P = \frac{1}{2}\mu(T)P^2 + \frac{1}{4}\nu P^4 + \frac{1}{2}\kappa\big|\nabla\vec{P}\big|^2, \qquad (3)$$

where $\mu(T)$ and ν are Landau parameters defining the transition temperature to the polar state and the term with parameter κ gives the energy penalty for spatial variation in direction and/or magnitude of $\vec{P}$. As pointed out in [9], the splay term in eq. (1) is larger than the sum of the splay elastic and flexoelectric energy contributions by $K_1 S_0^2/2$, where $S_0 = \gamma P_0/K_1$ and P_0 is the equilibrium value of polarization. This should

be accounted for when minimising the free energy for P_0, but it simply leads to a shift of the temperature at which the polar order becomes stable, and we can include this in the temperature dependent Landau term in eq. (3). The equilibrium value of P_0 is found by minimizing the following expression:

$$\frac{1}{2}\mu_0(T-T_P)P_0^2+\frac{1}{4}\nu P_0^4=min, \tag{4}$$

where we used $\mu(T)=\mu_0(T-T_P)$ with $\mu_0>0$. At $T<T_P$ the equilibrium magnitude of polarization is

$$P_0^2=\frac{1}{\nu}\ \mu_0(T_P-T)\ \ . \tag{5}$$

When we plug P_0 from eq. (5) into eq. (3), we obtain $f_P=-\nu P_0^4/4$ if $|\nabla\vec{P}|=0$.

If we assume that $T_P>T_{NS}$, then, at $T<T_P$, the material will first experience the phase transition from the nematic to the ferroelectric nematic phase and by further lowering the temperature the transition to the polar SmA phase and eventually to the polar SmC phase. However, if $T_P<T_{NS}$, the following phase sequence is expected: $\mathrm{N}\rightarrow\mathrm{SmA}\rightarrow\mathrm{SmA_F}\rightarrow\mathrm{SmC_F}$. Because polar phases favour spontaneous splay of polarization, and because the splay cannot be made favourable everywhere, one can also expect smectic structures that will be antiferroelectric but in the sense of antiferroelectricity along the smectic layer (and not perpendicular to it), as shown in Figure S59.

Let us first focus on the racemic mixture, in which the following phase sequence is observed: $\mathrm{N}\rightarrow\mathrm{SmA}\rightarrow\mathrm{SmA_{AF}}\rightarrow\mathrm{SmA_F}\rightarrow\mathrm{SmC_F}$. At temperatures below $T_{P\gamma}$ the polar order becomes stable. However, uniform polar order along the smectic layer in the SmA phase would require the splay of polarization to be zero, which would increase the nematic component of the free energy. Splay of polarization in the smectic phase leads to the undulation of smectic layers, but in the SmA phase this comes at no energy cost, because the smectic layer thickness is preserved. It is also more convenient for the material to be macroscopically apolar, which is obtained by the interchange of domains with the up and down polarization as shown in Figure S59. Such arrangement is easily obtained if the polar order between the two domains is melted. If the width of the melted region is l_w, the energy penalty will be (F_w):

$$F_w=\frac{1}{4}\nu P_0^4\ l_w\ \ . \tag{6}$$

On the other hand, the energy will be gained in the regions of the favourable splay. If there were no splay, the energy in the region of the length l_b would be larger due to the lack of splay but lower because there would be no spatial variation of polarization. The net result which should be compared with the energy of the wall is

$$F_b=\frac{1}{2}K_1S_0^2l_b-\frac{1}{2}\kappa\left(\frac{\Delta P_x}{l_b}\right)^2\ l_b\ \ , \tag{7}$$

where $\Delta P_x = 2P_0 \sin\theta_0$ and θ_0 is the maximum splay angle (see Figure S59). By equating energies given by eqs. (6) and (7), we find

$$\frac{l_b}{l_w} = \frac{\nu K_1 P_0^2}{2\gamma^2}\left(1 - \frac{4\kappa K_1}{\gamma^2 l_b^2}\sin^2\theta_0\right)^{-1} . \qquad (8)$$

In the case of the equilibrium splay $\nabla \cdot \vec{n} = \vec{n} \cdot \vec{P}_0$, from where it follows that $2\sin\theta_0 / l_b = \gamma P_0 / K_1$, so

$$l_b = \frac{2K_1}{\gamma P_0}\sin\theta_0 . \qquad (9)$$

By plugging l_b from eq. (9) into the right-hand-side of eq. (8) we obtain

$$\frac{l_b}{l_w} = \frac{\frac{\nu K_1 P_0^2}{2\gamma^2}}{1 - \frac{\kappa P_0^2}{K_1}} . \qquad (10)$$

From eq. (10) we see that the ratio between the width of the block and width of the wall increases as temperature decreases, because P_0 increases by decreasing temperature, so the numerator in eq. (10) increases, and the denominator decreases. Also, when temperature decreases, the energy of the wall (eq. (5)) increases with $(T_P - T)^2$, while the energy penalty of no or unfavourable splay increases with $(T_P - T)$. As a result, at some temperature the formation of the wall with no polar order will not be favourable anymore. This will lead to the transition to the $\mathrm{SmA_F}$ phase, where the energy of the wall is related to the unfavourable splay (see Figure S59b) and to the spatial variation in polarization as

$$F_w = \frac{1}{2}K_1\left(\frac{\Delta n_x}{l_w} + \frac{\gamma P_0}{K_1}\right)^2 l_w + \frac{1}{2}\kappa\left(\frac{\Delta P_x}{l_w}\right)^2 l_w , \qquad (11)$$

where $\Delta n_x = 2\sin\theta_0$. The transition temperature T_F from the $\mathrm{SmA_{AF}}$ to the $\mathrm{SmA_F}$ phase is obtained by equating eqs. (6) and (11). If we assume that the main temperature dependence is in the magnitude of polarization and that the width of the wall is very weakly dependent on temperature, we obtain a fourth order polynomial equation for P_{0,T_F} – polarization value at temperature $T = T_F$:

$$\frac{1}{2}K_1\left(\frac{2\sin\theta_0}{l_w} + \frac{\gamma P_{0,T_F}}{K_1}\right)^2 + \frac{1}{2}\kappa\left(\frac{2P_{0,T_F}\sin\theta_0}{l_w}\right)^2 = \frac{1}{4}\nu P_{0,T_F}^4 . \qquad (12)$$

From eq. (12) one can find P_{0,T_F} and then estimate T_F from eq. (6), if other parameters are known.

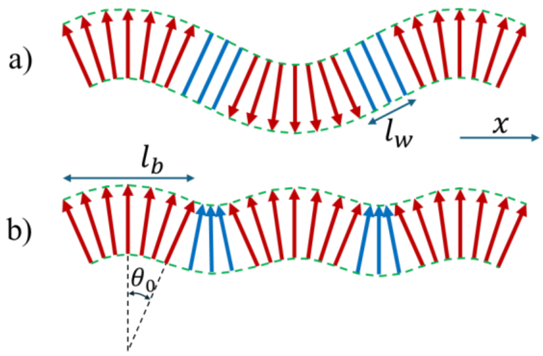

Figure S59. Tendency for the polarization splay leads to undulation of smectic layers in the a) SmA_{AF} and b) SmA_F phase observed in a racemic mixture. Red arrows present the local polarization direction with a favourable splay, blue arrows regions with unfavourable splay, blue rods present the direction of the average molecular long axes in regions with no polar order. The width of the blocks with a favourable splay is l_b, the width of the walls with an unfavourable splay or no polar order is l_w, θ_0 is the angle of the maximum splay along the x – axis.

Now, let us focus on a chiral material. In this case, the material prefers also a spontaneous twist. Spontaneous twist, however, is impossible to accommodate along the smectic layer without changing the thickness of the layer. Upon transition to the polar phase, we first obtain the $\mathrm{SmA_{AF}}$ phase, because the cost of melting the polar order is low at low values of $T_P - T$. In the SmA and $\mathrm{SmA_{AF}}$ phase, the energy density price for no spontaneous twist is $K_2 q_0^2/2$, for $\mathrm{SmA_{AF}}$, both in the block of favourable splay and in the walls with no polar order. When temperature is further reduced below T_F, the system can finally achieve spontaneous twist as well, by a phase transition to the polar $\mathrm{TGB\_}A_F$ phase instead of the $\mathrm{SmA_F}$ phase, where the wall between two regions of favourable splay contains only the unfavourable splay. In a chiral material, the energy of the wall can be reduced by forming a series of screw dislocation in a TGB boundary. Such a boundary contains favourable twist and unfavourable splay, the energy price of the latter being compensated by the energy gain due to the former.

Finally, we estimate the width of the block (l_b). We assume a typical value for the elastic constant $K_1 \approx 2 \times 10^{-11}\mathrm{N}$ and take the value of P_0 at $T \approx 100°\mathrm{C}$, $P_0 \approx 10^{-2}\mathrm{Cm}^{-2}$ (see Figure 4a in the main text). From the measurements of the flexoelectric coefficient $e^*/K \sim 1\ \mathrm{Cm}^{-1}\mathrm{N}^{-1}$ in the nematic phase of material exhibiting the ferroelectric nematic phase [10] and relating it to the "energy" flexoelectric coefficient γ as $\gamma = e^*/(\varepsilon\varepsilon_0)$, with $\varepsilon \sim 100$, we find $\gamma \sim 0.01\ \mathrm{V}$, which agrees with the value chosen for γ in [9]. From the birefringence measurements it can be deduced (see the next section in SI) that the splay amplitude is $\theta_0 \approx 0.025$. The value of l_b is then estimated from eq. (9) and found to be approximately $10\ \mathrm{nm}$. The obtained order of magnitude is consistent with the widths of the blocks measured in the ordinary, non-polar $\mathrm{TGB\_A}$ phase [11].

Estimation of the maximum splay angle

To estimate the maximum splay angle from the measurements of birefringence, we use the following procedure. The dielectric tensor at optical frequencies ($\underline{\varepsilon}$) of a uniaxial material with the optic axis along the z-direction (direction of the smectic layer normal in the case of planar smectic layers in Figure S59) is

$$\underline{\varepsilon} = \begin{pmatrix} \varepsilon_\perp & 0 & 0 \\ 0 & \varepsilon_\perp & 0 \\ 0 & 0 & \varepsilon_\parallel \end{pmatrix} . \tag{13}$$

If the optic axis rotates by θ around the y-axis, the dielectric tensor transforms to

$$\underline{\varepsilon}(\theta) = \begin{pmatrix} \varepsilon_\perp \cos^2\theta + \varepsilon_\parallel \sin^2\theta & 0 & \frac{1}{2}\Delta\varepsilon \sin(2\theta) \\ 0 & \varepsilon_\perp & 0 \\ \frac{1}{2}\Delta\varepsilon \sin(2\theta) & 0 & \varepsilon_\perp \sin^2\theta + \varepsilon_\parallel \cos^2\theta \end{pmatrix} , \tag{14}$$

where $\Delta\varepsilon = \varepsilon_\parallel - \varepsilon_\perp$ is an anisotropy of a uniform phase, in our case the SmA phase. To find anisotropy of a modulated phase, we average $\underline{\varepsilon}(\theta)$ over all θ between $\pm\theta_0$ and find the dielectric tensor ($\underline{\varepsilon}_{splay}$) of the splayed structure as

$$\underline{\varepsilon}_{splay} = \frac{1}{2\theta_0}\int_{-\theta_0}^{\theta_0} \underline{\varepsilon}(\theta) d\theta \ , \tag{15}$$

from where it follows

$$\underline{\varepsilon}_{splay} = \begin{pmatrix} \frac{\varepsilon_\perp + \varepsilon_\parallel}{2} + \frac{\Delta\varepsilon}{4\theta_0}\sin(2\theta_0) & 0 & 0 \\ 0 & \varepsilon_\perp & 0 \\ 0 & 0 & \frac{\varepsilon_\perp + \varepsilon_\parallel}{2} - \frac{\Delta\varepsilon}{4\theta_0}\sin(2\theta_0) \end{pmatrix} . \tag{16}$$

The anisotropy of the splayed structure ($\Delta\varepsilon_{splay}$) is thus

$$\Delta\varepsilon_{splay} = \frac{\Delta\varepsilon}{2\theta_0}\sin(2\theta_0) \ . \tag{17}$$

In the limit of very small splay angle, $\Delta\varepsilon_{splay} = \Delta\varepsilon$, but we are interested in the first nonzero correction to this limit:

$$\Delta\varepsilon_{splay} = \Delta\varepsilon\left(1 - \frac{4}{3}\theta_0^2\right) \ . \tag{18}$$

By using $\Delta\varepsilon = (n_e + n_o)(n_e - n_o) = (n_e + n_o)\Delta n$, where n_e and n_o are the extraordinary and ordinary index of refraction, respectively, and Δn is birefringence, we find that the difference in the birefringence between the splayed and uniform structure is

$$\Delta n_{splay} - \Delta n = -\frac{4}{3}\Delta n\, \theta_0^2 \ . \qquad (19)$$

The maximum splay angle is thus

$$\theta_0^2 = -\frac{3}{4}\frac{\Delta n_{splay} - \Delta n}{\Delta n} \ . \qquad (20)$$

The birefringence is $\Delta n \approx 0.17$ close to the SmA – SmA_{AF} phase transition and $\Delta n_{splay} - \Delta n \approx -1.4 \times 10^{-4}$ (both values can be deduced from Figure 1e in the main text). By plugging these values into eq. (20), we find $\theta_0 \approx 0.025\ \mathrm{rad} \approx 1.4\ \mathrm{deg}$. The total splay is thus approximately $3\ \mathrm{deg}$ (from $-\theta_0$ to θ_0).

References

[1] H. Maebayashi, T. Fuchigami, Y. Gotoh, M. Inoue, Stereoselective Acetalization for the Synthesis of Liquid-Crystal Compounds Possessing a trans-2,5-Disubstituted 1,3-Dioxane Ring with Saturated Aqueous Solutions of Inorganic Salts, *Org. Process Res. Dev.*, **23**, 477, (2019).

[2] S. Brown, E. Cruickshank, J. M. D. Storey, C. T. Imrie, D. Pociecha, M. Majewska, A. Makal, E. Gorecka, Multiple Polar and Non-polar Nematic Phases, *ChemPhysChem*, **22**, 2506, (2021).

[3] G. J. Strachan, E. Górecka, J. Szydłowska, A. Makal, D. Pociecha, Nematic and Smectic Phases with Proper Ferroelectric Order, *Adv. Sci.*, **12**, 2409754 (2025).

[4] N. Sebastián et al., Polarization patterning in ferroelectric nematic liquids via flexoelectric coupling, *Nat. Commun*. **14**, 3029, (2023).

[5] S. R. Renn and T. C. Lubensky, Abrikosov dislocation lattice in a model of the cholesteric – to – smectic- *A* transition, *Phys. Rev. A* **38**, 2132, (1988).

[6] E. I. Kats, Stability of the uniform ferroelectric nematic phase, *Phys. Rev. E* **103**, 012704, (2021).

[7] N. Vaupotič, D. Pociecha, P. Rybak, J. Matraszek, M. Čepič, J. M. Wolska, and E. Gorecka, Dielectric response of a ferroelectric nematic liquid crystalline phase in thin cells, *Liq. Cryst.* **50**, 584, (2023).

[8] L. Paik and J. V. Selinger, Flexoelectricity versus electrostatics in polar nematic liquid crystals, *Phys. Rev. E* **111**, L053402, (2025).

[9] P. Medle Rupnik, E. Hanžel, M. Lovšin, N. Osterman, C. J. Gibb, R. J. Mandle, N. Sebastián, and A. Mertelj, Antiferroelectric Order in Nematic Liquids: Flexoelectricity Versus Electrostatics, *Adv. Science* **12**, 2414818, (2025).

[10] A. Barthakur, J. Karcz, P. Kula, and S. Dhara, Critical splay fluctuations and colossal flexoelectric effect above the nonpolar to polar nematic phase transition, *Phys. Rev. Mater.* **7**, 035603, (2023).

[11] L. Navailles, B. Pansu, L. Gorre-Talini, and H. T. Nguyen, Structural Study of a Commensurate TGB A Phase and of a Presumed Chiral Line Liquid Phase, *Phys. Rev*. *Lett*. **81**, 4168, (1998).
